# Tailoring Reactivity of Molecular Gallium Hydroxides via Chalcogen Bridging

**DOI:** 10.1021/acs.inorgchem.6c03433

**Published:** 2026-09-14

**Authors:** Erandi Bernabé-Pablo, Alfonso Gallardo-Garibay, Vojtech Jancik, Diego Martínez-Otero, Roman Neufeld, Uvaldo Hernández-Balderas, Dietmar Stalke, Mónica Moya-Cabrera

**Affiliations:** † 7180 Universidad Nacional Autónoma de México, Instituto de Química, Ciudad Universitaria, Ciudad de México 04510, México; ‡ Centro Conjunto de Investigación en QuímicaSustentable UAEM-UNAM, Carretera Toluca-Atlacomulco Km 14.5, Toluca, Estado de México 50200, México; § Institut für Anorganische Chemie, Georg-August Universität Göttingen, Tammannstrasse 4, 37077 Göttingen, Germany

## Abstract

We studied the influence of bridging chalcogen atoms (μ-E; E = S, Se) on the structure and reactivity of digallium dihydroxide complexes [{^Me^LGa­(OH)}_2_(μ-E)] [E = S (**4**), Se (**5**); ^Me^L = CH­{CMe­(NAr)}_2_^–^, Ar = 2,4,6-Me_3_C_6_H_2_]. Compounds **4** and **5** were synthesized from ^Me^LGaCl_2_ and sodium chalcogenide (Na_2_E) in THF-NH_3_(*l*), followed by controlled hydrolysis. For the sulfur system, the intermediates [{^Me^LGa­(Cl)}_2_(μ-S)] (**2**) and [{^Me^LGa­(NH_2_)}_2_(μ-S)] (**3**) were isolated and characterized, whereas the corresponding selenium derivatives proved too reactive for isolation. The reactivity of **4** and **5** toward Group 4 metal amides, M­(NR_2_)_4_ (M = Ti, R = Me; M = Zr, Hf, R = Et), revealed a chalcogen effect, affording both monocyclic and spirocyclic complexes from **4**, while **5** selectively yielded spirocyclic complexes. Likewise, the milder amide Me_2_Si­(NMe_2_)_2_ reacted exclusively with **5** to produce a siloxide-bridged derivative. Distinct borane-dependent pathways were also observed. Treatment of **4** and **5** with BH_3_·THF resulted in the Ga–OH protonolysis to form cyclic borates, whereas BH_3_·SMe_2_ promoted hydride attack at gallium, yielding gallium hydrides. These results provide an insight into the ambiphilic character of **4** and **5** and establish chalcogen bridging as a strategy for tuning their bonding and reactivity.

## Graphical abstract

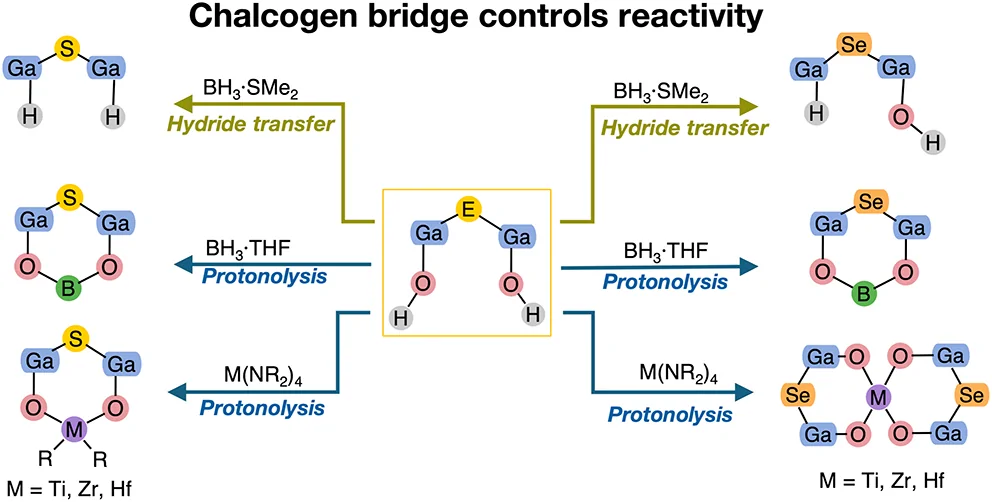

## Introduction

The chemistry of Group 13 metal complexes continues to offer new opportunities for the development of functional materials and homogeneous catalysts.^1–6^ Although this area has traditionally been dominated by aluminum complexes due to their high reactivity,^7–9^ gallium complexes have emerged as an attractive alternative. Despite their nearly identical covalent radii (1.22 Å for Ga vs 1.21 Å for Al),^10^ gallium is less Lewis acidic and less electrophilic than aluminum. Consequently, gallium compounds generally show greater resistance toward hydrolysis and a stronger tendency to form discrete, soluble complexes with oxygen-containing ligands, features that can be advantageous in a variety of applications. Moreover, gallium systems exhibit reactivity patterns and catalytic activities that often rival, and in some cases surpass, those of their aluminum counterparts.^11–21^ Nonetheless, the broader development of gallium chemistry remains hindered by synthetic challenges associated with the lower reactivity of Ga–X and Ga–H bonds in potential precursors relative to the corresponding aluminum species, making it difficult to extend aluminum chemistry to gallium analogs.^22,23^

In this regard, our group has previously reported the synthesis of dinuclear aluminum chalcogenides [{^Me^LAl­(EH)}_2_(μ-E)] (E = S, Se; ^Me^L = CH­{CMe­(NAr)}_2_^–^, Ar = 2,4,6-Me_3_C_6_H_2_), as well as the alumoxane dihydroxide [{^Me^LAl­(OH)}_2_(μ-O)], derived from ^Me^LAlH_2_, and subsequently used as versatile precursors for heterometallic systems.^24,25^ Attempts to access analogous gallium species using a similar strategy were largely unsuccessful, mainly due to the lower electrophilicity of the Ga–H bond.^23^ As an alternative, the galloxane dihydroxide [{^Me^LGa­(OH)}_2_(μ-O)] (**I**) was synthesized via hydrolysis of ^Me^LGaCl_2_ (**1**) in the presence of a strong base, namely the N-heterocyclic carbene, 1,3-di-*tert*-butylimidazol-2-ilydene (I^*t*^Bu, Scheme 1a).^23^

**Scheme 1:**
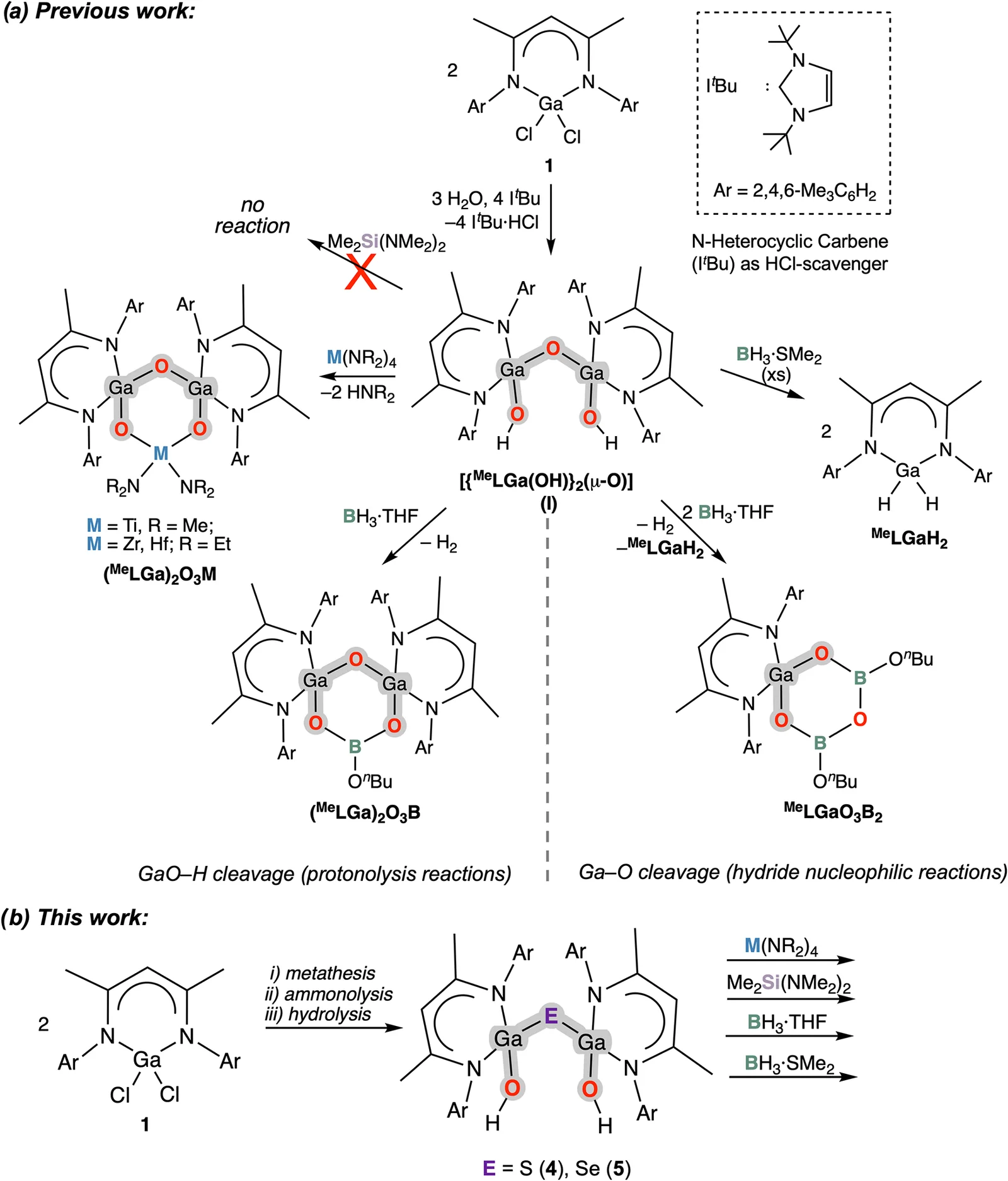
(a) Synthesis and Representative Reactivity of Galloxane Dihydroxide **I**, Illustrating Its Ambiphilic Behavior upon Treatment with Boron Hydrides and Selected Amides; (b) General Synthetic Reactions for Digallium Chalcogenide Dihydroxides **4** and **5** Investigated in This Work

Compound **I** enabled a detailed investigation of Ga–OH reactivity, revealing its dual (ambiphilic) character through both protonolysis and nucleophilic hydride-type reactivity at the gallium centers (Scheme 1a).^23,26^ The Brønsted acidity of the GaO–H groups allowed the incorporation of Group 4 metal amides to afford **(** ^**Me**^ **LGa)**_**2**_ **O**_**3**_ **M** (M = Ti, R = Me; M = Zr, Hf, R = Et) through protonolysis reactions.^23^ Galloxane **I** underwent clean protonolysis with BH_3_·THF, yielding **(** ^**Me**^ **LGa)**_**2**_ **O**_**3**_ **B**, but in the presence of an excess of borane, hydride nucleophilic attack on the gallium center promoted the cleavage of the Ga–O–Ga unit, producing ^**Me**^ **LGaO**_**3**_ **B**_**2**_ and ^**Me**^ **LGaH**_**2**_. The O^*n*^Bu substituent groups in borate products originate from the ring-opening reaction of the THF molecule coordinated to the borane. Moreover, treatment of **I** with excess of BH_3_·SMe_2_ led to ^**Me**^ **LGaH**_**2**_ through a hydride attack on both gallium centers.^26^

These compounds, accessed through the ambiphilic reactivity of **I**, inspired the development of related systems in which the properties of the Ga–O–Ga units could be deliberately tuned. Despite growing interest in molecular hydroxides, strategies for modulating the ambiphilic character of OH functionalities remain largely unexplored, leaving this aspect of main group hydroxo chemistry underdeveloped. Thus, building on insights from [{^Me^LGa­(OH)}_2_(μ-O)] (**I**), we envisioned replacing the bridging oxygen atoms with S and Se to alter the environment of the hydroxide groups. To the best of our knowledge, the influence of bridging chalcogen atoms (S and Se) on the bonding and reactivity of Ga–OH-containing systems has not been previously investigated.

Unsurprisingly, reports on discrete compounds featuring the Ga–E–Ga unit are limited due to the gallium chalcogenides’ propensity to form oligomeric structures as well as the limited availability of suitable starting materials. Examples of fully characterized compounds of gallium chalcogenides with low aggregation order (*n* ≤ 2, where *n* = oligomeric aggregation number) remain scarce (compounds **a**–**m**; Figure 1).^27–38^ The synthetic routes reported for these gallium chalcogenides are the direct reaction of derivatives of subvalent Ga^I^ with S_8_ (**b**, **c**, **l**, **i**),^27–31^ or Ga^II^ with Et_3_PE (**e**),^32^ as well as the reaction of gallium dichlorides with Li_2_E (**a**, **h**, **k**),^33,34^ and other gallium derivatives through a particular pathway in the presence of S_8_ (**g**, **j**),^35,36^ PhNCS (**f**, **m**)^37^ or UV radiation (**d**).^38^ In these examples, the use of sterically demanding ligands was essential to suppress oligomerization and form discrete Ga=E and Ga–E–Ga motifs. Nonetheless, the incorporation of hydroxide functionality into such frameworks remains largely unexplored. The only reported structure with a S–Ga–OH unit corresponds to the partially hydrolyzed ionic diphenyl bis-thiosemicarbazone gallium nitrate complex, where the sulfur atom serves as a κ^2^-bridge, and the hydroxide functions as a μ–OH bridging the gallium atoms,^39^ whereas examples for selenium are virtually unknown.

**Figure 1:**
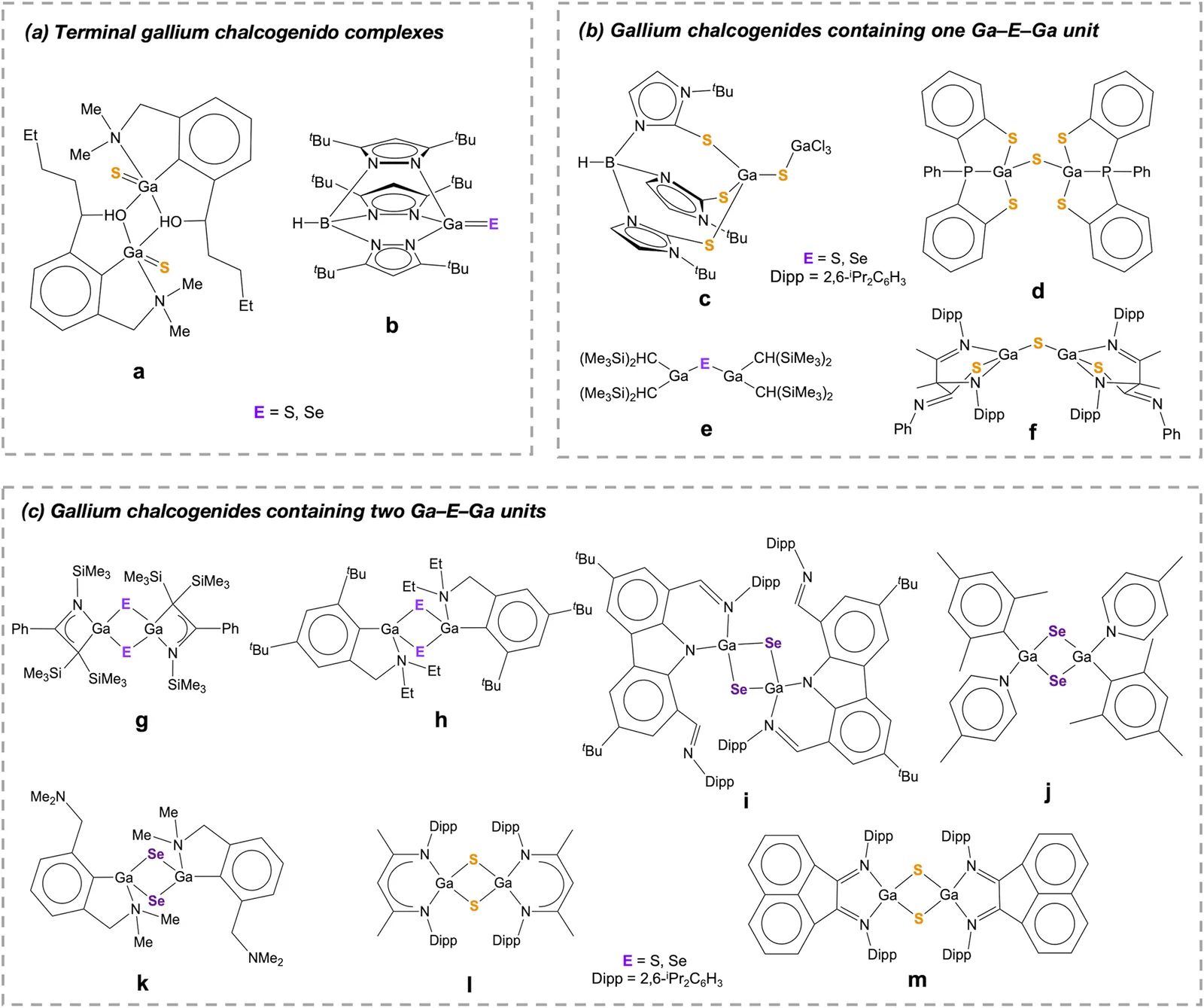
Discrete gallium chalcogenide complexes reported in the literature: (a) Terminal gallium chalcogenido complexes, (b) acyclic gallium chalcogenides containing one Ga–E–Ga unit, (c) dimeric gallium chalcogenide complexes. The structures shown represent all currently known examples.

Moreover, monomeric gallium complexes featuring a terminal Ga–OH unit have been stabilized by the bulkier β-diketiminate ligand ^*i*Pr^L (^*i*Pr^L = CH­{CMe­(NDipp)}_2_^–^; Dipp = 2,6-^*i*^Pr_2_C_6_H_3_), which is closely related to the ligand employed in this work. Representative examples include ^*i*Pr^LGa­(OH)­R′ (R′ = OH),^40^ Me,^41^ OCH_2_C≡CH,^42^ [(μ-O)­Si­(OH)­(O^*t*^Bu)_2_],^43^ PP­(H)­{N­(Dipp)­CH_2_}_2_,^44^ [μ-η^1^:η^2^-P_6_H­(Ga^*i*Pr^L)],^45^ η^1^(C)-*o*-pyridyl, κ^2^-*O,C-o*-oxopyridyl, OC_6_H_9_, κ^2^-*O,O*′-O_2_CPh_2_, CH_2_S­(=O)­Me, and CH­(Me)­P­(=O)­Et_2_.^46^ In most cases, the hydroxide group originates from the reaction of the corresponding gallium precursor with water.^40–45^ A notable exception was reported by Schulz and co-workers, in which treatment of ^*i*Pr^LGa with N_2_O in the presence of pyridine or pyridine N-oxide led to hydroxide formation via a sequence involving oxidation and intramolecular C–H activation.^46^ Despite these advances, examples of molecular gallium hydroxides containing more than one Ga–OH functionality remain extremely rare. To the best of our knowledge, the only structurally characterized bimetallic gallium hydroxide bearing two Ga–OH groups connected through a μ-O bridge is [{^Me^LGa­(OH)}_2_(μ-O)] (**I**).

Herein, we report on an approach towards the syntheses of digallium chalcogenide dihydroxides [{^Me^LGa­(OH)}_2_(μ-E)] [E = S (**4**), Se (**5**)] (Scheme 1b), along with a systematic study of their divergent reactivity patterns towards boranes, silicon amides, and Group 4 amides. Collectively, these results suggest that chalcogen bridging is an effective design element for tuning reactivity within gallium hydroxide frameworks, providing a platform for the development of an alternative class of bifunctional main-group complexes.

## Results and Discussion

### Synthesis of Digallium Chalcogenide Hydroxides

The synthesis of the Ga–S–Ga motif was achieved by treating the gallium dichloride precursor ^Me^LGaCl_2_ (**1**) with Na_2_S prepared *in situ* from elemental sulfur and sodium metal in a THF-NH_3_(*l*) medium at −50 °C. This reaction afforded the sulfur-bridged digallium dichloride [{^Me^LGa­(Cl)}_2_(μ-S)] (**2**) in 49% yield (Scheme 2). Notably, the successful preparation of **2** required the use of freshly prepared, rigorously anhydrous Na_2_S.

**Scheme 2:**
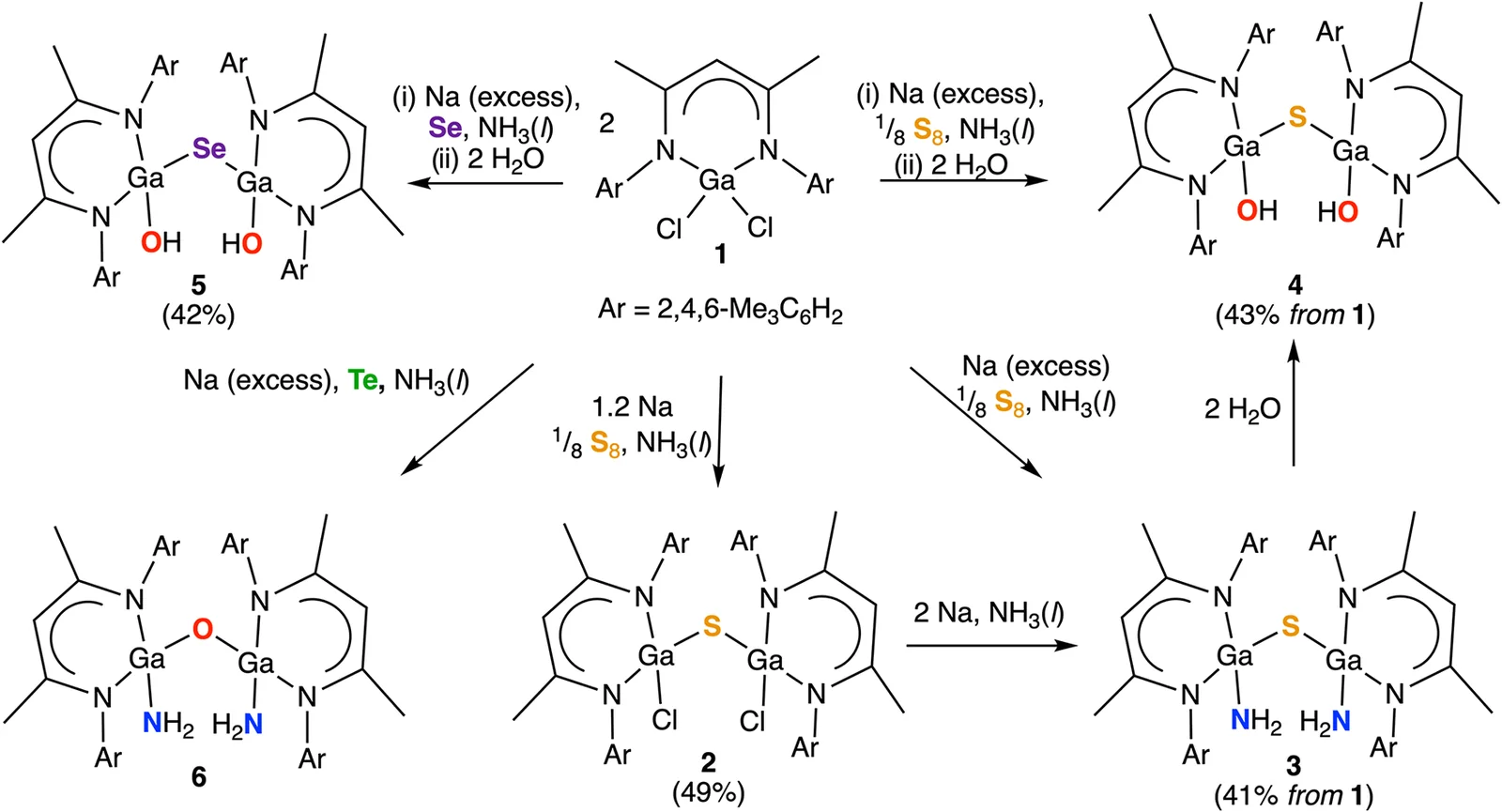
Synthetic Routes to Functionalized Ga–E–Ga (E = S, Se) Compounds **2**–**6**

Despite the ostensibly anhydrous reaction conditions, trace amounts of digallium sulfide dihydroxide [{^Me^LGa­(OH)}_2_(μ-S)] (**4**) were isolated as single crystals. The presence of **4** initially suggested that it might arise from the hydrolysis of **2** accompanied by HCl elimination. To evaluate this possibility, compound **2** was subjected to controlled hydrolysis experiments in the presence of HCl-scavenging bases (I^*t*^Bu^23^ or Proton Sponge). However, no formation of **4** was observed under any of the conditions examined. These results suggest that **4** is not formed via direct hydrolysis of **2**, but rather through an alternative reaction pathway taking place during the synthesis.

In this context, the conversion of Ga–Cl bonds to Ga–NH_2_ functionalities has been shown to occur in NH_3_(*l*) in the presence of a strong base.^40^ Accordingly, we propose that compound **2** undergoes ammonolysis in NH_3_(*l*) to form the diamide [{^Me^LGa­(NH_2_)}_2_(μ-S)], with NH_4_Cl as by-product (Scheme S1, eq 1). Concurrently, sodium dissolves in NH_3_(*l*) to produce solvated electrons (sodium electride), which reacts with the NH_4_Cl to regenerate NH_3_ with the concomitant formation of NaCl and H_2_ (Scheme S1, eq 2). Therefore, after adjusting the Na equivalents, compound [{^Me^LGa­(NH_2_)}_2_(μ-S)] (**3**) was isolated in 41% yield starting from **2** and in 43% yield starting from **1** (Scheme 2). Subsequent treatment of **3** with two equivalents of H_2_O resulted in the clean conversion to the corresponding dihydroxide **4**, thus confirming **3** as a viable intermediate in the formation of **4**. In contrast, reaction of **2** with NaNH_2_ in THF-NH_3_(*l*) at −50 °C led to no observable conversion, suggesting that the formation of **3** under the sodium-mediated conditions cannot be simply attributed to the presence of NaNH_2_.

The synthetic methodology was subsequently extended to selenium, providing the dihydroxide [{^Me^LGa­(OH)}_2_(μ-Se)] (**5**) in 42% yield under condition similar to those employed for sulfur (Scheme 2). However, in contrast to the sulfur system, attempts to isolate selenium-containing intermediates analogous to **2** and **3** were unsuccessful, resulting in insoluble materials.

The reactivity observed with tellurium differed significantly. Treatment of **1** with freshly prepared Na_2_Te in THF-NH_3_(*l*) in the presence of excess sodium afforded [{^Me^LGa­(OH)}_2_(μ-O)] (**I**) as the major product. Efforts to isolate tellurium-containing intermediates resulted instead in the formation of only a small amount the galloxane diamide [{^Me^LGa­(NH_2_)}_2_(μ-O)] (**6**) (5%) which was identified crystallographically and isolated in 5% yield, together with a gray precipitate (Scheme 2). Repeating the reaction under rigorous anhydrous conditions produced an intractable mixture of products, and the resulting species exhibited limited stability in solution, as evidenced by the precipitation within 48 h. The preferential formation oxygen-bridged species **I** and **6**, rather than the tellurium bridged derivatives, suggests that the putative gallium–tellurium species are intrinsically unstable under the employed reaction conditions.

Compounds **2**–**6** are highly moisture-sensitive crystalline solids that are readily soluble in THF, benzene, and toluene. While compounds **4** and **5** are stable in the solid state, they undergo gradual decomposition in solution over several weeks, leading to the formation of insoluble materials. X-ray diffraction analysis revealed that some of these decomposition products correspond to cyclic gallium chalcogenides (*vide infra*), which, once formed, are insoluble in common organic solvents.

The IR (ATR) spectrum of **4** exhibits a weak band at *ṽ* 3616 cm^–1^, attributed to the GaO–H stretching vibration. In compound **5**, a similar band appears at *ṽ* 3614 cm^–1^, along with a weak broad band at *ṽ* 3388 cm^–1^, which is consistent with the OH group involved in hydrogen bonding. This assignment is confirmed by the molecular structure of **5**, determined by X-ray diffraction analysis (*vide infra*).

In contrast, the IR spectra of **3** and **6** display two bands corresponding to the symmetric and asymmetric stretching vibrations of the NH_2_ groups at *ṽ* 3409 and 3325 cm^–1^.

The ^1^H NMR spectra of compounds **2**–**6** display the characteristic hallmarks attributed to Ga–E–Ga species,^23^ showing two sets of signals for the *ortho*-methyl (*o*-Ar–Me) groups and *meta*-hydrogen (*m*-Ar–H*)* atoms of the ligand framework. The OH groups appear as sharp resonances at δ −0.45 (**4**) and −0.31 (**5**) ppm in C_6_D_6_, while the Ga–NH_2_ moieties appear as a broad signal at δ −0.90 (**3**) and −1.13 (**6**) ppm. The pronounced upfield shifts of these NMR signals are attributed to the paramagnetic shielding effects exerted by the nearby aromatic rings.^47^

X-ray diffraction studies were performed on single crystals isolated from saturated solutions of **2**–**6**, and their molecular structures are shown in Figure 2. Compound **2** crystallized with one molecule of benzene and an O/Cl occupation with a 0.13:1.87 ratio. During the crystallization of compound **4**, single crystals of the four-membered GaOGaS species [{^Me^LGa}_2_(μ-S)­(μ-O)] (**7**) (SI) were also isolated after several weeks. Compound **5** proves to be unstable in solution after 78 h and crystallized as a mixture comprising **5** (11.7%), [{^Me^LGa}_2_(μ-Se)_2_] (**8**) (87.6%), and [{^Me^LGa}_2_(μ-Se)­(μ-Se_2_)] (**8a**) (0.7%) (SI).

**Figure 2:**
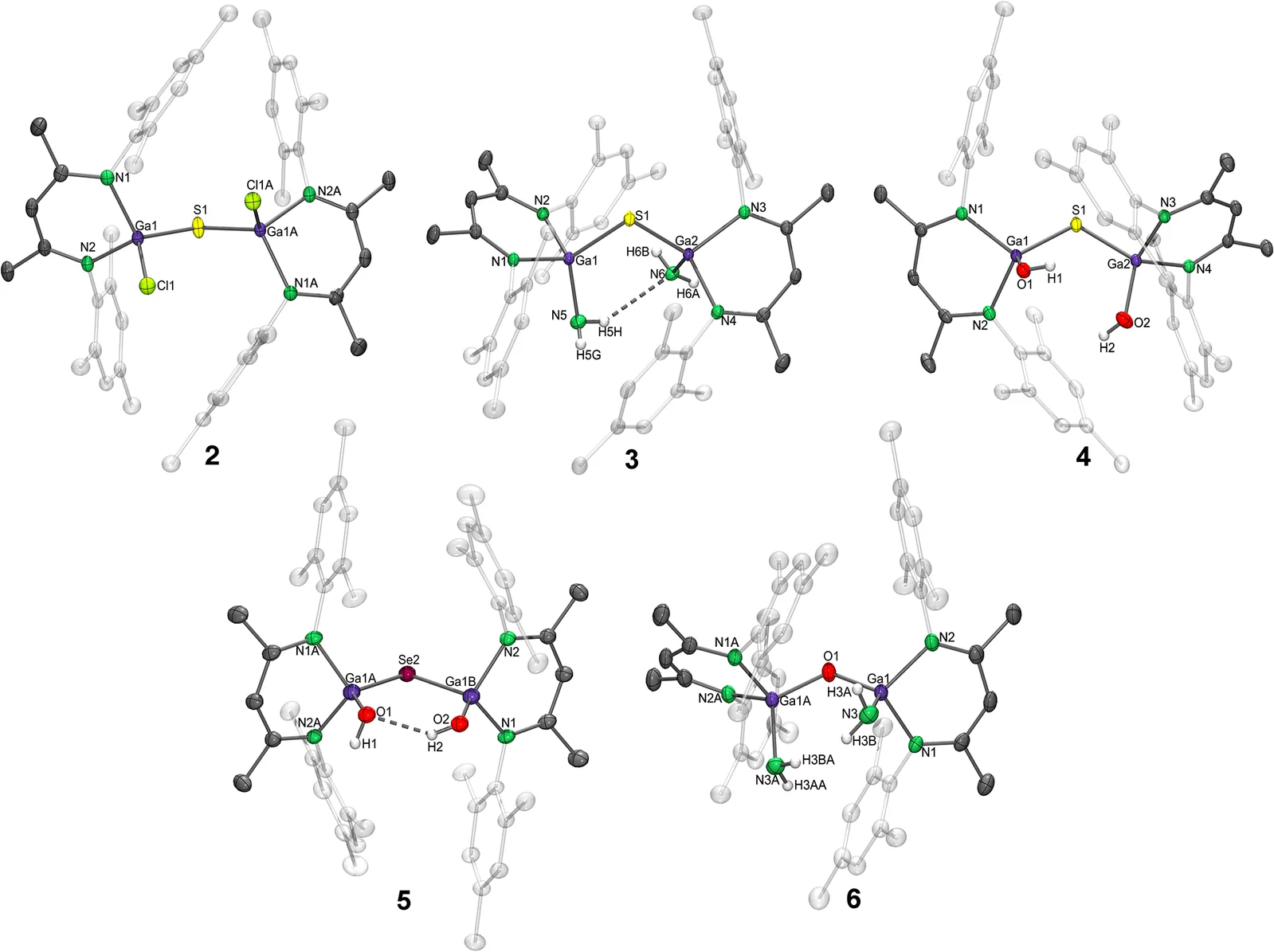
Molecular structures of compounds **2**–**6** with thermal ellipsoids at 50% probability. Hydrogen atoms (except for those in the OH and NH_2_ groups), and solvent molecule for **2**, were omitted for clarity.

The gallium atoms in compounds **2**–**6** adopt a slightly distorted tetrahedral geometry, and the terminal substituents exhibit a *syn* conformation. The angles defined by the X–Ga–E and E–Ga–X planes [X = Cl (**2**), NH_2_ (**3** and **6**), and OH (**4**)] are 50.7°, 44.4°, 43.6°, and 49.1° for compounds **2**, **3**, **4**, and **6**, respectively. For compound **5** (X = OH), this angle is significantly smaller at 6.1°, which results from the intramolecular hydrogen bonding between O1 and H2.

The Ga–S bond lengths in **4** [2.216(1) and 2.223(1) Å] are similar to those observed in compounds **2** [2.196(1) Å] and **3** [2.243(1) and 2.218(1) Å], while the Ga–Se bond lengths in **5** correspond to 2.337(4) and 2.351(4) Å. In **6**, the Ga–(μ-O) bond length and Ga–O–Ga angle [1.818(1) Å, 119.1(2)°], are longer and more acute than in [{^Me^LGa­(OH)}_2_(μ-O)] (**I**) [1.794(1) Å, 126.4(1)°].^23^ This is attributed to the lower electronegativity and greater steric bulk of the NH_2_ groups in **6**. The Ga–NH_2_ bond lengths in **3** [1.866(1), 1.872(1) Å] and **6** [1.878(2) Å] closely match those in the monometallic gallium complex ^*i*Pr^LGa­(^*t*^Bu)­(NH_2_) [1.878(2) Å].^40^

Comparison of the Ga–OH bond lengths and Ga–E–Ga angles in [{^Me^LGa­(OH)}_2_(μ-O)] (**I**) [1.845(1) Å, 126.4(1)°],^23^ **4** [1.815(1) and 1.814(1) Å, 101.8(2)°], and **5** [1.778(18) and 1.817(19) Å, 95.4(2)°] reveals that both the Ga–OH bond lengths and Ga–E–Ga angles decrease as the bridging chalcogen atom (μ-E) becomes heavier. Thus, the decreasing O···O distance (3.434 Å for **I**, 3.335 Å for **4**, and 2.687 Å for **5**) is a direct geometric consequence of the progressive angular contraction Ga–E–Ga down the chalcogenide series. The acute Ga–E–Ga angle provides the necessary geometric arrangement for hydrogen bond formation in **5**. This observation is further supported by non-covalent interaction (NCI) calculations (see Supporting Information for details).

### Reactivity of **4** and **5** with Group 4 Metal- and Silicon-Amides

The structural arrangement of the digallium chalcogenide dihydroxides [{^Me^LGa­(OH)}_2_(μ-E)] [E = S (**4**), Se (**5**)] makes them attractive precursors for the construction of cyclic heterobimetallic systems. Accordingly, the reactivity of **4** and **5** towards Group 4 amides M­(NR_2_)_4_ (M = Ti, R = Me; M = Zr, Hf, R = Et) was investigated to evaluate the influence of the bridging chalcogen (μ-E) on the reactivity of the Ga–OH group and to allow comparison with the behavior of the oxo-bridged analog **I** (Scheme 1a).

Treatment of compound **4** with a toluene solution of M­(NR_2_)_4_ in a 1:1 molar ratio at −79 °C afforded, after workup, heterobimetallic monochelate species of the type [{(^Me^LGa)_2_(μ-S)}­(μ-O)_2_{M­(NR_2_)_2_}] [M = Ti, R = Me (**9**); M = Zr (**10**), Hf (**11**), R = Et] in moderate yields (41–68%). Reversing the order of reagent addition (adding M­(NR_2_)_4_ to a toluene solution of **4** under the same stoichiometry and temperature) led to the isolation of heterobimetallic bischelate complexes [{(^Me^LGa)_2_(μ-S)}­(μ-O)_2_]_2_M [M = Ti (**12**), Zr (**13**), Hf (**14**)] in 42–45% yield (Scheme 3a). Increasing the molar ratio of **4** to favor the formation of bischelate did not improve the yield; instead, additional side products were observed, complicating the isolation of **12**–**14**. These results suggest that **4** engages in competing side reactions with byproducts such as R_2_NH, likely contributing to shifts in the product equilibria.

**Scheme 3:**
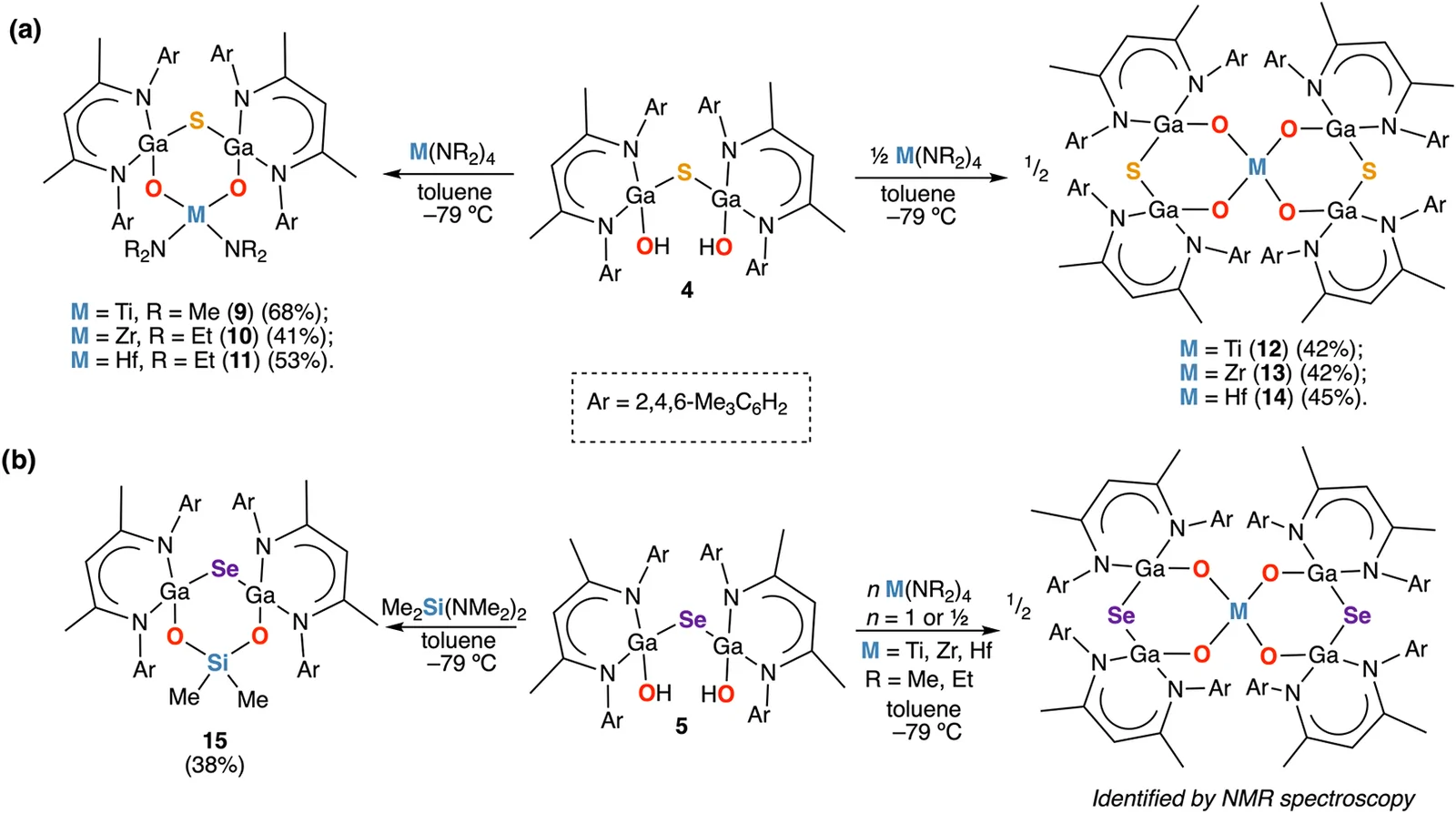
(a) Heterobimetallic Compounds Derived from **4** with M­(NR_2_)_4_; (b) Heteronuclear Compounds Derived from **5** with M­(NR_2_)_4_ and Me_2_Si­(NMe_2_)_2_.

In contrast, the reaction of digallium selenide dihydroxide **5** with M­(NR_2_)_4_ (M = Ti, R = Me; M = Zr, Hf, R = Et) in different molar ratios (1:1 and 2:1) resulted in inseparable mixtures of products, among which the bischelate species [{(^Me^LGa)_2_(μ-Se)}­(μ-O)_2_]_2_M; M = Ti, Zr, Hf] were identified. Attempts to isolate these complexes in their pure form were unsuccessful due to the formation of complex mixtures of side-products. Given the high reactivity of compound **5** with these metal amides, we investigated its reactivity with the milder base bis­(dimethylamino)­dimethylsilane, Me_2_Si­(NMe_2_)_2_. This reaction afforded the cyclic digallium selenide dimethylsiloxide [{(^Me^LGa)_2_(μ-Se)}­(μ-O)_2_{SiMe_2_}] (**15**) in 38% yield (Scheme 3b). Conversely, the reaction of compound **4** with Me_2_Si­(NMe_2_)_2_ under identical conditions resulted in the recovery of the starting material.

Compounds **9**–**15** are air-sensitive solids that exhibited high solubility in aromatic and hydrocarbon solvents, which contributes to their modest isolation yields. The IR spectra of **9**–**15** show no absorption band corresponding to the GaO–H stretching vibration, and their ^1^H NMR spectra display the disappearance of the signal at δ −0.45 ppm for **9**–**14** and at δ −0.31 ppm for **15**, attributed to the OH groups. Notably, the ^1^H NMR spectrum of **9** exhibits a singlet at δ 2.99 ppm, corresponding to the Ti­(NMe_2_)_2_ moiety, while the M­(NEt_2_)_2_ signals for **10** appear at δ 3.10 and 1.23 ppm, and for **11** at δ 3.11 and 1.24 ppm. These signals are absent from the ^1^H NMR spectra of compounds **12–14**, which is consistent with their spirocyclic structures. In contrast, the ^1^H NMR spectrum of **15** displays a signal for methyl groups at δ −0.07 ppm, indicative of the SiMe_2_ group.

The high solubility of the cyclic heterometallic systems in common organic solvents, together with their moisture sensitivity, hindered the isolation of single crystals of **10**, **11**, and **14**. Nonetheless, suitable single crystals of **9** and **12** were obtained after several weeks from concentrated toluene solutions and were characterized by X-ray diffraction studies (Figure 3). A detailed comparison of structural parameters (Ti–O) bond distances, Ga–O–Ti, and O–Ti–O angles, with those of the related complex [{(^Me^LGa)_2_(μ-O)}­(μ-O)_2_{Ti­(NMe_2_)_2_}][Bibr ref23] is provided in the SI.

**Figure 3:**
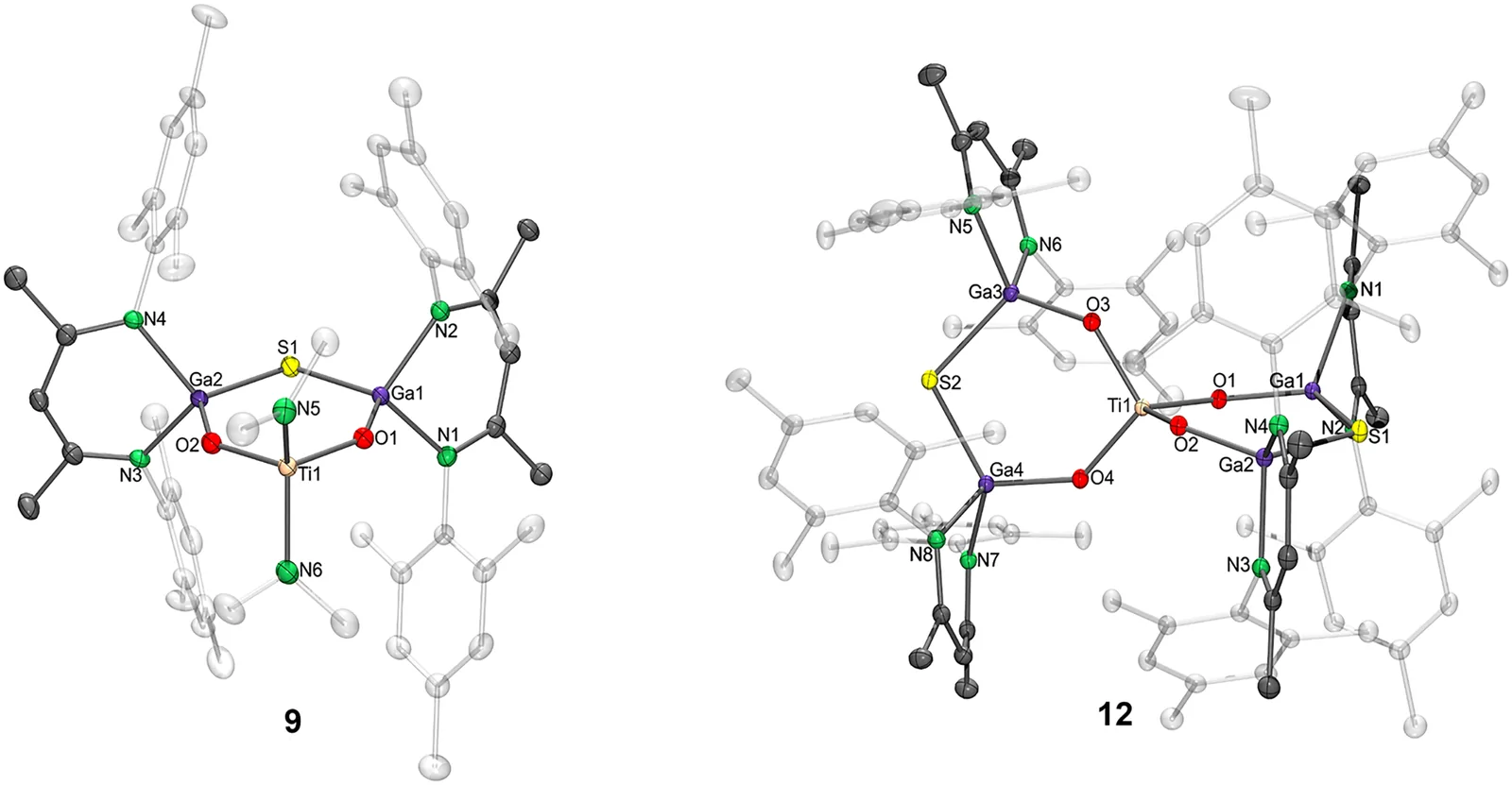
Molecular structures of **9** and **12** with thermal ellipsoids at 50% probability. Hydrogen atoms were omitted for clarity.

For compound **13**, only low-quality single-crystal X-ray diffraction data were obtained, allowing the elucidation of a connectivity model. Compound **13** co-crystallized with the partially hydrolyzed derivative [{(^Me^LGa)_2_(μ-S)}_2_(μ-O)_3_(μ–OH)_2_]­Zr·H_2_O (**13a**) (SI). This species is likely formed from the hydrolysis of **13** with two equivalents of H_2_O, followed by the coordination of an additional H_2_O molecule to the zirconium center.

Additionally, needle-shaped crystals obtained from a saturated toluene solution of **13** were identified by X-ray diffraction studies as the heterobimetallic polyoxocluster [{[{(^Me^LGa)_2_(μ-S)}­(μ-O)_2_Zr]_2_(μ–OH)}_2_(μ–OH)_4_(μ_3_–OH)_2_(μ_3_-O)_2_]Zr (**16**) (SI). The formation of **16** is proposed to occur through the reaction of two molecules of **13a** with residual Zr­(NEt_2_)_4_ and four additional equivalents of H_2_O, yielding the heterobimetallic polyoxocluster and Et_2_NH.

In contrast to the difficulties encountered with the heterometallic complexes, single crystals of **15** were readily obtained, allowing complete structural characterization. Compound **15** crystallized from a saturated toluene solution, and its molecular structure is shown in Figure 4.

**Figure 4:**
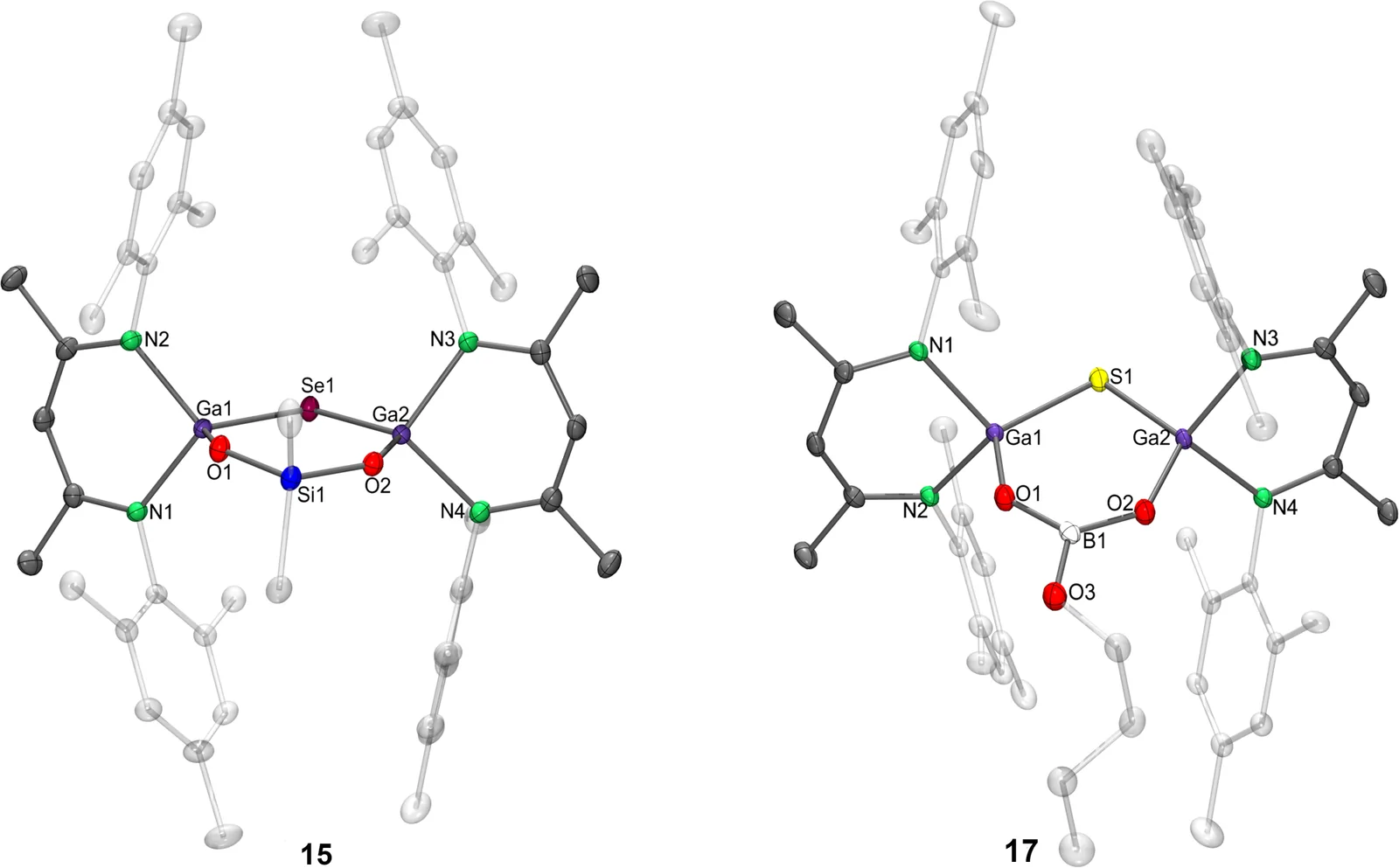
Molecular structures of **15** and **17** with thermal ellipsoids at 50% probability. The hydrogen atoms and solvent molecules were omitted for clarity.

### Reactivity of **4** and **5** with Boron Hydrides

In the study of the reactions of boron hydrides BH_3_·D (D = THF, SMe_2_) with galloxane dihydroxide **I** (Scheme 1a), two key reaction pathways were identified: protonolysis and hydride nucleophilic attack on the gallium centers.^26^ The cleavage of the GaO–H and the Ga–O bonds is consistent with these respective mechanistic processes.

Given the previously observed differences in reactivity, the ambiphilic character of the Ga–OH groups in compounds **4** and **5** was investigated through their reactions with BH_3_·THF. These reactions led to the formation of cyclic gallium borates of the type [{(^Me^LGa)_2_(μ-E)}­(μ-O)_2_{B­(O^*n*^Bu)}] [E = S (**17**), Se (**19**)] (Scheme 4), in which the O^*n*^Bu substituent originates from reductive cleavage of the THF molecule coordinated to BH_3_.^26^ Notably, conversely to compound **I** described earlier (Scheme 1a), excess BH_3_·THF does not affect the stability of **17** and **19**.

**Scheme 4:**
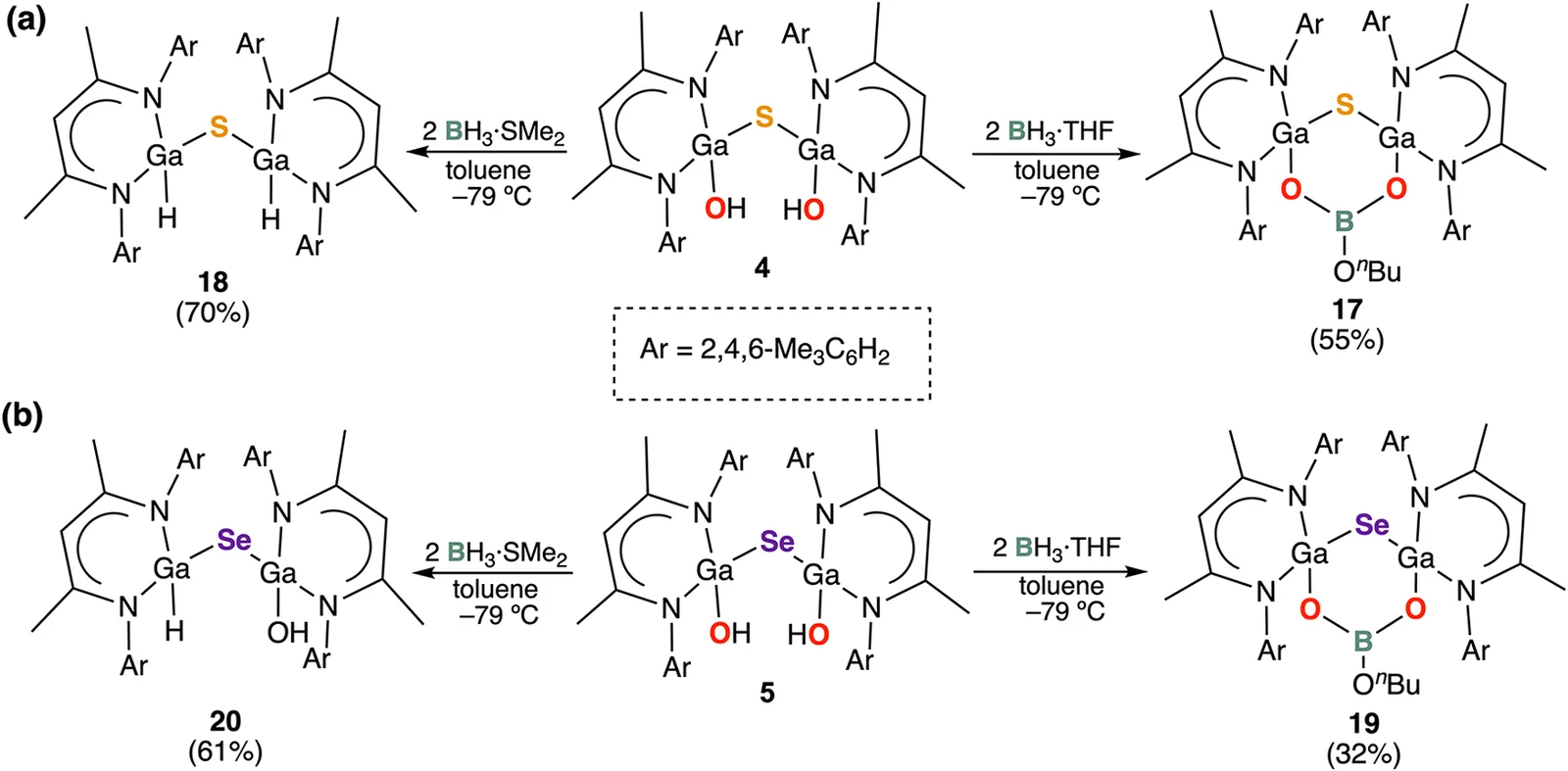
Reactivity of **4** and **5** with Boron Hydrides BH_3_·D (D = THF, SMe_2_)

In contrast, when BH_3_·SMe_2_ was reacted with **4**, direct transfer of two hydride groups to the gallium centers was observed, resulting in the formation of the digallium sulfide dihydride complex, [{^Me^LGa­(H)}_2_(μ-S)] (**18**) (Scheme 4a).

Compound **5**, on the other hand, reacted with BH_3_·SMe_2_ to yield the asymmetric digallium selenide hydride hydroxide complex [{^Me^LGa­(H)}­(μ-Se)­{^Me^LGa­(OH)}] (**20**), in which only one hydride was transferred to a gallium center (Scheme 4b). In addition, two minor side products were identified by ^1^H NMR spectroscopy: [{^Me^LGa­(H)}_2_(μ-Se)] (**20a**) and [{(^Me^LGa)_2_(μ-Se)}­(μ-O)_2_{BH}] (**20b**), each formed in less than 5% yield (SI).

It is noteworthy to mention that the cleavage of the Ga–O–Ga unit in **I** by the hydride nucleophilic attack of BH_3_ is also facilitated by the formation of a strong B–O bond (787 kJ mol^–1^).^26,48^

The ^1^H NMR spectra of compounds **17**–**19** exhibit characteristic splitting of the signals corresponding to the *o*-Ar methyl groups and the *m*-Ar hydrogen atoms of the ligand. As previously mentioned, this splitting results from decreased symmetry after the formation of the Ga–E–Ga bridge. For compounds **17** and **19**, signals are observed for the methyl (δ 1.03 ppm) and methylene (δ 1.55, 1.62, and 3.94 ppm for **17**; and δ 1.55, 1.62, and 3.97 ppm for **19**) of the O^*n*^Bu group.

The ^11^B NMR spectra of **17** and **19** display a broad single signal at δ 18.5 and 17.8 ppm, respectively. In comparison, the related galloxane borate [{(^Me^LGa)_2_(μ-O)}­(μ-O)_2_{B­(O^*n*^Bu)}] (**(** ^**Me**^ **LGa)**_**2**_ **O**_**3**_ **B**; Scheme 1a) shows the corresponding ^11^B NMR signal at δ 23.2 ppm.^26^ The EI-MS spectra of **17** and **19** reveal the characteristic molecular ion [*M*]^+^ for each compound at *m/z* 954 and 1002, respectively.

The ^1^H NMR spectrum of compound **18** displays a broad signal at δ 4.70 ppm corresponding to the hydride group. The IR spectrum shows a distinctive band at *ṽ* 1872 cm^–1^, attributed to the Ga–H stretching vibration. The EI-MS spectrum of **18** exhibits characteristic peaks at *m/z* 849 [M]^+^ and 838 [M – 2H]^+^.

In the case of compound **20**, its ^1^H NMR spectrum displays more signal splitting. First, the splitting of the *o*-Ar–Me and *m*-Ar–H signals from the β-diketiminate ligand due to the formation of a bridging Se motif. Second, each of these signals appear as a doublet because of the different functional groups attached to the gallium centers (H and OH). The Ga–H and Ga–OH signals appear at δ 5.22 and −0.98 ppm, respectively. The signals from the methine (CH) groups in the two ligand units appear at δ 4.68 and 4.58 ppm. Additionally, the EI-MS spectrum of compound **20** shows characteristic peaks at *m/z* 886 [M *–* H_2_O]^+^ and 902 [M – 2H]^+^.

Single crystals of compound **17** were obtained from a benzene solution at ambient temperature (Figure 4), whereas crystals of compounds **18** and **19** were grown from concentrated toluene solutions. Due to the low quality of the crystallographic data for **18** and **19**, only connectivity models were obtained for these compounds (SI).

Compound **17** features a boron atom in a planar trigonal geometry, and the six-membered GaSGaOBO ring adopts a planar configuration. The O–B–O angle in **17** [126.5(2)°] is only slightly larger than that observed in the borate analog **(** ^**Me**^ **LGa)**_**2**_ **O**_**3**_ **B**, [125.6(2)°] (Scheme 1a),^26^ which can be attributed to the different bridging μ-E atoms between the gallium centers.

## Conclusions

In summary, the digallium chalcogenide dihydroxides [{^Me^LGa­(OH)}_2_(μ-E)] [E = S (**4**), Se (**5**)] were synthesized through a metathesis–ammonolysis–hydrolysis sequence starting from ^Me^LGaCl_2_ (**1**). These compounds constitute the earliest examples of structurally characterized digallium chalcogenide frameworks featuring terminal Ga–OH groups. The reactivity patterns observed toward Group 4 metal amides, silicon amides, and boranes, identify μ-E substitution as a tool for modulating between protonolysis and hydride nucleophilic attack pathways. This study highlights that chalcogen bridging can be employed to modify the reactivity of gallium hydroxide systems.

## Experimental Section

### General Considerations

All experiments and characterizations were performed under an N_2_ atmosphere using Schlenk and glovebox techniques. All the chemical reagents and anhydrous ammonia (99.99%) were purchased from Sigma-Aldrich. Compound **1** was prepared according to the literature procedure.^23^ All solvents were dried with an MBraun SPS using Grubbs-type columns before use. C_6_D_6_ was dried with a Na/K alloy and distilled using a Swagelok system. NMR spectra were recorded at 25 °C on a Bruker Advance III 300 MHz spectrometer. The ^1^H and ^13^C NMR spectra were referenced to the residual non-deuterated and deuterated solvents, respectively, whereas the ^11^B and ^77^Se NMR spectra were referenced to Et_2_O·BF_3_ and (CH_3_)_2_Se, respectively. Melting points were measured in sealed glass tubes using a Büchi B-540 melting point apparatus. Mass spectra were performed on a Shimadzu GCMS-QP2010 Plus mass spectrometer using direct injection and electron impact (EI) as the ionization technique with a detection range from 20 to 1090 *m/z*. Infrared spectra were measured using attenuated total reflectance (ATR) on a Bruker Alpha FT-IR spectrometer under an inert atmosphere in the range *ṽ* 4000–400 cm^–1^. Elemental analyses (C, H, N, S) were performed on an Elementar MicroVARIO Cube analyzer. Crystallographic data and details can be found in the SI.

**Caution!** Sodium–potassium alloy (Na/K) is highly reactive, corrosive, and pyrophoric. It reacts violently with water and may ignite upon exposure to air or oxygen. Exposure to oxygen can produce potassium superoxide (KO_2_), a powerful oxidizer that may react violently with Na/K, organic materials, or water. Na/K must be handled under an inert atmosphere (argon or nitrogen) using appropriate protective equipment and established procedures.^49^

**Caution!** Ammonia gas is corrosive and toxic! Reactions in ammonia require a well-ventilated hood with the reaction vessel outlet leading to the hood exhaust. Ensure all health and safety protocols are observed when working with ammonia in gaseous or liquid form during experiments.^50^

### Synthesis of [{^Me^LGa­(Cl)}_2_(μ-S)] (**2**)

Na (0.048 g, 2.08 mmol) and S_8_ (0.033 g, 1.04 mmol) were placed in a Schlenk flask and introduced into a cold methanol bath at −50 °C. Then, 5 mL of NH_3_ (approx.) were condensed inside. Thus, the mixture was stirred until all the sodium metal and elemental sulfur were dissolved in NH_3_(*l*). After five minutes, under stirring and keeping the cold bath, ^Me^LGaCl_2_ (**1**) (1.0 g, 2.08 mmol in 40 mL of THF) was added dropwise. The reaction mixture was stirred for 20 min, and the cold bath was removed following safety precautions. Ammonia was evaporated while the reaction gradually reached ambient temperature under a dynamic nitrogen (N_2_) gas flux. The reaction mixture was stirred for 16 h and then filtered through Celite. All the volatiles were removed under vacuum. The isolated white solid was washed with hexane (3 × 3 mL). Yield 49% (0.45 g). Mp 269 °C. ^1^H NMR (300 MHz, CDCl_3_, 25 °C): δ 1.62 (s, 6H, C*H*_3_), 1.91, 2.31 (s, 12H, *o*–Ar–C*H*_3_), 2.15 (s, 6H, *p*–Ar–C*H*_3_), 4.92 (s, 1H, γ–C*H*), 6.64, 6.84 ppm (s, 4H, *m*-Ar-*H*). IR (ATR): ṽ 1609 (w), 1583 (sh), 1569 (m), 1552 (s), 1534 (m), 1503 (s), 1484 (w), 1470 (w), 1434 (w), 1379 (m), 1312 (m), 1294 (m), 1209 (w), 965 (w), 850 (m), 798 (sh), 667 (w), 543 (s), 442 (w), 421 (m) cm^–1^. EI-MS (70 eV) *m/z*: 908 [M – H]^+^. Elemental analysis (%) calcd. for C_46_H_58_Cl_2_Ga_2_N_4_S (909.40): C 60.75, H 6.43, N 6.16, S 3.53; found: C 60.46, H 6.65, N 6.21, S 3.46.

### Synthesis of [{^Me^LGa­(NH_2_)}_2_(μ-S)] (**3**)

A solution of **1** (1.0 g, 2.08 mmol) in 40 mL of THF was dropped into a mixture of Na (0.11 g, 4.6 mmol) and S_8_ (0.033 g, 1.04 mmol) solubilized in NH_3_(*l*) (5 mL, −50 °C) under stirring. After 20 min, the cold bath was removed, and ammonia evaporated under N_2_ flux. The reaction mixture was stirred for 16 h (ambient temperature), followed by filtration through Celite. All volatiles were removed under vacuum, and the light-green solid was washed with cold pentane (2 × 1 mL). Yield 41% (0.38 g). Mp 248 °C. ^1^H NMR (300 MHz, C_6_D_6_, 25 °C): δ −0.90 (s, 1H, N*H*_2_), 1.45 (s, 6H, C*H*_3_), 2.24, 2.34 (s, 12H, *o*–Ar–C*H*_3_), 2.31 (s, 6H, *p*–Ar–C*H*_3_), 4.65 (s, 1H, γ–C*H*), 6.70, 6.82 ppm (s, 4H, *m*-Ar-*H*). ^13^C NMR (75 MHz, C_6_D_6_, 25 °C): δ 18.9 (*C*H_3_), 19.7 (*p*-Ar-*C*H_3_), 20.9, 22.8 (*o*-Ar-*C*H_3_), 94.1 (γ-*C*H_3_), 129.1, 129.5 (*m*-Ar-*C*), 133.7, 134.6 (*o*-Ar-*C*), 134.7 (*p*-Ar-*C*), 141.5 (*i*-Ar-*C*), 167.6 ppm (*C*=N). IR (ATR): ṽ 3409 (m) and 3324 (m) cm^–1^. EI-MS (70 eV) *m/z*: 854 [M – NH_2_]^+^. Elemental analysis (%) calcd. for C_46_H_62_Ga_2_N_6_S (870.54): C 63.47, H 7.18, N 9.65, S 3.68; found: C 63.32, H 7.12, N 9.12, S 3.46.

### Synthesis of [{^Me^LGa­(OH)}_2_(μ-S)] (**4**)

Na (0.12 g, 5.21 mmol), and S_8_ (0.033 g, 1.04 mmol) in a Schlenk flask at −50 °C were dissolved in condensed NH_3_ (5 mL) and stirred until both reagents were solubilized. Compound **1** (1.0 g, 2.08 mmol in 40 mL THF) was added dropwise to the reaction mixture. After 20 min, ammonia evaporation was performed under N_2_ flux while the reaction reached ambient temperature. The mixture was stirred for 16 h. The reaction mixture was filtered through Celite, and the solution was cooled to −79 °C. A solution of H_2_O (0.5 M, 2.08 mmol) in THF was added dropwise. After 20 min of stirring, the cold bath was removed, and the reaction mixture was stirred overnight at ambient temperature. It was then filtered through Celite. All volatiles were removed, and the resulting white solid was washed with pentane (3 × 3 mL). Yield 43% (0.40 g). Mp 275 °C. ^1^H NMR (300 MHz, C_6_D_6_, 25 °C): δ −0.45 (s, 1H, O*H*), 1.43 (s, 6H, C*H*_3_), 2.23, 2.40 (s, 12H, *o*–Ar–C*H*_3_), 2.26 (s, 6H, *p*–Ar–C*H*_3_), 4.65 (s, 1H, γ–C*H*), 6.69, 6.85 ppm (s, 4H, *m*-Ar-*H*). ^1^H NMR (300 MHz, toluene-d_8_, 25 °C): δ −0.60 (s, 1H, O*H*), 1.42 (s, 6H, C*H*_3_), 2.15, 2.32 (s, 12H, *o*–Ar–C*H*_3_), 2.26 (s, 6H, *p*–Ar–C*H*_3_), 4.63 (s, 1H, γ–C*H*), 6.63, 6.80 ppm (s, 4H, *m*-Ar-*H*). ^13^C NMR (75 MHz, toluene-d_8_, 25 °C): δ 19.5 (*C*H_3_), 19.7 (p-Ar-*C*H_3_), 21.4, 23.0 (*o*-Ar-*C*H_3_), 94.8 (γ-*C*H_3_), 129.6, 129.9 (*m*-Ar-*C*), 134.5, 135.1 (*o*-Ar-*C*), 135.3 (*p*-Ar-*C*), 141.2 (*i*-Ar-*C*), 168.6 ppm (*C*=N). IR (ATR): ṽ 3616 (m) cm^–1^ (GaO–H). EI-MS (70 eV) *m/z*: 871 [M – H]^+^. Elemental analysis (%) calcd. for C_46_H_60_Ga_2_N_4_O_2_S (872.51): C 63.32, H 6.93, N 6.42, S 3.68; found: C 62.82, H 6.35, N 6.35, S 3.47.

### Synthesis of [{^Me^LGa­(OH)}_2_(μ-Se)] (**5**)

Compound **5** was synthesized using the same procedure outlined above for **4**, starting from Na (0.10 g, 4.50 mmol), Se (0.07 g, 0.90 mmol), **1** (0.85 g, 1.80 mmol), and 1.8 mL of the solution of H_2_O (0.5 M in THF, 0.90 mmol). Yield 42% (0.34 g). Mp 332 °C (dec). ^1^H NMR (300 MHz, C_6_D_6_, 25 °C): δ −0.31 (s, 1H, O*H*), 1.43 (s, 6H, C*H*_3_), 2.24, 2.41 (s, 12H, *o*–Ar–C*H*_3_), 2.27 (s, 6H, *p*–Ar–C*H*_3_), 4.65 (s, 1H, γ–C*H*), 6.71, 6.86 ppm (s, 4H, *m*-Ar-*H*). ^1^H NMR (300 MHz, CDCl_3_, 25 °C): δ −0.52 (s, 1H, O*H*), 1.57 (s, 6H, C*H*_3_), 1.94, 2.31 (s, 12H, *o*–Ar–C*H*_3_), 2.14 (s, 6H, *p*–Ar–C*H*_3_), 4.82 (s, 1H, γ–C*H*), 6.67, 6.87 ppm (s, 4H, *m*-Ar-*H*). ^13^C NMR (75 MHz, C_6_D_6_, 25 °C): δ 19.2 (*C*H_3_), 19.8 (*p*-Ar-*C*H_3_), 21.1, 22.8 (*o*-Ar-*C*H_3_), 94.6 (γ*-C*H_3_), 129.5, 129.6 (*m*-Ar-*C*), 133.9, 134.7 (*o*-Ar-*C*), 135.0 (*p*-Ar-*C*), 140.7 (*i*-Ar-*C*), 168.4 ppm (*C*=N). ^77^Se NMR (C_6_D_6_, 25 °C): δ −555.3 ppm. IR (ATR): ṽ 3614 (s) (GaO–H), 3388 (s, b) (GaO–H, H-bonded) cm^–1^. EI-MS (70 eV) *m/z*: 902 [M – OH]^+^. Elemental analysis (%) calcd. for C_46_H_60_Ga_2_N_4_O_2_Se (919.42): C 60.09, H 6.58, N 6.09; found: C 58.03, H 5.91, N 5.86.

### Synthesis of [{^Me^LGa­(NH_2_)}_2_(μ-O)] (**6**)

Compound **6** was isolated by attempts to isolate [{LGa­(NH_2_)}_2_(μ-Te)]. Na (0.07 g, 2.91 mmol) and Te (0.09 g, 0.73 mmol) were placed to react in NH_3_ (*l*) (5 mL) at −50 °C. After both reagents were solubilized and stirred for 20 min, compound **1** (0.69 g, 1.47 mmol in 40 mL of THF) was added dropwise at −50 °C. The cold bath was removed after 20 min. Ammonia was evaporated under a dynamic N_2_ flux, and the reaction mixture was stirred for 16 h at ambient temperature. The reaction mixture was filtered, and after 16 h, single crystals of **6** were obtained from this solution in low yields. Yield 6% (0.035 g). Mp 176 °C. ^1^H NMR (300 MHz, C_6_D_6_, 25 °C): δ −1.13 (s, 1H, N*H*_2_), 1.45 (s, 6H, C*H*_3_), 2.25, 2.32 (s, 12H, *o*–Ar–C*H*_3_), 2.25 (s, 6H, *p*–Ar–C*H*_3_), 4.68 (s, 1H, γ–C*H*), 6.78, 6.86 ppm (s, 4H, *m*-Ar-*H*). IR (ATR): ṽ 3409 (w) and 3325 (w) cm^–1^. EI-MS (70 eV) *m/z*: 838 [M – NH_2_]^+^. No elemental analysis data was obtained because of the extremely sensitive nature of this compound.

### Synthesis of [{(^Me^LGa)_2_(μ-S)}­(μ-O)_2_{Ti­(NMe_2_)_2_}] (**9**)

Ti­(NMe_2_)_4_ (0.046 g, 0.20 mmol) in toluene (10 mL) was placed in a cold bath at −79 °C. A solution of compound **4** (0.14 g, 0.16 mmol) in toluene (10 mL) was transferred via cannula to the titanium amide complex solution. The cold bath was then removed, and the mixture was stirred overnight at ambient temperature. The solution was filtered, and all volatiles were removed under vacuum. The residual yellow solid was washed with cold pentane (3 × 3 mL). Yield 68% (0.11 g). Mp 331 °C. ^1^H NMR (300 MHz, C_6_D_6_, 25 °C): δ 1.39 (s, 6H, C*H*_3_), 2.18, 2.36 (s, 12H, *o*–Ar–C*H*_3_), 2.32 (s, 6H, *p*–Ar–C*H*_3_), 2.99 [s, 6H, N­(C*H*_3_)_2_], 4.75 (s, 1H, γ–C*H*), 6.79, 6.81 ppm (s, 4H, *m*-Ar-*H*). ^13^C NMR (75 MHz, C_6_D_6_, 25 °C): δ 19.3 (*C*H_3_), 21.2 (*p*-Ar-*C*H_3_), 19.4, 23.0 (*o*-Ar-*C*H_3_), 45.8 [N­(*C*H_3_)_2_], 96.3 (γ*-C*H_3_), 129.5, 129.8 (*m*-Ar-*C*), 133.8, 134.5 (*o*-Ar-*C*), 134.4 (*p*-Ar-*C*), 141.7 (*i*-Ar-*C*), 168.7 ppm (*C*=N). IR (ATR): ṽ 2963 (w), 2914 (w), 2851 (w), 2811 (m), 2761 (m), 1526 (s), 1477 (w), 1453 (w), 1288 (s), 1226 (m), 1202 (w), 1160 (m), 1148 (w), 1120 (w), 1022 (m), 865 (s), 774 (s), 729 (m), 694 (m), 582 (sh), 567 (w), 503 (w), 460 (m), 407 (m) cm^–1^. EI-MS (70 eV) *m/z*: 1006 [*M*]^+^. Elemental analysis (%) calcd. for C_50_H_70_Ga_2_N_6_O_2_STi (1006.52): C 59.67, H 7.01, N 8.35, S 3.19; found: C 58.39, H 6.67, N 7.50, S 2.68. The difference between the observed and calculated EA values could be due to the highly air-sensitive nature of the compound.

### Synthesis of [{(^Me^LGa)_2_(μ-S)}­(μ-O)_2_{Zr­(NEt_2_)_2_}] (**10**)

Compound **10** was prepared following the same experimental procedure used for **9**, starting from 0.15 g of compound **4** (0.17 mmol) and 0.073 g of Zr­(NEt_2_)_4_ (0.19 mmol). Yield 41% (0.09 g). Mp 267 °C. ^1^H NMR (300 MHz, C_6_D_6_, 25 °C): δ 1.23 [t, ^3^ *J* = 7.2 Hz, 6H, N­(CH_2_C*H*_3_)_2_], 1.40 (s, 6H, C*H*_3_), 2.20, 2.36 (s, 12H, *o*–Ar–C*H*_3_), 2.33 (s, 6H, *p*–Ar–C*H*_3_), 3.10 [q, ^3^ *J* = 7.2 Hz, 4H, N­(C*H*_2_CH_3_)_2_], 4.75 (s, 1H, γ–C*H*), 6.80, 6.85 ppm (s, 4H, *m*-Ar-*H*). The ^13^C NMR spectrum could not be obtained because of significant sample precipitation after preparation. IR (ATR): ṽ 2955 (w), 2917 (w), 2857 (w), 2813 (w), 2737 (w), 1533 (sh), 1454 (m), 1387 (s), 1291 (w), 1248 (w), 1206 (w), 1183 (w), 1149 (w), 1124 (w), 1023 (m), 864 (m), 843 (w), 788 (s), 728 (m), 698 (m), 652 (w), 569 (m), 547 (w), 491 (w), 463 (m), 396 (m) cm^–1^. The EI-MS spectrum only showed characteristic fragment ions corresponding to the gallium chalcogenide ligand. No elemental analysis data were obtained because of the extremely sensitive nature of **10**.

### Synthesis of [{(^Me^LGa)_2_(μ-S)}­(μ-O)_2_{Hf­(NEt_2_)_2_}] (**11**)

Compound **11** was prepared following the same experimental procedure used for **9**, starting from 0.10 g of **4** (0.11 mmol) and 0.048 g of Hf­(NEt_2_)_4_ (0.13 mmol). Yield 53% (0.08g). Mp 270 °C. ^1^H NMR (300 MHz, C_6_D_6_, 25 °C): δ 1.24 [t, ^3^ *J* = 7.2 Hz, 6H, N­(CH_2_C*H*_3_)_2_], 1.40 (s, 6H, C*H*_3_), 2.20, 2.36 (s, 12H, *o*–Ar–C*H*_3_), 2.32 (s, 6H, *p*–Ar–C*H*_3_), 3.11 [q, ^3^ *J* = 7.2 Hz, 4H, N­(C*H*_2_CH_3_)_2_], 4.75 (s, 1H, γ–C*H*), 6.80, 6.84 ppm (s, 4H, *m*-Ar-*H*). The ^13^C NMR spectrum was not obtained because of concentrated sample precipitation after preparation. IR (ATR): ṽ 2915 (m), 1608 (m), 1528 (w), 1452 (w), 1385 (s), 1288 (w), 1254 (m), 1227 (w), 1201 (w), 1148 (w), 1095 (w), 1020 (w), 961 (w), 861 (m), 836 (m), 759 (m), 729 (w), 651 (w), 634 (w), 569 (w), 504 (w), 420 (m) cm^–1^. The EI-MS spectrum only showed characteristic fragment ions corresponding to the gallium chalcogenide ligand. No elemental analysis data were obtained for this compound because of its extremely sensitive nature.

### Synthesis of [{(^Me^LGa)_2_(μ-S)}­(μ-O)_2_]_2_Ti (**12**)

Ti­(NMe_2_)_4_ (0.04 g, 0.20 mmol) in toluene (10 mL) was added dropwise to a solution of **4** (0.14 g, 0.16 mmol, 10 mL of toluene) at −79 °C via cannula. The reaction mixture was stirred overnight at ambient temperature and filtered through a Celite filter. All the volatiles were removed under vacuum. The residual white solid was crystallized from pentane. Yield 42% (0.06 g). ^1^H NMR (300 MHz, C_6_D_6_, 25 °C): δ 1.53 (s, 6H, C*H*_3_), 2.11 (s, 6H, *p*–Ar–C*H*_3_), 2.47, 2.53 (s, 12H, *o*–Ar–C*H*_3_), 4.93 (s, 1H, γ–C*H*), 6.84, 7.03 ppm (s, 4H, *m*-Ar-*H*). ^13^C NMR spectrum acquisition was not possible because of the concentrated sample precipitation after preparation. IR (ATR): ṽ 2999 (m), 2914 (m), 2855 (m), 1610 (w), 1553 (w), 1531 (m), 1453 (w), 1388 (sh), 1287 (w), 1251 (w), 1226 (w), 1202 (m), 1149 (w), 1020 (m), 861 (m), 842 (w), 772 (s), 746 (s), 726 (s), 651 (w), 633 (w), 603 (w), 582 (w), 569 (w), 539 (w), 504 (m), 480 (m), 407 (w) cm^–1^. The EI-MS spectrum only showed characteristic fragment ions corresponding to the gallium chalcogenide ligand. Elemental analysis (%) calcd. for C_92_H_116_Ga_4_N_8_S_2_Ti (1788.87): C 61.77, H 6.54, N 6.26, S 3.58; found: C 61.10, H 6.50, N 6.19, S 3.38.

### Synthesis of [{(^Me^LGa)_2_(μ-S)}­(μ-O)_2_]_2_Zr (**13**)

Compound **13** was prepared following the same experimental procedure as that used for compound **12**, starting from **4** (0.14 g, 0.16 mmol) and Zr­(NEt_2_)_4_ (0.03 g, 0.08 mmol). Yield 42% (0.062 g). Mp 270 °C. ^1^H NMR (300 MHz, C_6_D_6_, 25 °C): δ 1.51 (s, 6H, C*H*_3_), 2.12 (s, 6H, *p*–Ar–C*H*_3_), 2.50, 2.55 (s, 12H, *o*–Ar–C*H*_3_), 4.90 (s, 1H, γ–C*H*), 6.84, 7.06 ppm (s, 4H, *m*-Ar-*H*). The ^13^C NMR spectrum was not obtained because the concentrated sample precipitated after preparation. IR (ATR): ṽ 2915 (w), 2850, (w), 2807 (w), 2758 (w), 1533 (s), 1457 (m), 1434 (m), 1389 (s), 1289 (w), 1250 (m), 1206 (m), 1150 (m), 1099 (w), 1020 (m), 945 (m), 864 (m), 843 (m), 790 (s), 717 (m), 652 (w), 633 (w), 605 (w), 568 (w), 490 (m), 450 (m), 398 (m) cm^–1^. The EI-MS spectrum only showed characteristic fragment ions corresponding to the gallium chalcogenide ligand. Elemental analysis (%) calcd. for **13** C_92_H_116_Ga_4_N_8_O_4_S_2_Zr (1832.21): C 60.31, H 6.38, N 6.12, S 3.50; found: C 61.98, H 7.10, N 6.28, S 3.58.

### Synthesis of [{(^Me^LGa)_2_(μ-S)}­(μ-O)_2_]_2_Hf (**14**)

Compound **14** was prepared using the same experimental procedure as that for compound **12**, starting from **4** (0.14 g, 0.16 mmol) and Hf­(NEt_2_)_4_ (0.069 g, 0.14 mmol). Yield 45% (0.07 g). ^1^H NMR (300 MHz, C_6_D_6_, 25 °C): δ 1.52 (s, 6H, C*H*_3_), 2.11 (s, 6H, *p*–Ar–C*H*_3_), 2.48, 2.53 (s, 12H, *o*–Ar–C*H*_3_), 4.92 (s, 1H, γ–C*H*), 6.84, 7.03 ppm (s, 4H, *m*–Ar-H). The ^13^C NMR spectrum was not obtained because of concentrated sample precipitation after preparation. IR (ATR): ṽ 2958 (w), 2918 (w), 1533 (s), 1454 (sh), 1387 (s), 1251 (w), 1204 (w), 1150 (w), 1125 (w), 1020 (m), 866 (w), 843 (w), 786 (s), 710 (w), 571 (m), 490 (w), 449 (m), 396 (m) cm^–1^. The EI-MS spectrum only showed characteristic fragment ions corresponding to the gallium chalcogenide ligand. No elemental analysis data were obtained for this compound because of its extremely sensitive nature.

### Synthesis of [{(^Me^LGa)_2_(μ-Se)}­(μ-O)_2_{SiMe_2_}] (**15**)

An excess of Me_2_Si­(NMe_2_)_2_ (0.05 mL, 0.27 mmol) was added dropwise to **5** (0.1 g, 0.10 mmol) in 15 mL of toluene at −79 °C. After the addition, the cold bath was removed. The reaction mixture was then stirred at ambient temperature for 16 h. The reaction mixture was filtered through Celite, and all volatiles were removed under vacuum. The white solid obtained was washed with hexane (3 × 3 mL). Yield 38% (0.04 g). Mp 384 °C. ^1^H NMR (300 MHz, C_6_D_6_, 25 °C): δ −0.07 (s, 6H, Si­(C*H*_3_)_2_), 1.41 (s, 6H, C*H*_3_), 2.23, 2.36 (s, 12H, *o*–Ar–C*H*_3_), 2.29 (s, 6H, *p*–Ar–C*H*_3_), 4.72 (s, 1H, γ–C*H*), 6.81, 6.86 ppm (s, 4H, *m*-Ar-*H*). IR (ATR): ṽ 3002 (w), 2952 (m), 2914 (m), 2854­(w), 1547 (m), 1528 (s), 1451 (m), 1378 (s), 1242 (m), 1202 (m), 1148 (m), 1019 (m), 957 (s), 863 (s), 841 (w), 808 (m), 762 (s), 636 (m), 568 (s), 504 (s), 489 (s) cm^–1^. EI-MS (70 eV) *m/z*: 976 [M]^+^. Elemental analysis (%) calcd. for C_48_H_64_Ga_2_N_4_O_2_SeSi (975.56): C 59.10, H 6.61, N 5.74; found: C 58.97, H 6.60, N 5.76.

### Compound **16**

Single crystals of polyoxocluster **16** were obtained from a concentrated toluene solution of **13**. Mp 284 °C. ^1^H NMR (300 MHz, C_6_D_6_, 25 °C): δ 1.50 (s, 6H, C*H*_3_), 2.10, 2.54 (s, 12H, *o*–Ar–C*H*_3_), 2.48 (s, 6H, *p*–Ar–C*H*_3_), 4.88 (s, 1H, γ–C*H*), 6.83, 7.05 ppm (s, 4H, *m*-Ar-*H*). The EI-MS spectrum only showed characteristic fragment ions corresponding to the gallium chalcogenide ligand. Elemental analysis (%) calcd. for **16** C_204_H_288_Ga_8_N_16_O_18_S_4_Zr_5_ (4394.59): C 55.75, H 6.61, N 5.15, S 2.92; found: C 55.94, H 6.25, N 5.29, S 2.53.

### Synthesis of [{(^Me^LGa)_2_(μ-S)}­(μ-O)_2_{B­(O^*n*^Bu)}] (**17**)

A solution of the BH_3_·THF complex (1.0 M in THF, 0.45 mL, 0.22 mmol) was added to **4** (0.10 g, 0.11 mmol) in toluene (15 mL) at −79 °C and stirred for 16 h at ambient temperature, filtered, and all the volatile material was removed under vacuum. The white residue was washed with hexane (3 × 3 mL). Yield 55% (0.06 g). Mp 311 °C. ^1^H NMR (300 MHz, C_6_D_6_, 25 °C): δ 1.03 [t, ^3^ *J =* 7.2 Hz, 3H, BO­(CH_2_)_3_C*H*_3_], 1.37 (s, 6H, C*H*_3_), 1.55 [m, ^3^ *J =* not observable, 2H, BO­(CH_2_)_2_C*H*_2_CH_3_], 1.62 (q, ^3^ *J =* not observable, 2H, BOCH_2_C*H*_2_CH_2_CH_3_), 2.13, 2.34 (s, 12H, *o*–Ar–C*H*_3_), 2.27 (s, 6H, *p*–Ar–C*H*_3_), 3.94 [t, ^3^ *J =* 6.6 Hz, 2H, BOC*H*_2_(CH_2_)_2_CH_3_], 4.71 (s, 1H, γ–C*H*), 6.76, 6.85 ppm (s, 4H, *m*-Ar–*H*). ^13^C NMR (75 MHz, C_6_D_6_, 25 °C): δ 14.4 [BO­(CH_2_)_3_ *C*H_3_], 18.5 (*C*H_3_), 19.0, 21.1 (*o*-Ar-*C*H_3_), 20.3 (*p*-Ar-*C*H_3_), 22.8 [BO­(CH_2_)_2_ *C*H_2_CH_3_], 35.1 (BOCH_2_ *C*H_2_CH_2_CH_3_), 62.8 [BO*C*H_2_(CH_2_)_2_CH_3_], 96.0 (γ-*C*H), 129.8, 130.2 (*m*-Ar-*C*), 133.4, 134.2 (*o*-Ar-*C*), 133.8 (*p*-Ar-*C*), 141.1 (*i*-Ar-*C*), 168.8 ppm (*C*=N). ^11^B NMR (C_6_D_6_, 25 °C): δ 18.5 ppm. IR (ATR): ṽ 3001 (w), 2950 (m), 2914 (m), 2861­(w), 2730 (w), 1610 (w), 1531 (s), 1451 (m), 1373 (s), 1336 (s), 1306 (m), 1288 (w),1220 (s), 1204 (m), 1104 (w), 961 (s), 935 (s), 865 (s), 841 (m), 762 (s), 684 (m),652 (w), 637 (w), 583 (s), 568 (w), 507 (s), 439 (m) cm^–1^. EI-MS (70 eV) *m/z*: 954 [*M*]^+^. No elemental analysis data were obtained for this compound because of its extremely sensitive nature.

### Synthesis of [{^Me^LGa­(H)}_2_(μ-S)] (**18**)

0.25 g of compound **4** (0.28 mmol) was dissolved in 20 mL of toluene. BH_3_·SMe_2_ (2.0 M in THF, 0.28 mL, 0.56 mmol) was added via cannula to a solution of **4** at −79 °C. The cold bath was removed after 20 min. The reaction mixture was stirred for 16 h at ambient temperature. All volatiles were removed under vacuum, and the yellow solid was washed with cold pentane (3 × 3 mL). Yield 70% (0.17 g). Mp 266 °C. ^1^H NMR (300 MHz, C_6_D_6_, 25 °C): δ 1.44 (s, 6H, C*H*_3_), 2.15, 2.30 (s, 12H, *o*–Ar–C*H*_3_), 2.26 (s, 6H, *p*–Ar–C*H*_3_), 4.62 (s, 1H, γ–C*H*), 4.70 (s, 1H, Ga–*H*), 6.68, 6.81 ppm (s, 4H, *m*-Ar-*H*). ^13^C NMR (75 MHz, CDCl_3_, 25 °C): δ 18.5 (*C*H_3_), 19.5 (*p*-Ar-*C*H_3_), 20.9, 22.5 (*o*-Ar-*C*H_3_), 94.0 (γ*-C*H_3_), 129.4, 129.6 (*m*-Ar-*C*), 132.4, 134.6 (*o*-Ar-*C*), 134.3 (*p*-Ar-*C*), 141.5 (*i*-Ar-*C*), 167.1 ppm (*C*=N). IR (ATR): ṽ 1872 (broad) cm^–1^ (Ga–H). EI-MS (70 eV) *m/z*: 838 [M – 2H]^+^. Elemental analysis (%) calcd. for C_46_H_60_Ga_2_N_4_S (840.52): C 65.73, H 7.20, N 6.67, S 3.81; found: C 64.24, H 7.37, N 6.31, S 3.70.

### Synthesis of [{(^Me^LGa)_2_(μ-Se)}­(μ-O)_2_{B­(O^*n*^Bu)}] (**19**)

Compound **19** was obtained following the same experimental procedure as that used for **17** starting from 0.1 g of **5** (0.10 mmol in 15 mL of toluene) and 0.22 mL of the BH_3_·THF solution (1.0 M in THF, 0.22 mmol). Yield 32% (0.035 g). Mp 309 °C. ^1^H NMR (300 MHz, C_6_D_6_, 25 °C): δ 1.03 [t, ^3^ *J =* 7.2 Hz, 3H, BO­(CH_2_)_3_C*H*_3_], 1.38 (s, 6H, C*H*_3_), 1.55 [m, ^3^ *J =* not observable, 2H, BO­(CH_2_)_2_C*H*_2_CH_3_], 1.62 (q, ^3^ *J =* not observable, 2H, BOCH_2_C*H*_2_CH_2_CH_3_), 2.13, 2.35 (s, 12H, *o*–Ar–C*H*_3_), 2.28 (s, 6H, *p*–Ar–C*H*_3_), 3.97 [t, ^3^ *J =* 6.3 Hz, 2H, BOC*H*_2_(CH_2_)_2_CH_3_], 4.71 (s, 1H, γ–C*H*), 6.80, 6.86 ppm (s, 4H, *m*-Ar-*H*). ^11^B NMR (C_6_D_6_, 25 °C): δ 17.8 ppm. IR (ATR): ṽ 3001 (w), 2951 (m), 2915 (m), 2855­(w), 2730 (w), 1609 (w), 1531 (s), 1452 (m), 1375 (s), 1248 (s), 1220 (m), 1203 (s), 1148 (m), 1103 (w), 1021 (s), 864 (s), 842 (m), 761 (s), 684 (m),652 (w), 637 (w), 582 (s), 506 (s), 438 (s) cm^–1^. EI-MS (70 eV) *m/z*: 1002 [*M*]^+^. Elemental analysis (%) calcd. for C_50_H_67_BGa_2_N_4_O_3_Se (1001.33): C 59.98, H 6.74, N 5.60; found: C 60.81, H 6.76, N 5.47.

### Synthesis of [{^Me^LGa­(H)}­(μ-Se)­{LGa­(OH)}] (**20**)

Compound **5** (0.10 g, 0.10 mmol) was dissolved in toluene (20 mL) and placed in a cold bath at −79 °C. BH_3_·SMe_2_ (2.0 M in THF, 0.11 mL, 0.22 mmol) was then added dropwise. The cold bath was removed after 20 min. The reaction mixture was then stirred at ambient temperature for 16 h. It was then filtered, and all volatiles were removed under vacuum. The resulting white solid was washed with pentane (3 × 3 mL). Yield 61% (0.06 g). ^1^H NMR (300 MHz, C_6_D_6_, 25 °C): δ −0.98 (s, 1H, O*H*), 1.40, 1.47 (s, 12H, C*H*_3_), 2.13, 2.27, 2.29, 2.47 (s, 24H, *o*–Ar–C*H*_3_), 2.27 (s, 12H, *p*–Ar–C*H*_3_), 4.58, 4.68 (s, 2H, γ–C*H*), 5.22 (s, 1H, Ga–*H*), 6.67, 6.75, 6.81, 6.86 ppm (s, 8H, *m*-Ar-*H*). IR (ATR): ṽ 3677 (broad) cm^–1^ (GaO–H). EI-MS (70 eV) *m/z*: 886 [M – H_2_O]^+^, 902 [M – 2H]^+^. No elemental analysis data were obtained for this compound because of its extremely moisture-sensitive nature.

## Supplementary Material



## Notes

^1^H, ^11^B, and ^13^C NMR, EI-MS, and IR spectra; general X-ray diffraction details; molecular structures of compounds **7** and **16**; connectivity model for **13**, **13a**, **18** and **19**; crystallographic refinement details and selected geometric parameters for **2**–**7**, **9**, **12**, **15**–**17**, and **19**; DFT calculations: Non-covalent interaction (NCI) for complexes **I**, **4**, and **5** (PDF)
