# Supplementary material for: Tailoring Reactivity of Molecular Gallium Hydroxides via Chalcogen Bridging
Source: Inorg Chem. 2026 Sep 14;65(41):24295–307. doi: 10.1021/acs.inorgchem.6c03433 (PMC13685416; doi:10.1021/acs.inorgchem.6c03433)
Supplement: Supplementary file 1 [file ic6c03433_si_001.pdf]

Supporting Information

for

Tailoring Reactivity of Molecular Gallium Hydroxides via Chalcogen Bridging

Erandi Bernabé-Pablo,[a,b]* *Alfonso Gallardo-Garibay,*[a,b] *Vojtech Jancik,*[a,b] *Diego Martínez-Otero,*[a,b] *Roman Neufeld,*[c] *Uvaldo Hernández-Balderas,*[a,b] *Dietmar Stalke,*[c] *and Mónica Moya-Cabrera*[a,b]*

[a]Universidad Nacional Autónoma de México, Instituto de Química, Ciudad Universitaria, Ciudad de México, 04510, México

[b]Centro Conjunto de Investigación en Química Sustentable UAEM-UNAM, Carretera Toluca-Atlacomulco Km 14.5, Toluca, Estado de México, 50200, México

[c]Institut für Anorganische Chemie, Georg-August Universität Göttingen, Tammannstrasse 4, 37077, Göttingen, Germany

***Corresponding Author:**

* E-mail: monica.moya@unam.mx. Phone: +52 (722) 276 66 10, ext. 7726. Fax: (+52) 55 56 16 22 17. * E-mail: erandi.bernabe@iquimica.unam.mx. Phone: +52 (722) 276 66 10, ext. 7771. Fax: (+52) 55 56 16 22 17.

Table of Contents

1. General X-ray diffraction details **S4**

2. SCHEMES, FIGURES AND TABLES **S4**

Scheme S1. Proposed reaction pathways for the formation of the ammonia-solvated species **3**·$(NH_3)_n$ from the ammonia-solvated species of **2**·$(NH_3)_{n-2}$ **S4**

Figure S1. ^{1}H NMR spectrum of **2** in $CDCl_3$ **S5**

Figure S2. EI-MS spectrum of **2** **S5**

Figure S3. ^{1}H NMR spectrum of **3** in C_6D_6 **S6**

Figure S4. ^{13}C NMR spectrum of **3** in C_6D_6 **S6**

Figure S5. IR(ATR) spectrum of **3** **S7**

Figure S6. EI-MS spectrum of **3** **S7**

Figure S7. ^{1}H NMR spectrum of **4** in C_6D_6 **S8**

Figure S8. ^{13}C NMR spectrum of **4** in toluene-d_8 **S8**

Figure S9. IR(ATR) spectrum of **4** **S9**

Figure S10. ^{1}H NMR spectrum of **5** in C_6D_6 **S9**

Figure S11. ^{13}C NMR spectrum of **5** in C_6D_6 **S10**

Figure S12. IR(ATR) spectrum of **5** **S10**

Figure S13. EI-MS spectrum of **5** **S11**

Figure S14. ^{1}H NMR spectrum of **6** in C_6D_6 **S11**

Figure S15. IR(ATR) spectrum of **6** **S12**

Figure S16. EI-MS spectrum of **6** **S12**

Figure S17. ^{1}H NMR spectrum of **9** in C_6D_6 **S13**

Figure S18. ^{13}C NMR spectrum of **9** in C_6D_6 **S13**

Figure S19. EI-MS spectrum of **9** **S14**

Figure S20. ^{1}H NMR spectrum of **10** in C_6D_6 **S14**

Figure S21. ^{1}H NMR spectrum of **11** in C_6D_6 **S15**

Figure S22. ^{1}H NMR spectrum of **12** in C_6D_6 **S15**

Figure S23. ^{1}H NMR spectrum of **13** in C_6D_6 **S16**

Figure S24. ^{1}H NMR spectrum of **14** in C_6D_6 **S16**

Figure S25. ^{1}H NMR spectrum of **15** in C_6D_6 **S17**

Figure S26. EI-MS spectrum of **15** **S17**

Figure S27. ^{1}H NMR spectrum of **17** in C_6D_6 **S18**

Figure S28. ^{13}C NMR spectrum of **17** in C_6D_6 **S18**

Figure S29. ^{11}B NMR spectrum of **17** in C_6D_6 **S19**
Figure S30. EI-MS spectrum of **17** **S19**
Figure S31. ^{1}H NMR spectrum of **18** in C_6D_6 **S20**
Figure S32. ^{13}C NMR spectrum of **18** in C_6D_6 **S20**
Figure S33. EI-MS spectrum of **18** **S21**
Figure S34. IR (ATR) spectrum of **18** **S21**
Figure S35. ^{1}H NMR spectrum of **19** in C_6D_6 **S22**
Figure S36. ^{11}B NMR spectrum of **19** in C_6D_6 **S22**
Figure S37. EI-MS spectrum of **19** **S23**
Figure S38. ^{1}H NMR spectrum of **20** in C_6D_6 **S23**
Figure S39. EI-MS spectrum of **20** **S24**
Figure S40. Molecular structure of **7** **S24**
Figure S41. Molecular structures of **8** and **8a** **S25**
Table S1. Crystallographic Data and Refinement Details for Compounds **2**–**7**, **8** and **8a** **S26**
Table S2. Selected Bond Lengths (Å) and Angles (deg) for Compounds **2** and **6** **S27**
Table S3. Selected Bond Lengths (Å) and Angles (deg) for Compounds **3**–**5** **S27**
Table S4. Crystallographic Data and Refinement Details for **9**, **12**, **15**, **16** and **17** **S28**
Table S5. Selected Bond Lengths (Å) and Angles (deg) for Compounds **9**, **12**, **15** and **17** **S29**
Figure S42. Ball-and-stick diagram of connectivity model for **13**, **13a**, **18** and **19** **S30**
Figure S43. Molecular structure of **16** **S31**
Table S6. Selected bond lengths (Å) and angles (deg) for **16** **S32**
3. COMPUTATIONAL DETAILS **S33**
Non-Covalent Interactions (NCI) analysis for hydrogen bond formation **S33**
Figure S44. NCI surfaces for **I**, **4**, and **5** and overlap of s(r) vs sign(λ_2)ρ plots **S34**
4. REFERENCES **S35**

1. General X-ray diffraction details

Crystallographic data for **3** and **7** were collected on a Bruker Smart Apex II Quazar diffractometer with an INCOATEC micro source and a mirror-monochromated MoKα radiation ($\lambda = 0.71073$ Å). The X-ray data sets for **2**–**6**, **8**, **9**, **12**, and **15**–**17** were collected on a Bruker SMART APEX DUO three-circle diffractometer equipped with an Apex II CCD detector using MoK_{α} (Inotec sealed tube with a graphite monochromator). The crystals were coated with hydrocarbon oil and picked up with a nylon loop to mount them directly in the cold nitrogen stream (−173 °C) of the diffractometer. Frames were collected by omega scans. Data integration was performed using the SAINT program. A semi-empirical absorption correction was applied using SADABS.[1] Direct methods (SHELXS) were used to solve the structures. The refinements by the full-matrix least-squares on F^2 were performed with SHELXL-97[2] using the SHELXLE GUI.[3] Weighted R factors, R_w, and all goodness-of-fit indicators were based on F^2. The non-hydrogen atoms were refined anisotropically. The hydrogen atoms were placed in idealized geometrical positions and refined with U_{iso} tied to the parent atom using the riding model. Hydrogen atoms of the OH and NH_2 moieties in **3**–**6** were localized from the difference electron-density map and refined isotropically with U_{ij} tied to the nitrogen or oxygen atom, respectively.

2. SCHEMES, FIGURES AND TABLES

The ammonolysis reaction of Ga−Cl bonds in ammoniated digallium sulfide dichloride **2** yields **3** and NH_4Cl, as detailed in Eq. 1 of **Scheme S1**. Then, sodium electride and ammonium chloride react, producing sodium chloride, ammonia, and hydrogen (Eq. 2 of **Scheme S1**). This mechanism resembles the one reported for aluminum amide synthesis using AlI_3 and alkali metals in liquid ammonia.[4]

$$\underset{\mathbf{2}\cdot(NH_3)_n}{[\{^{Me}LGa(Cl)\}_2(\mu\text{-}S)](NH_3)_n} \xrightarrow{\textit{Ammonolysis}} \underset{\mathbf{3}\cdot(NH_3)_{n-2}}{[\{^{Me}LGa(NH_2)\}_2(\mu\text{-}S)](NH_3)_{n-2}} + 2\ NH_4Cl \quad [1]$$

$$2\ NH_4Cl + 2\ [Na(NH_3)_6]^+e^- \longrightarrow 2\ NaCl + 2\ NH_3 + H_2\uparrow \quad [2]$$

Scheme S1. Proposed reaction pathways for the formation of the ammonia-solvated species $\mathbf{3}\cdot(NH_3)_n$ from the ammonia-solvated species of $\mathbf{2}\cdot(NH_3)_{n-2}$.

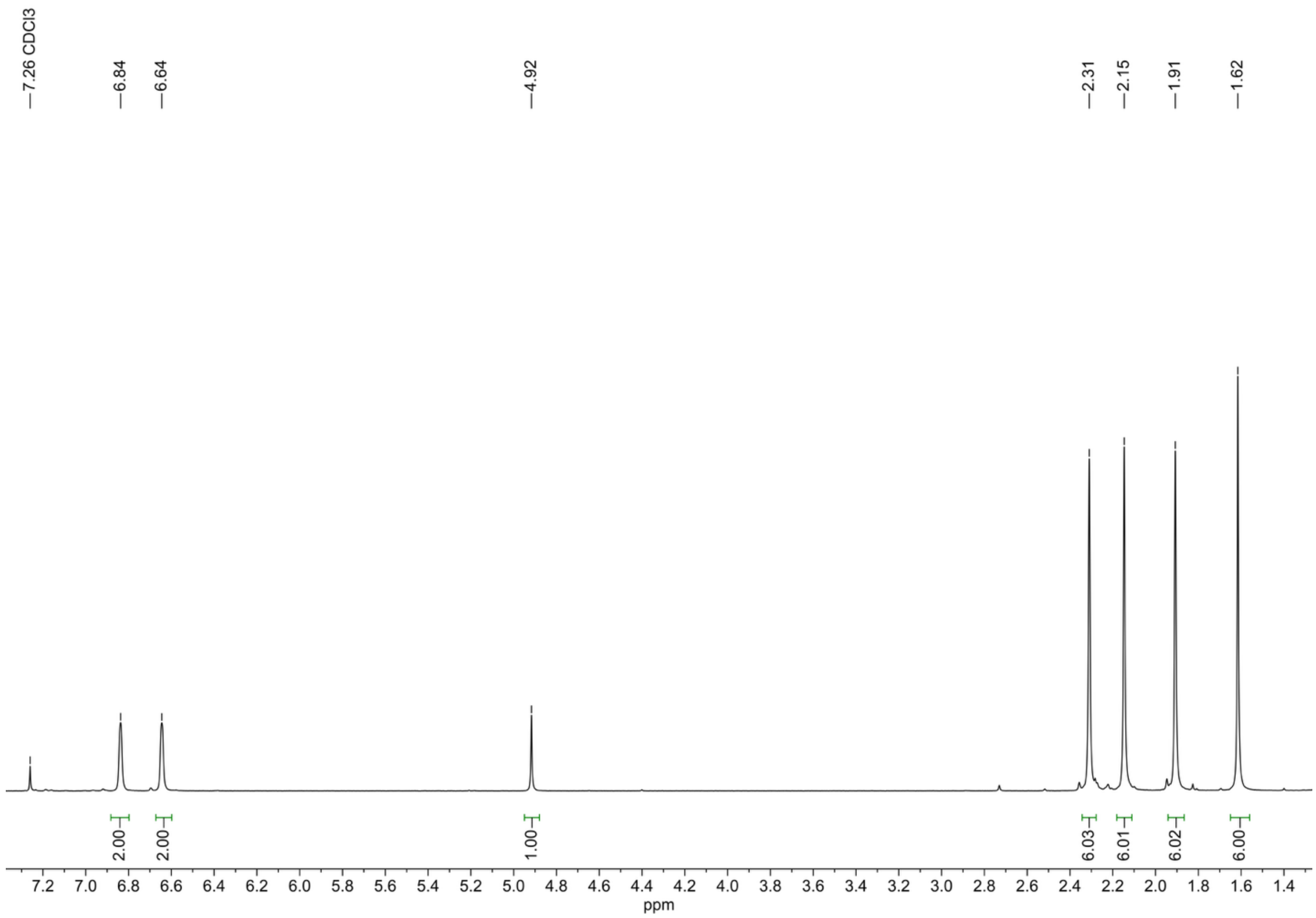

Figure S1. ^{1}H NMR spectrum of **2** in $CDCl_3$.

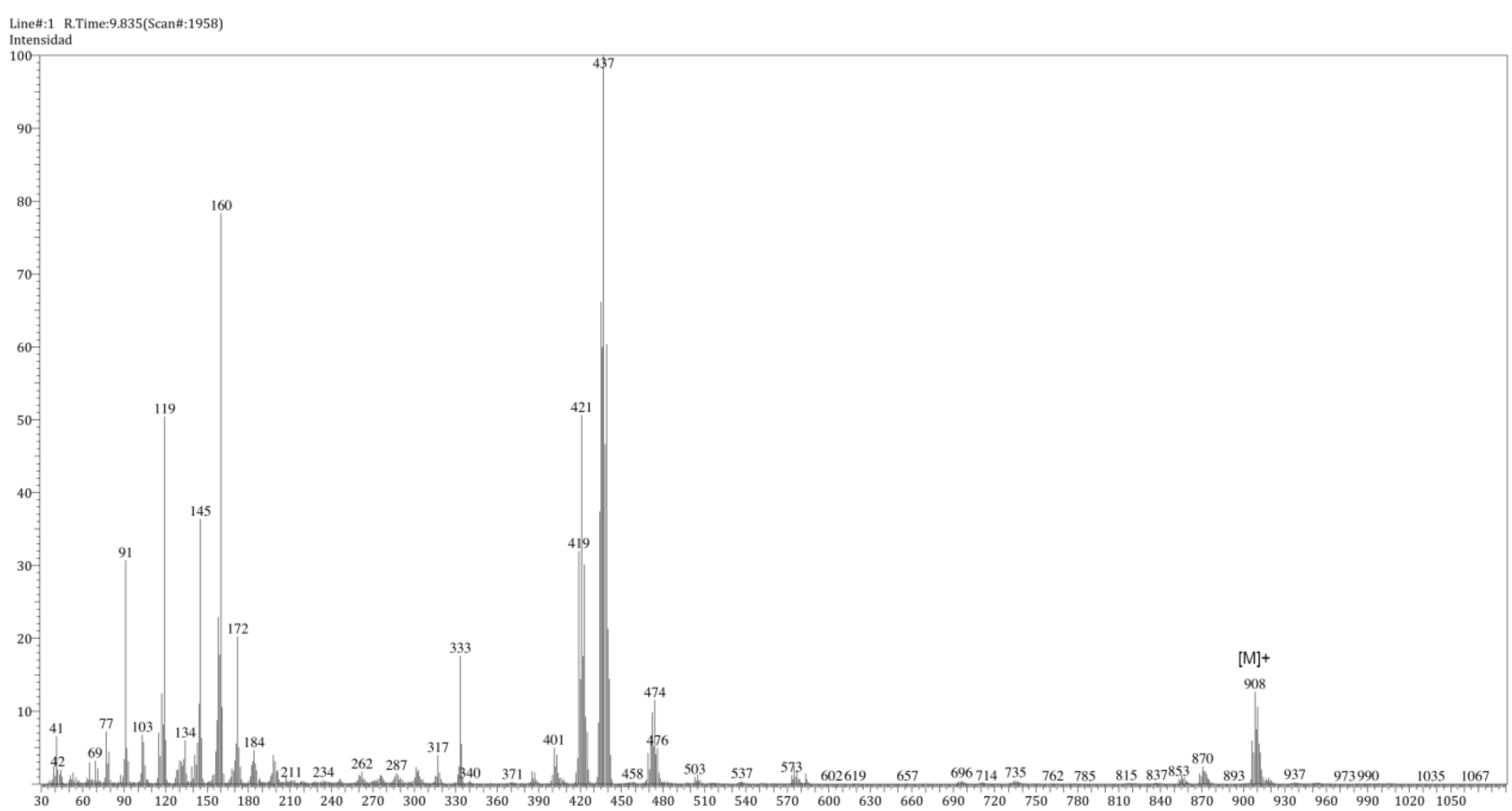

Figure S2. EI-MS spectrum of **2**.

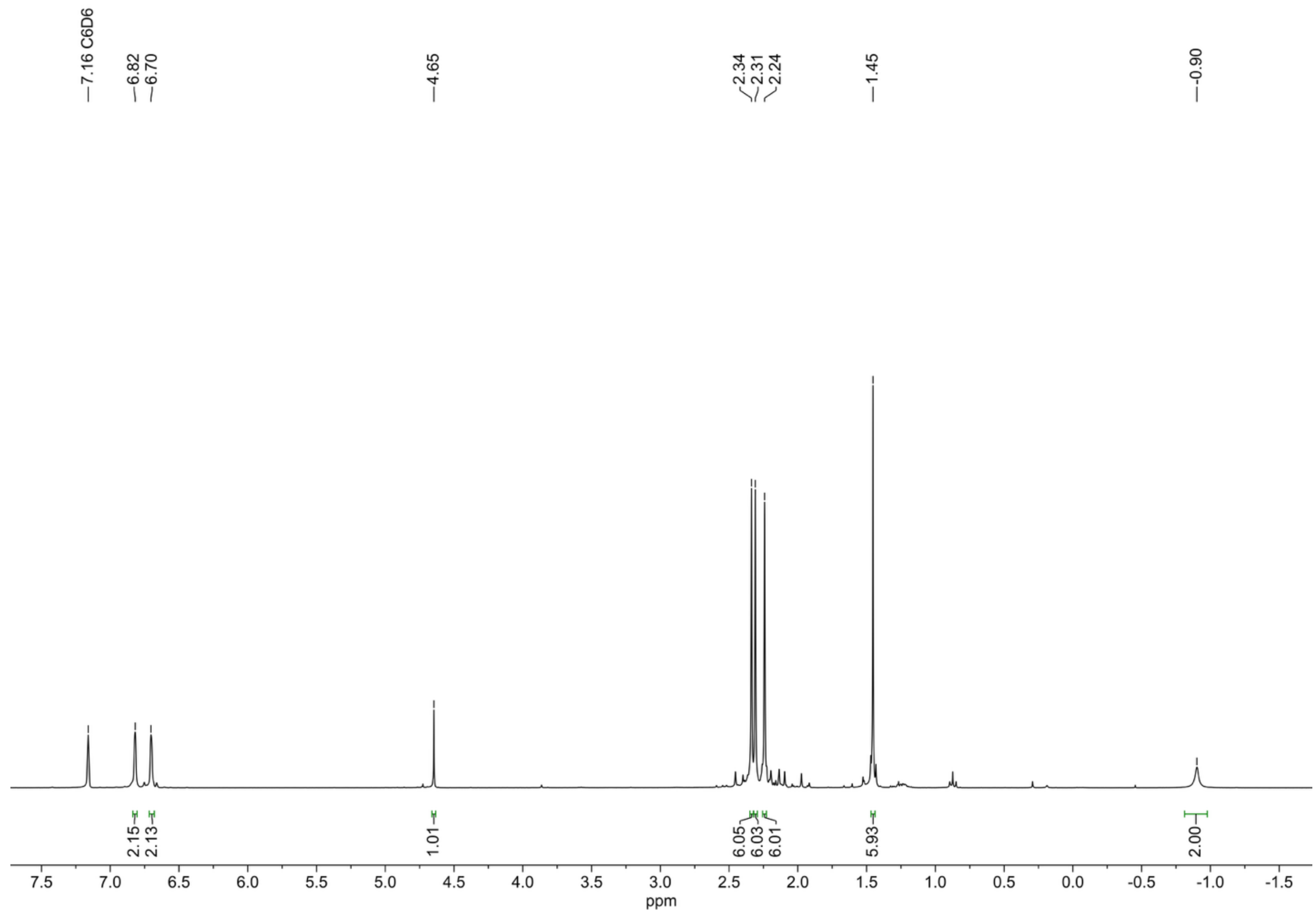

Figure S3. ^{1}H NMR spectrum of **3** in C_6D_6.

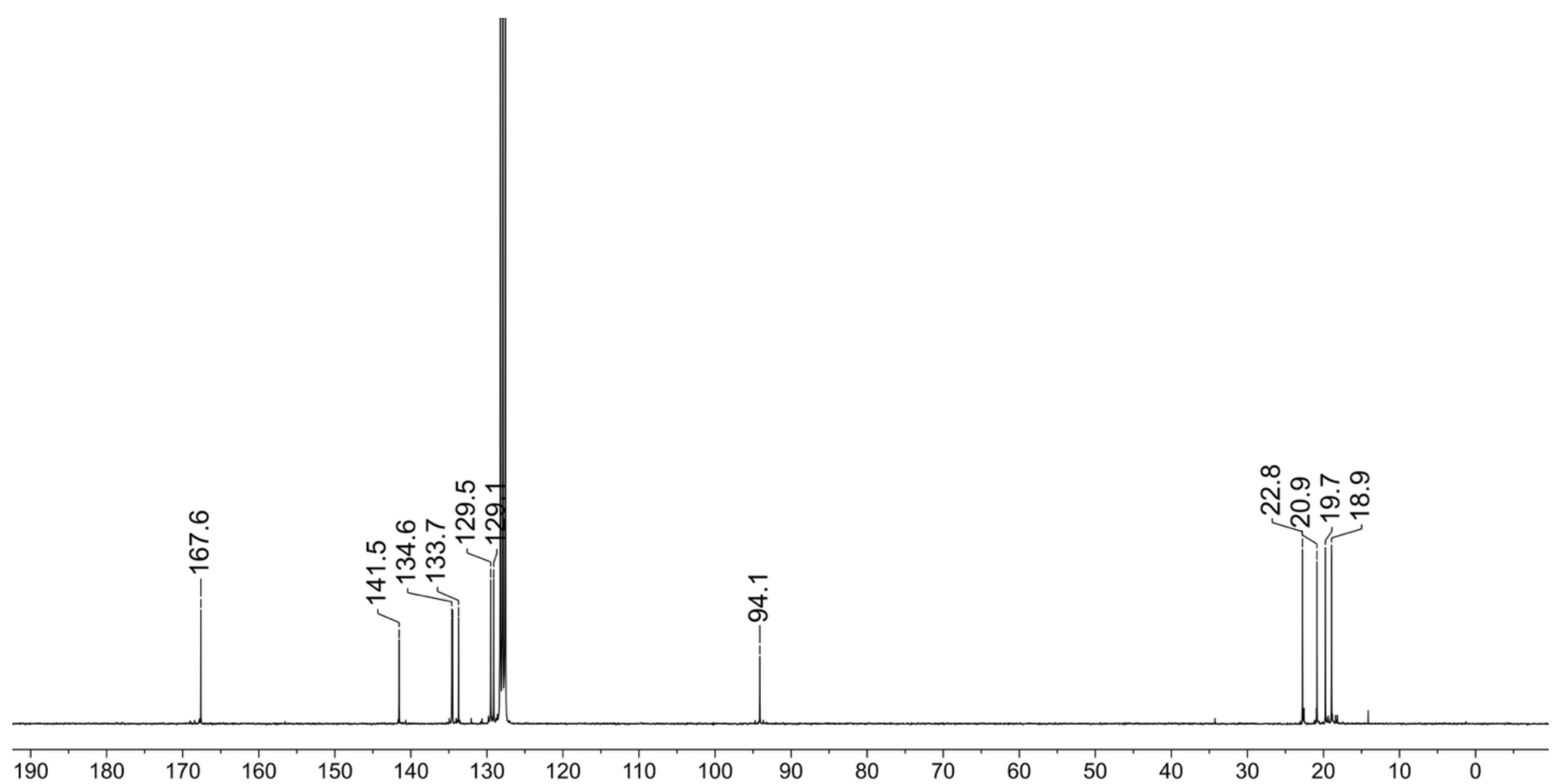

Figure S4. ^{13}C NMR spectrum of **3** in C_6D_6.

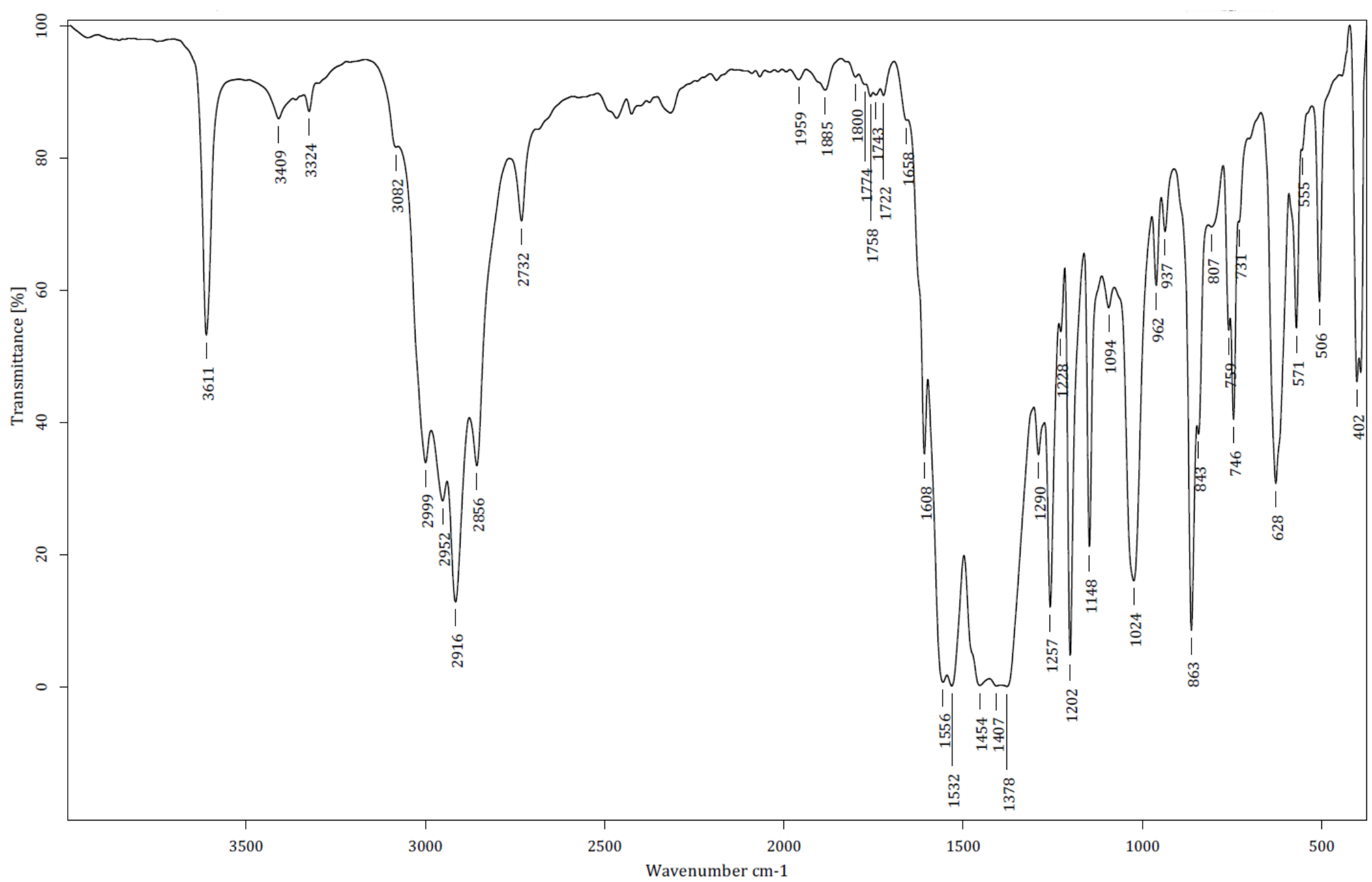

Figure S5. IR (ATR) spectrum of **3**.

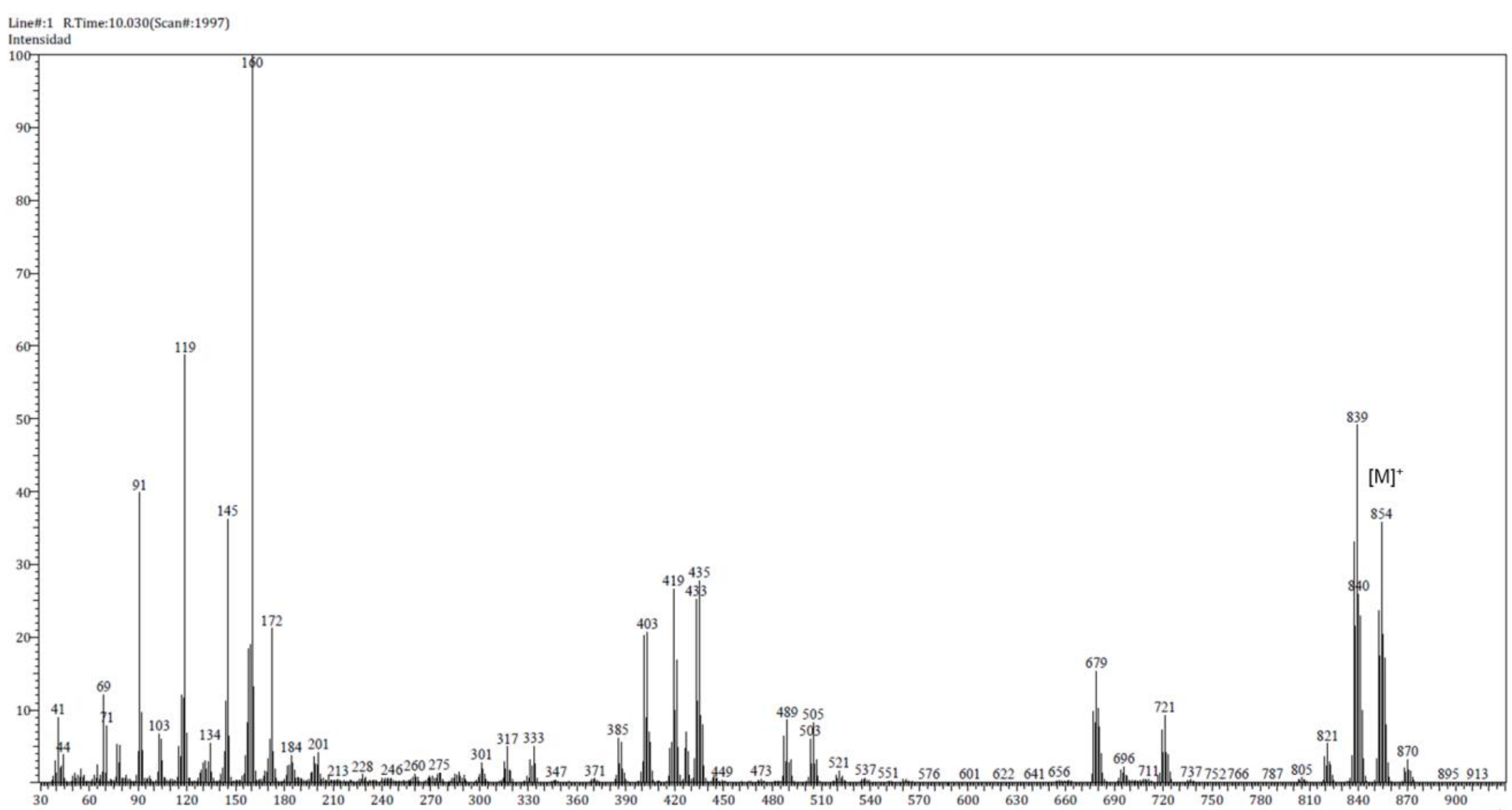

Figure S6. EI-MS spectrum of **3**.

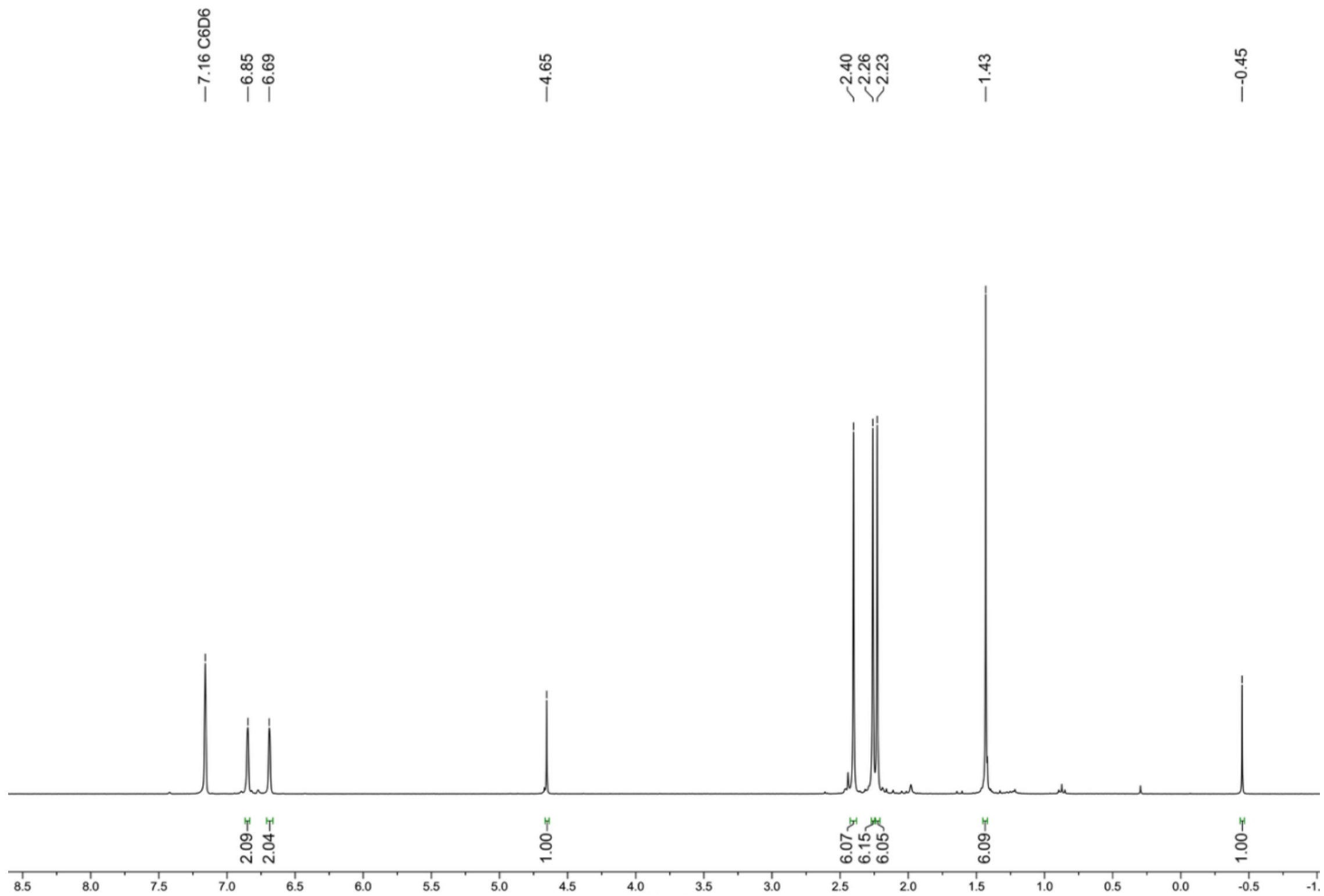

Figure S7. ^{1}H NMR spectrum of **4** in C_6D_6.

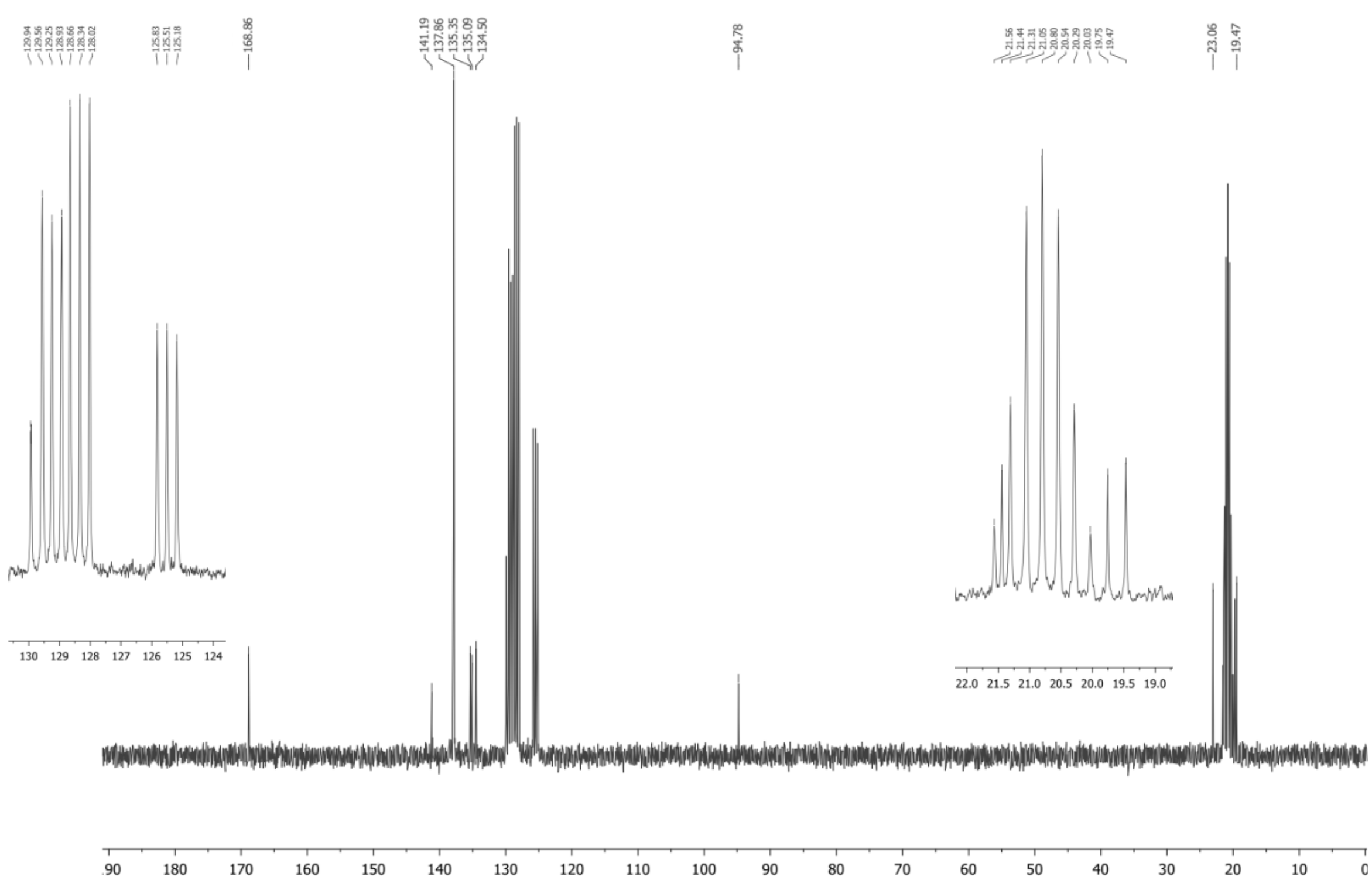

Figure S8. ^{13}C NMR spectrum of **4** in toluene-d_8.

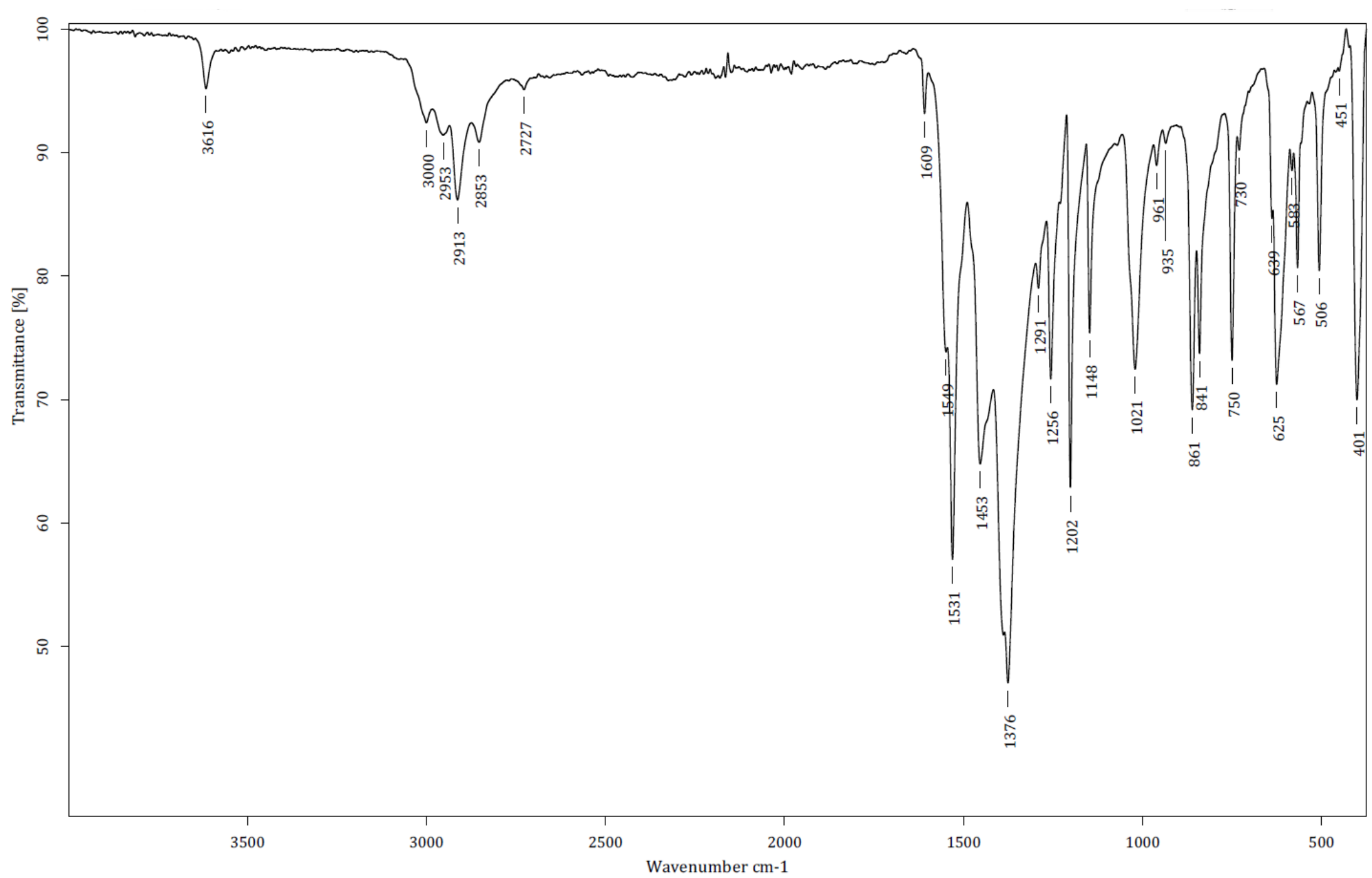

Figure S9. IR (ATR) spectrum of **4**.

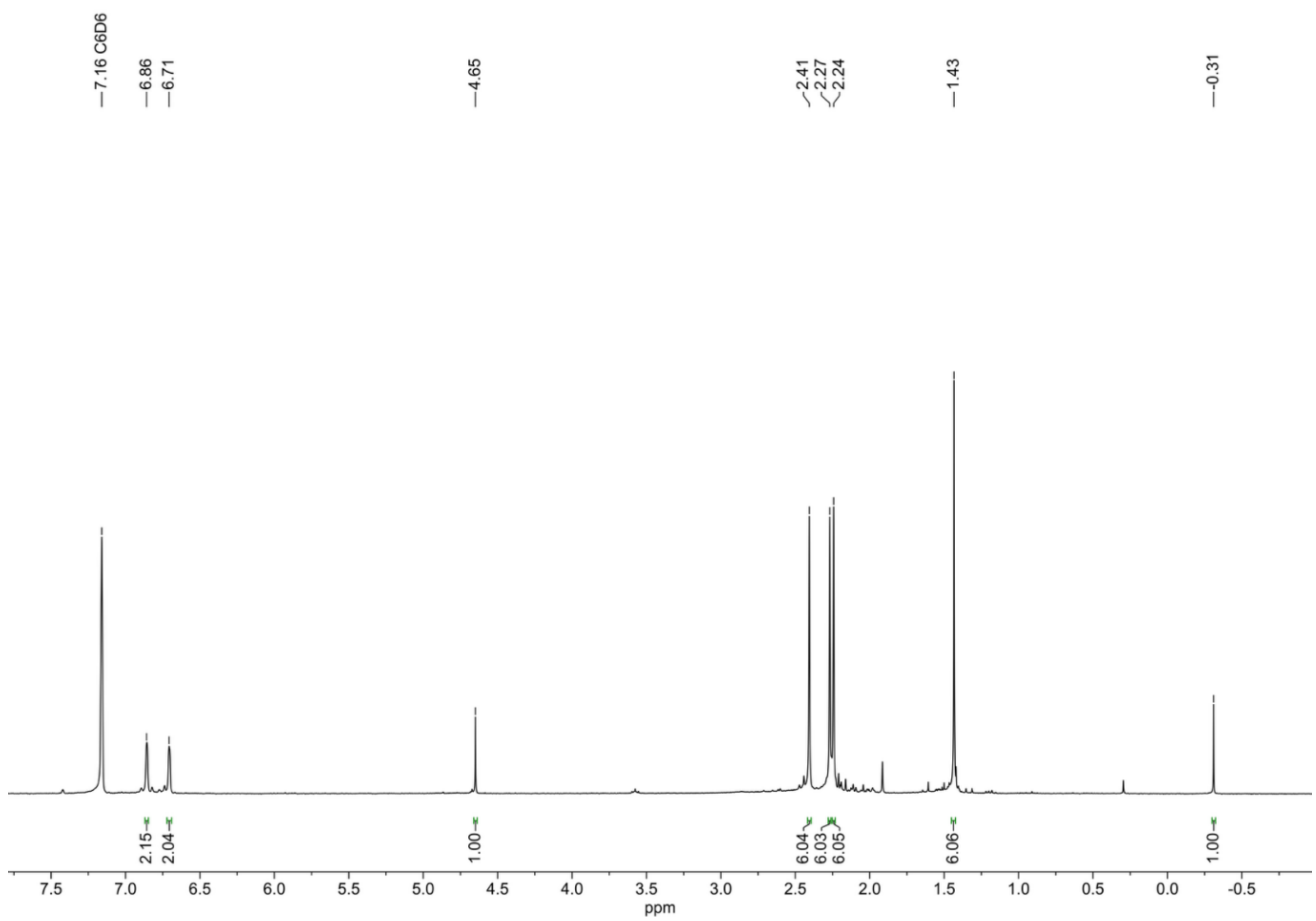

Figure S10. ^{1}H NMR spectrum of **5** in C_6D_6.

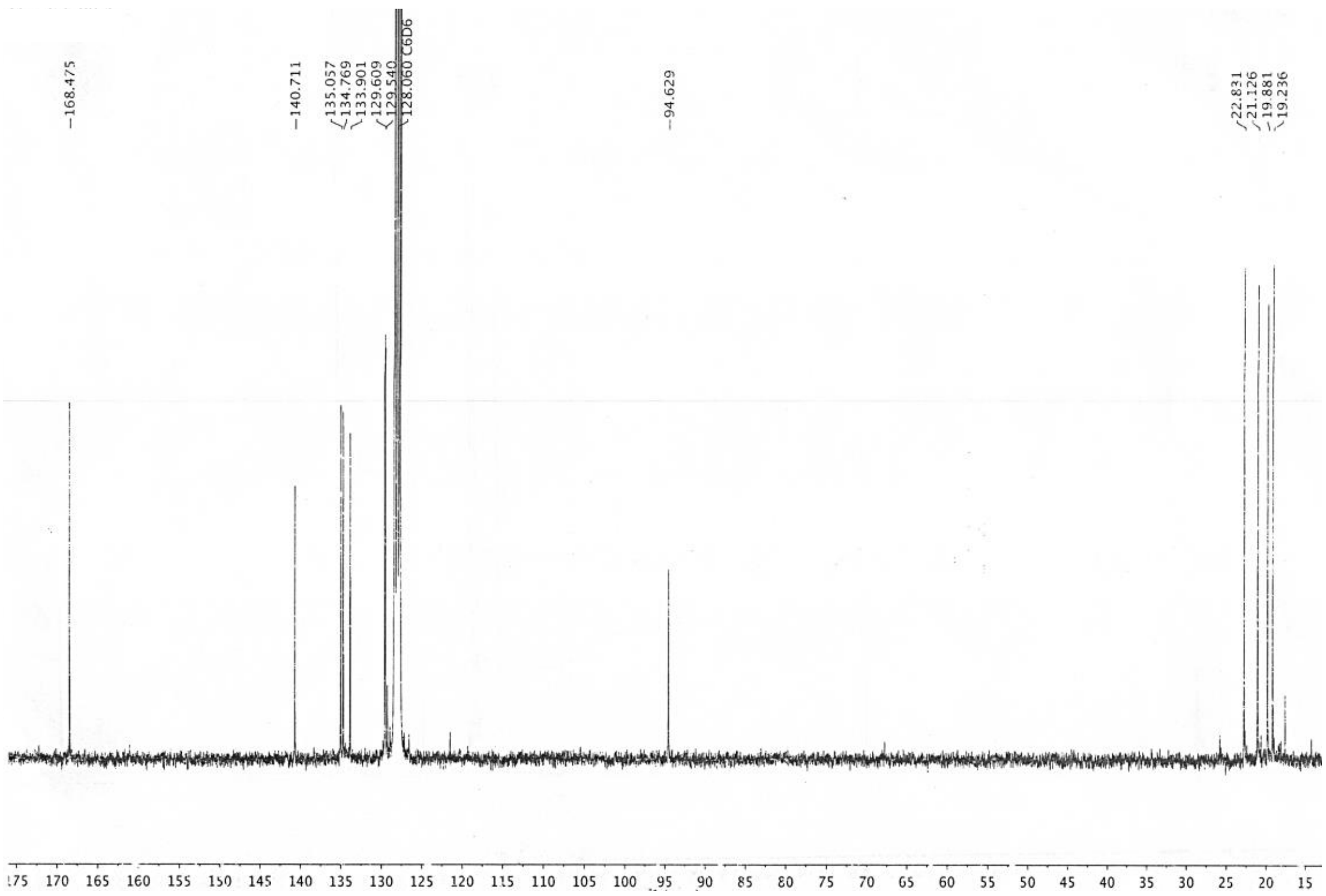

Figure S11. ^{13}C NMR spectrum of **5** in C_6D_6.

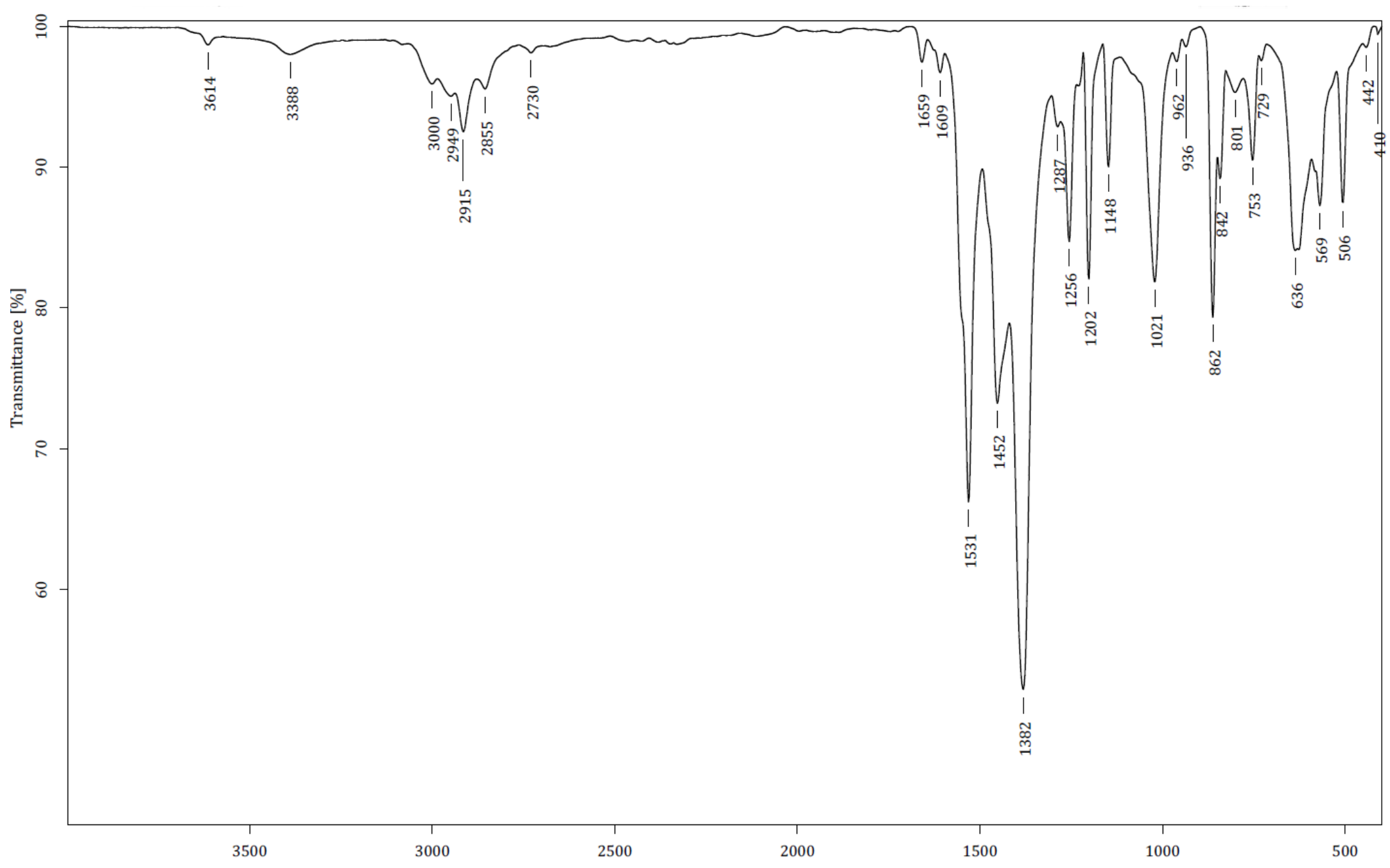

Figure S12. IR (ATR) spectrum of **5**.

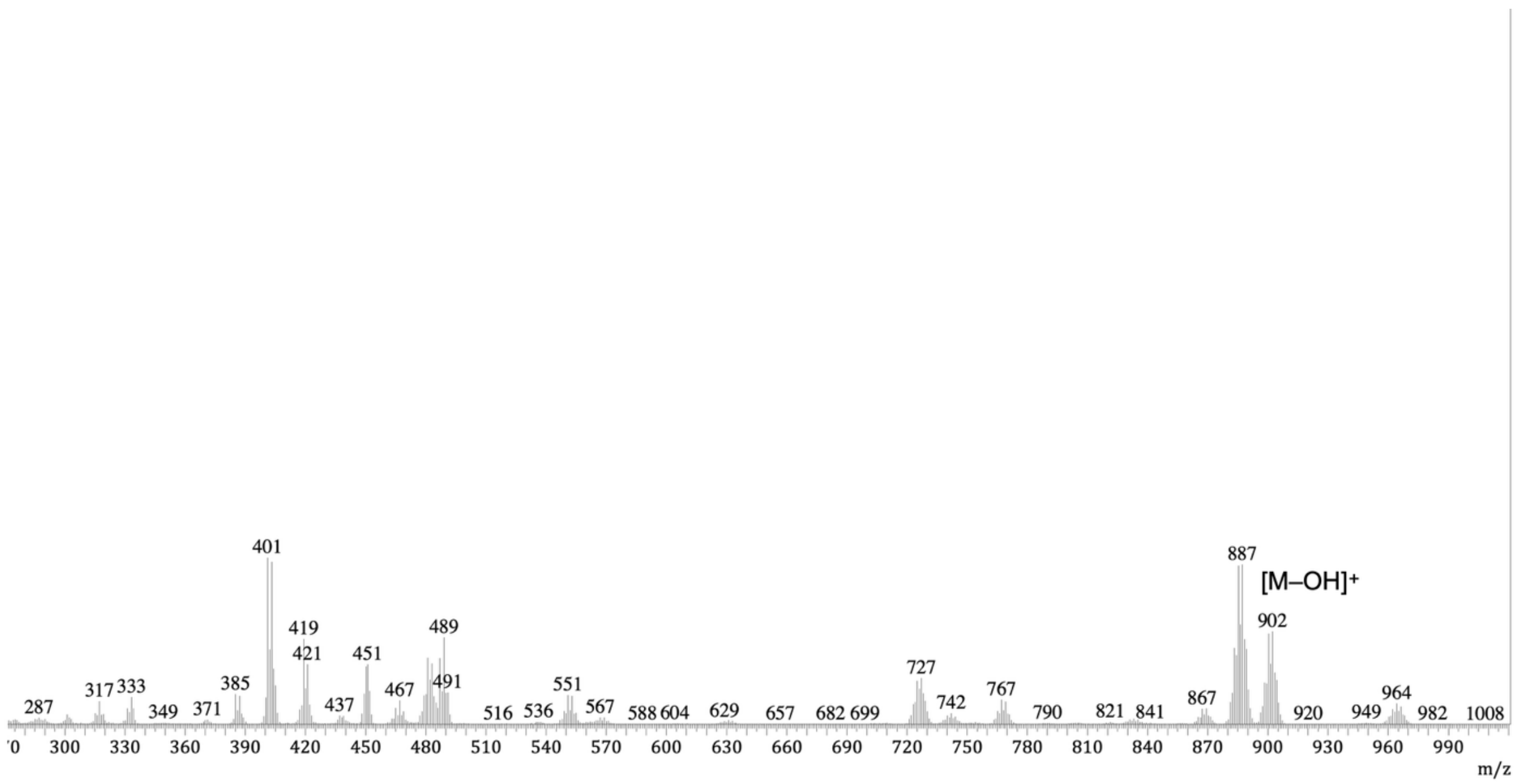

Figure S13. EI-MS spectrum of **5**.

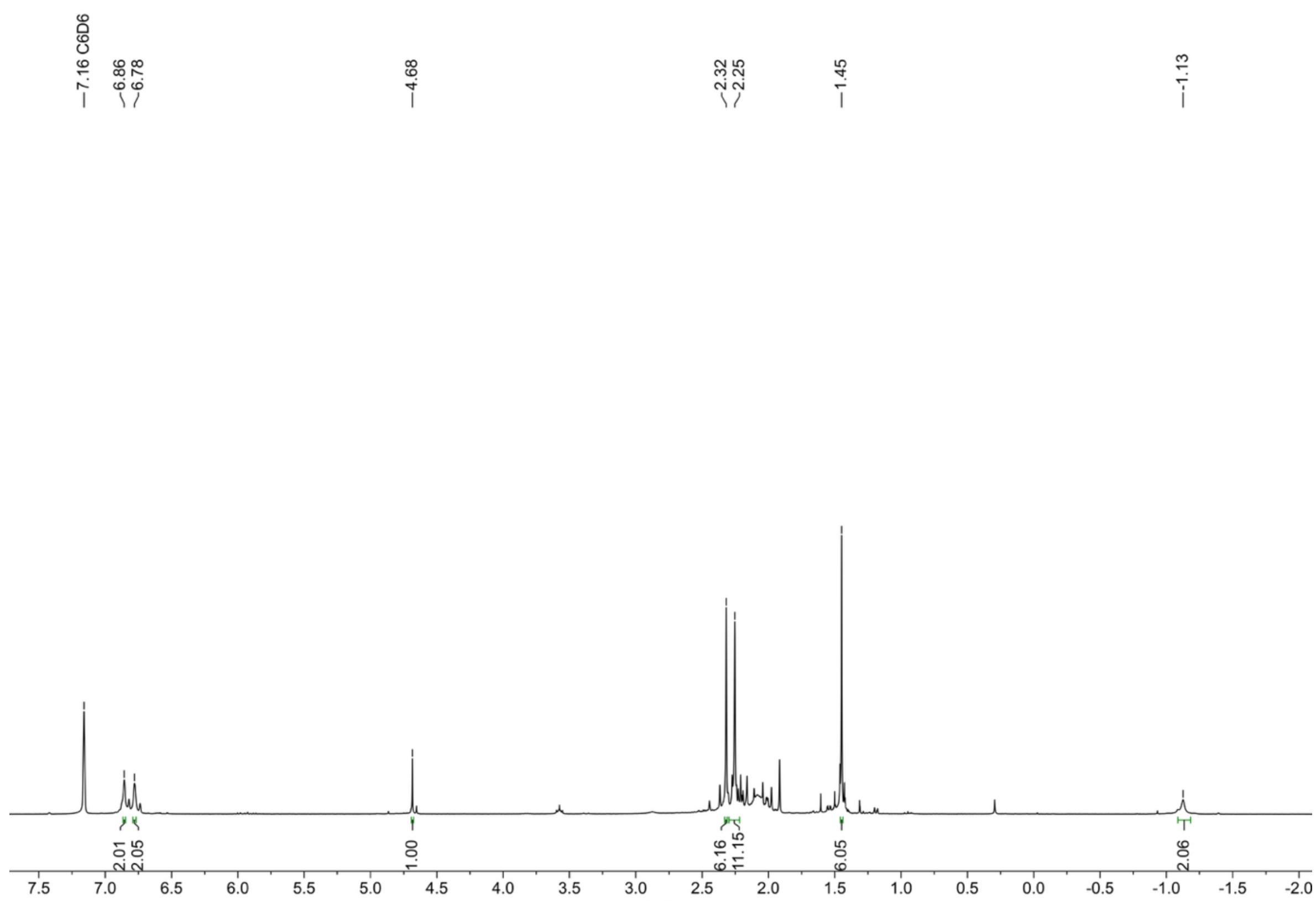

Figure S14. ^{1}H NMR spectrum of **6** in C_6D_6.

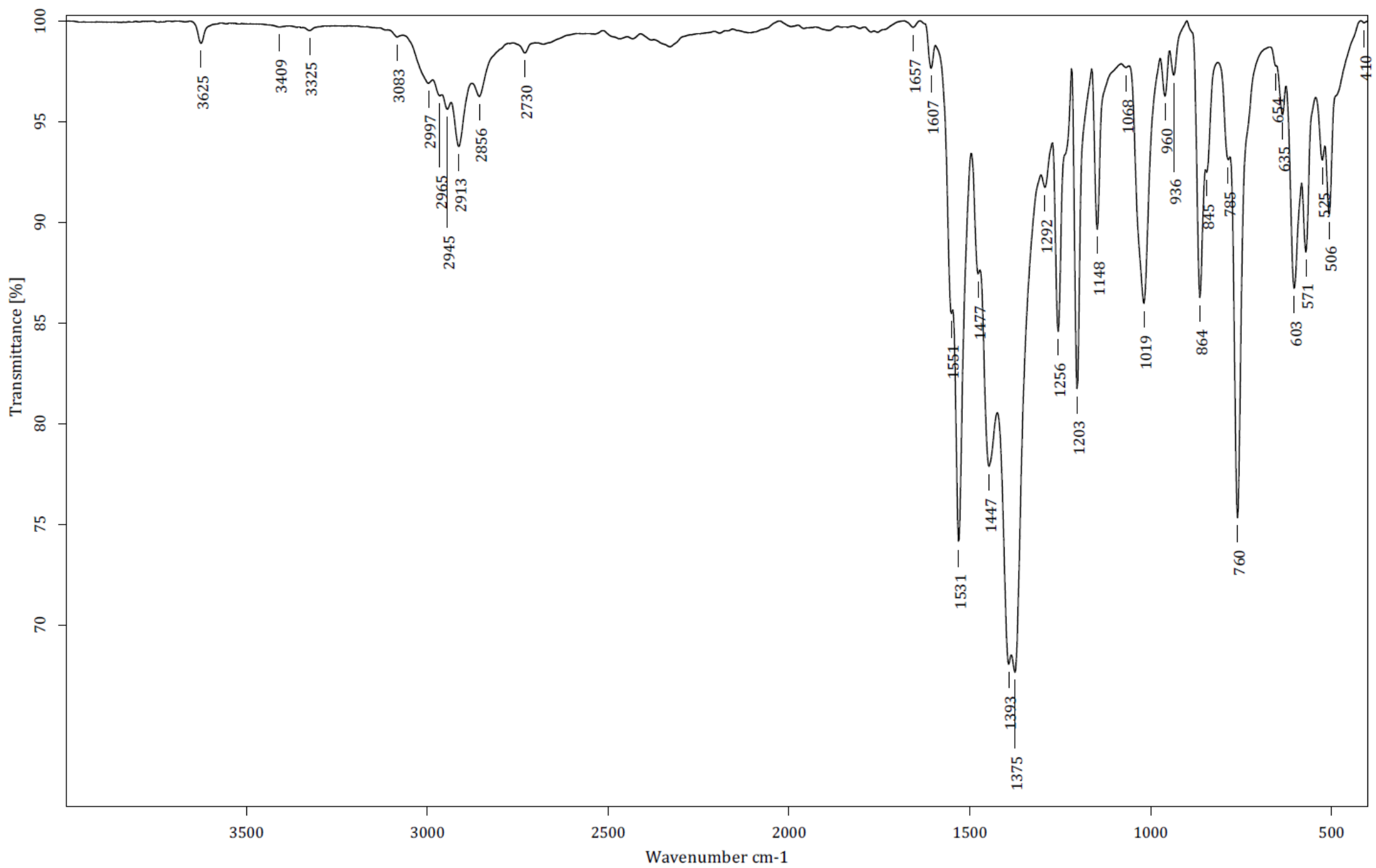

Figure S15. IR (ATR) spectrum of **6**.

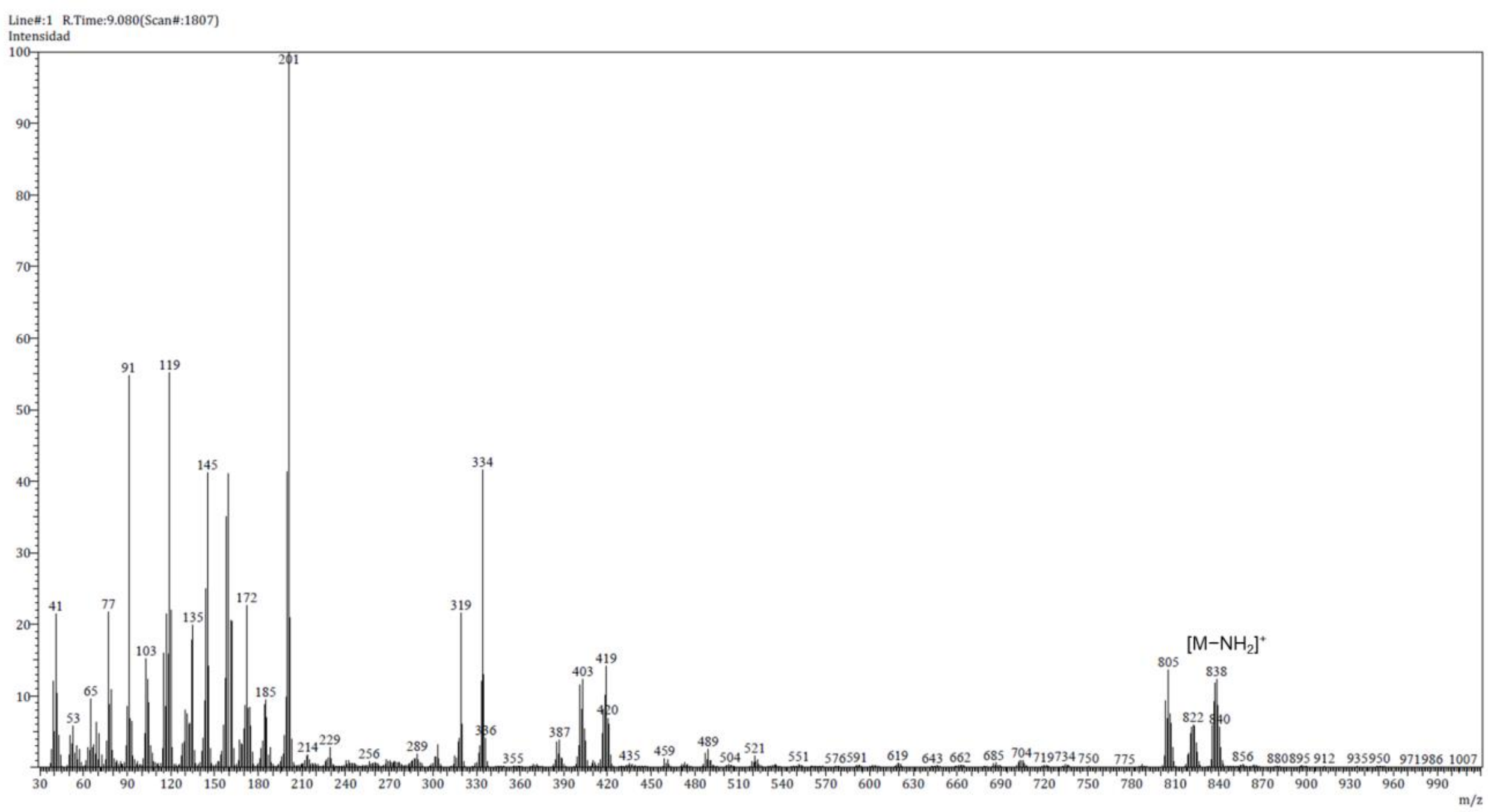

Figure S16. EI-MS spectrum of **6**.

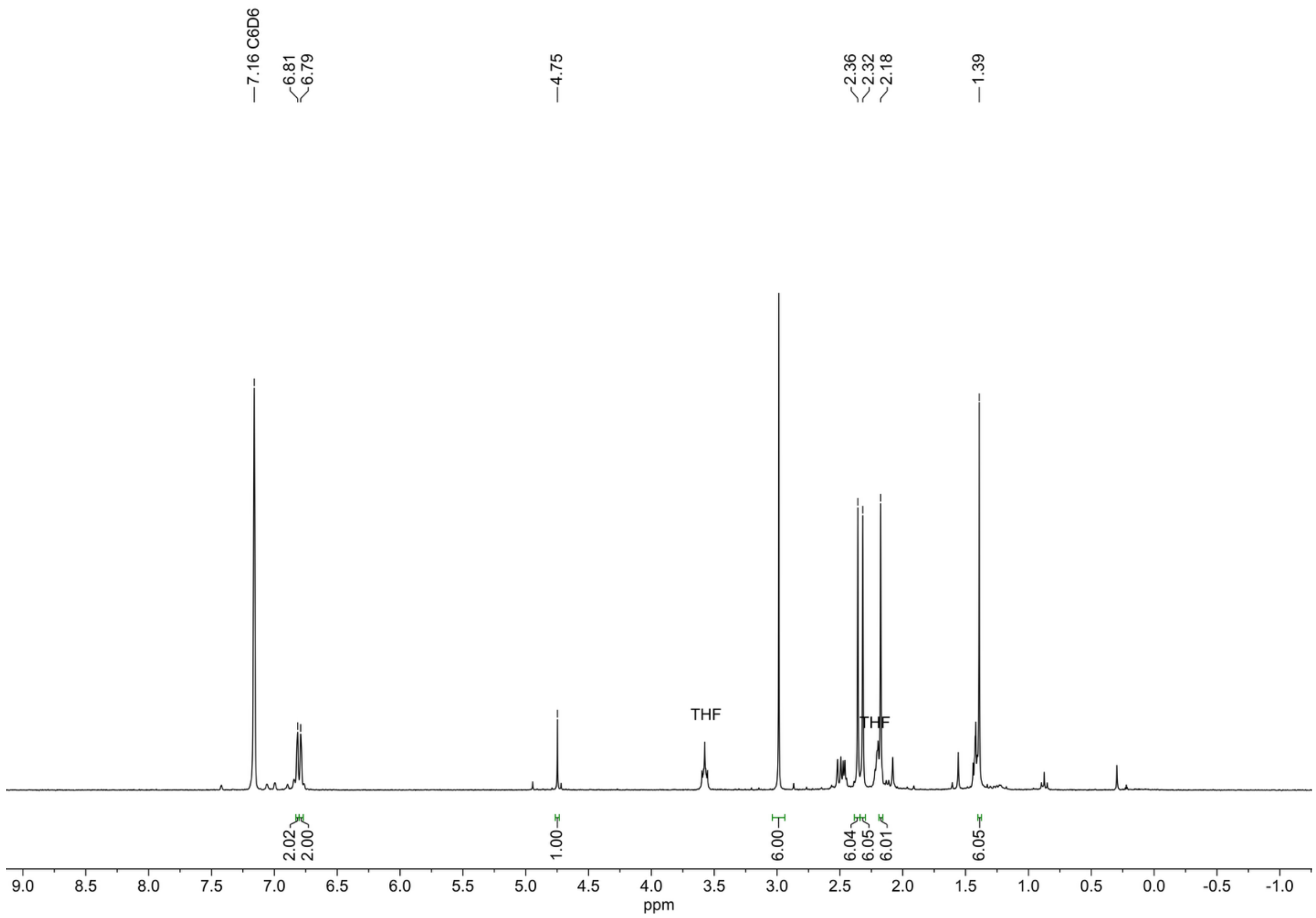

Figure S17. ^{1}H NMR spectrum of **9** in C_6D_6.

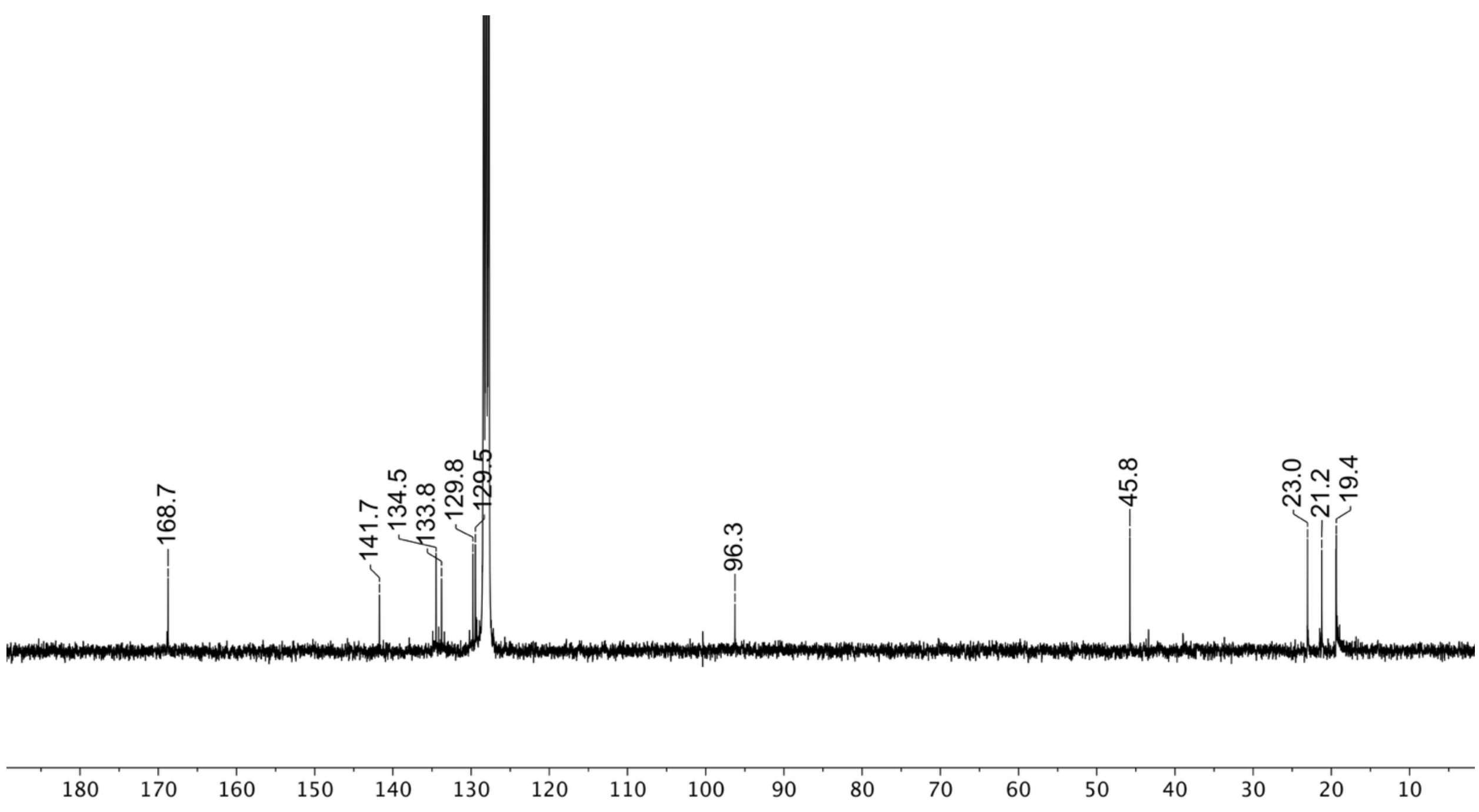

Figure S18. ^{13}C NMR spectrum of **9** in C_6D_6.

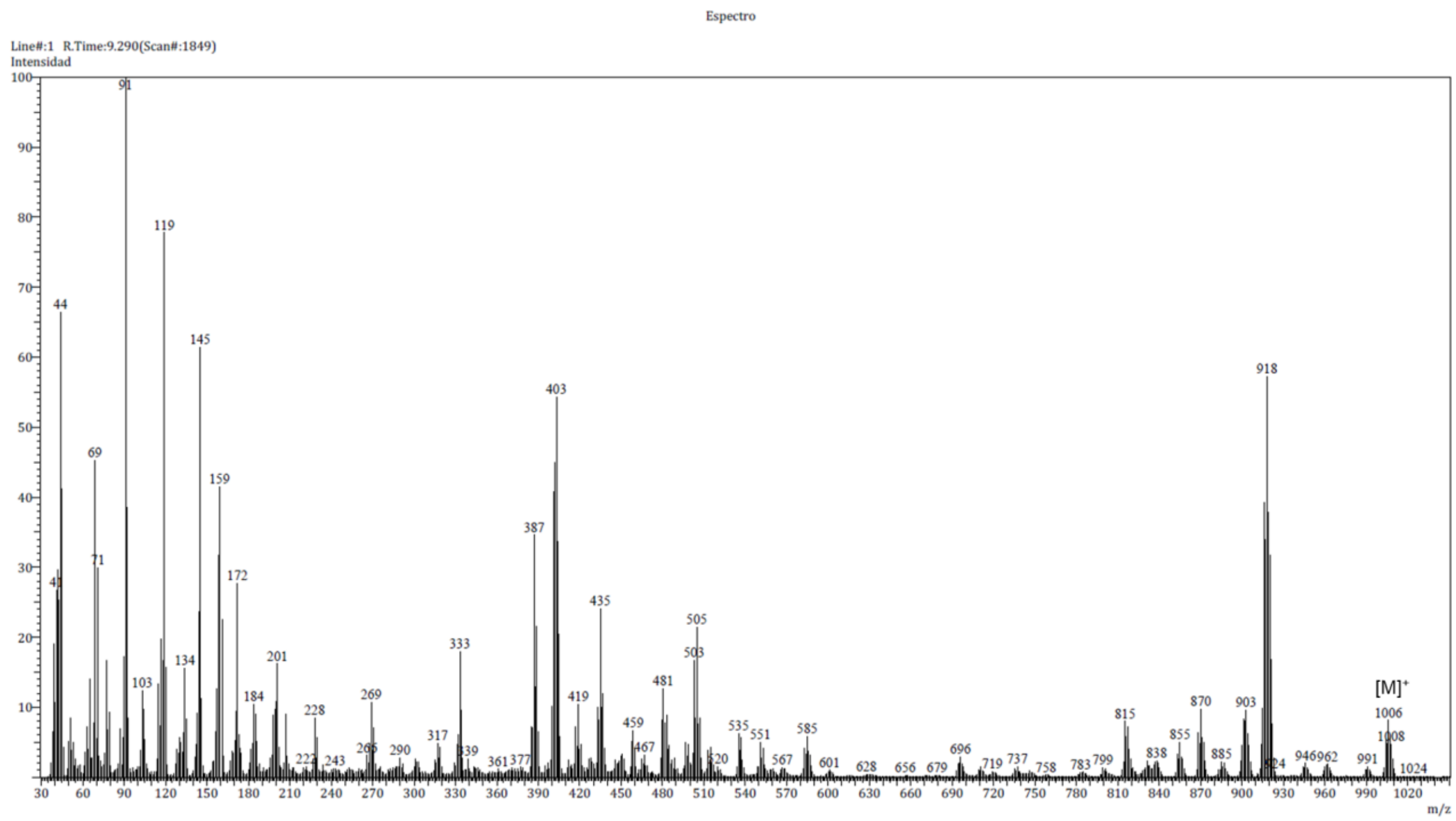

Figure S19. EI-MS spectrum of **9**.

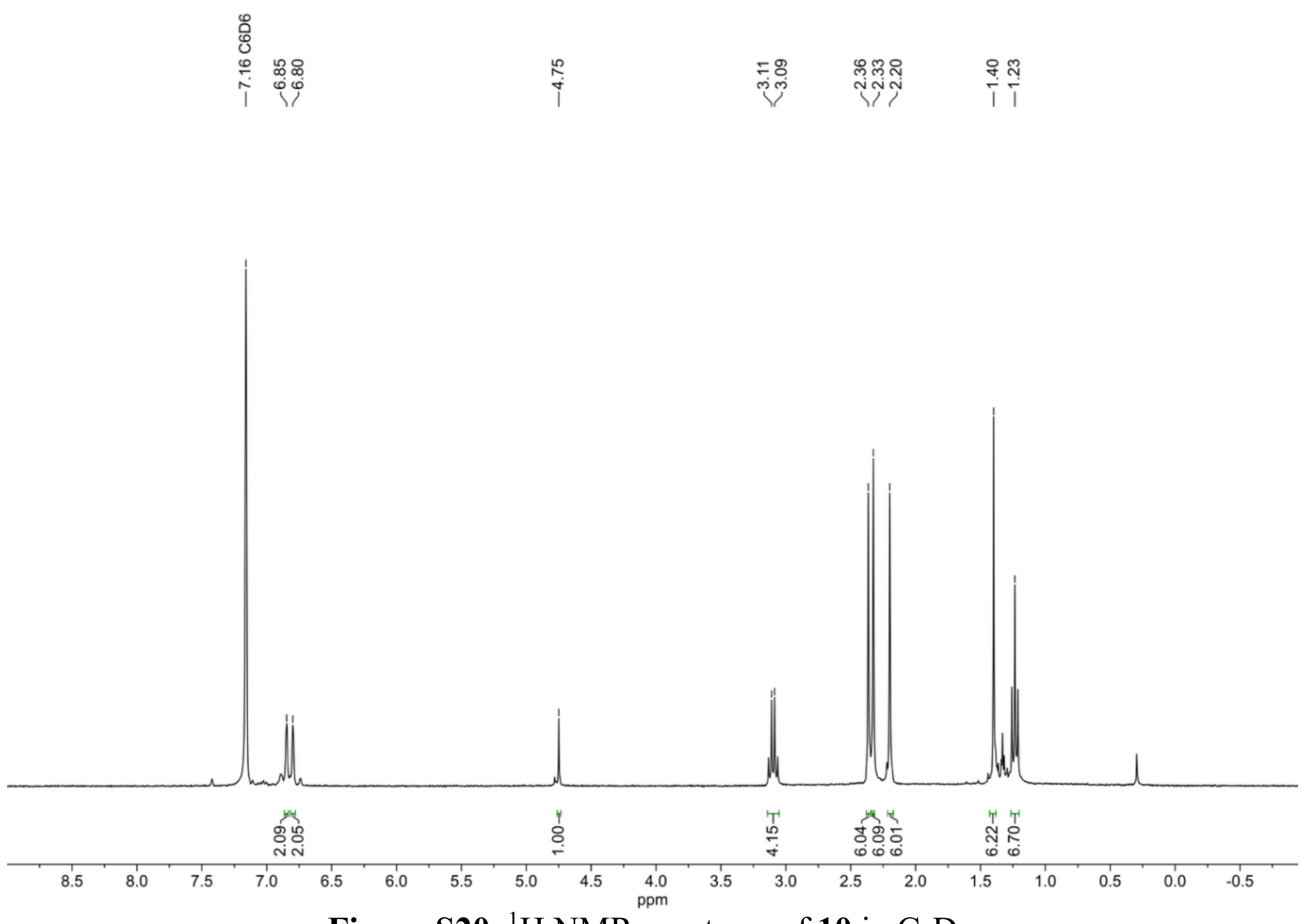

Figure S20. ^{1}H NMR spectrum of **10** in C_6D_6.

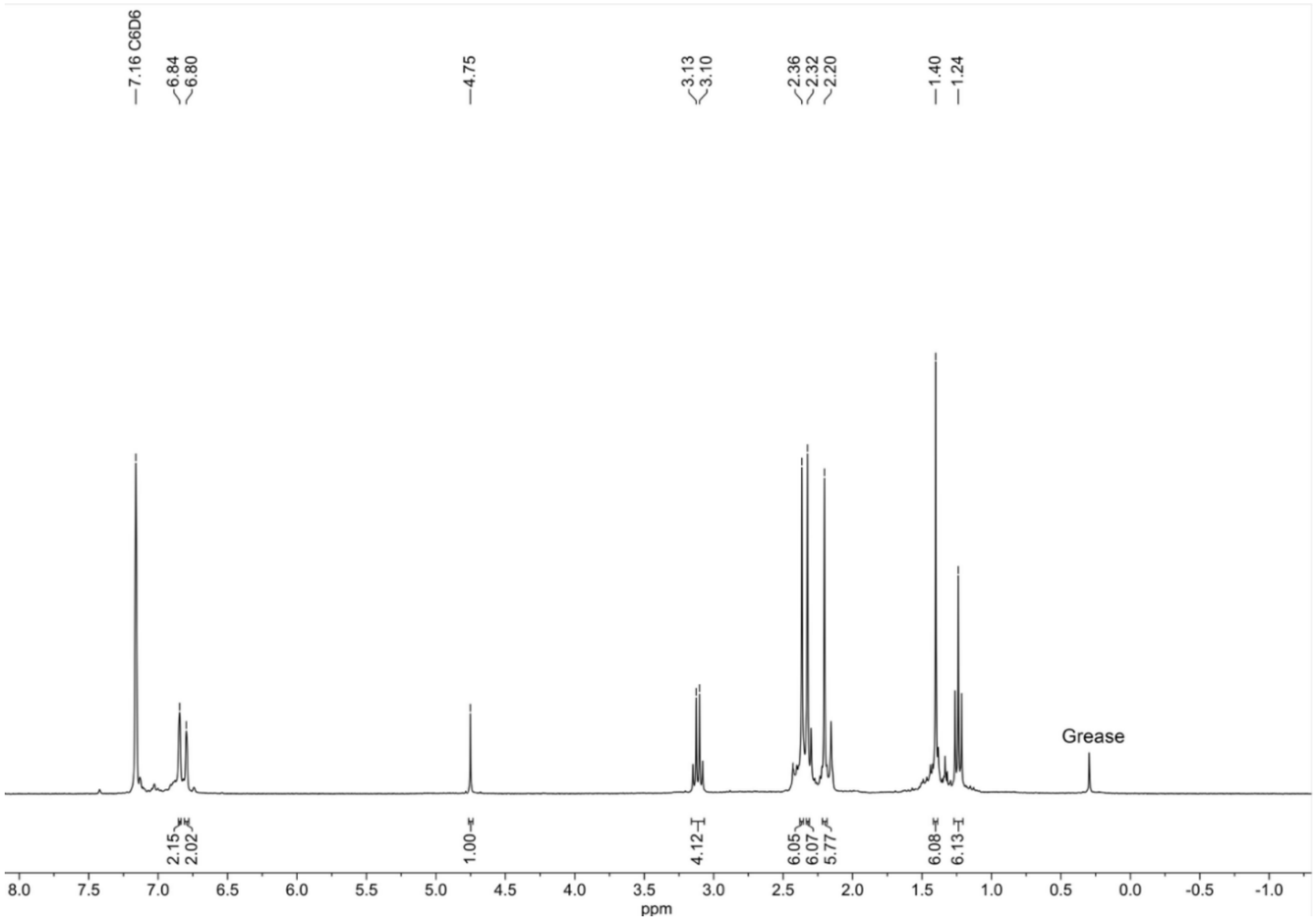

Figure S21. ^{1}H NMR spectrum of **11** in C_6D_6.

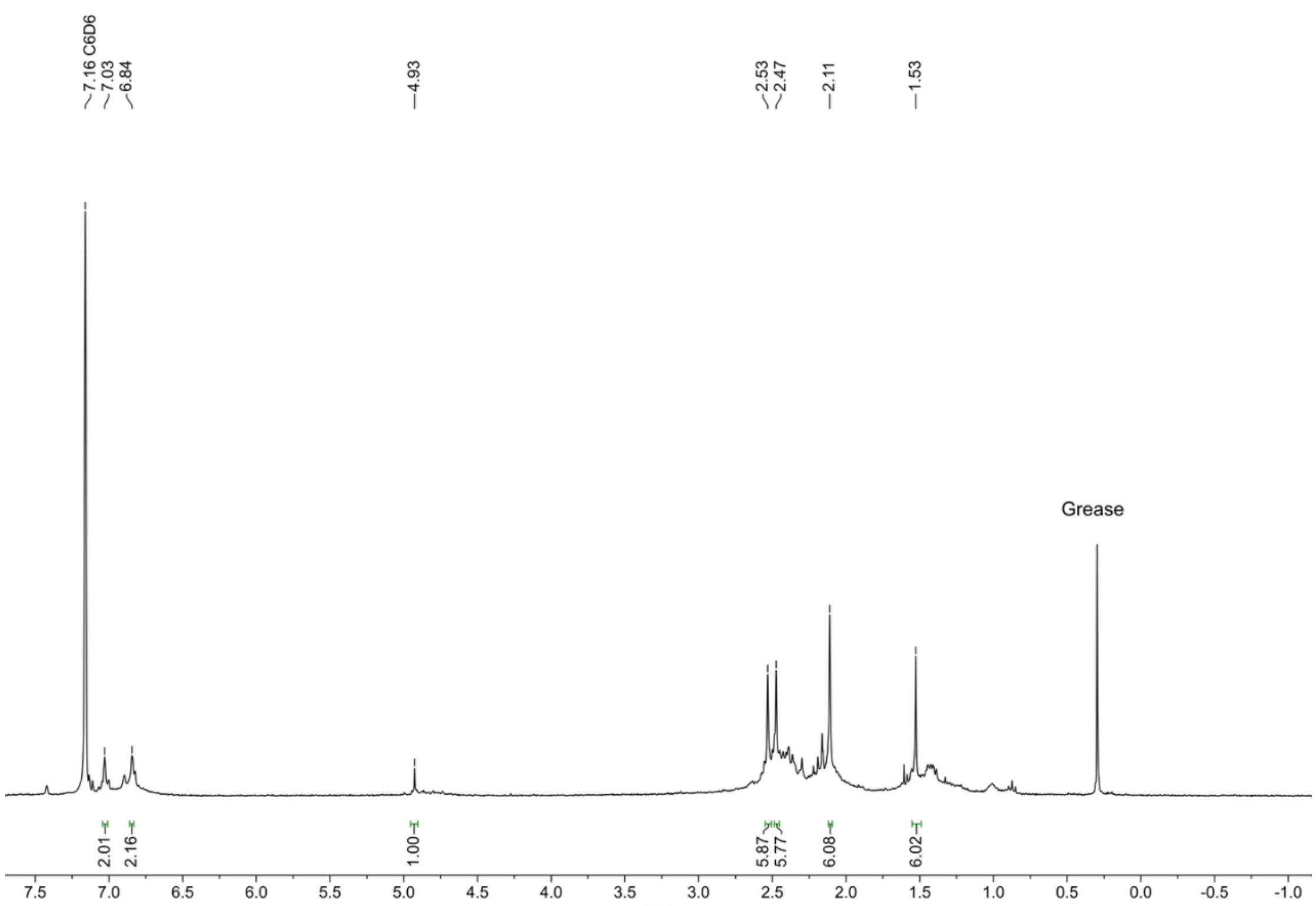

Figure S22. ^{1}H NMR spectrum of **12** in C_6D_6.

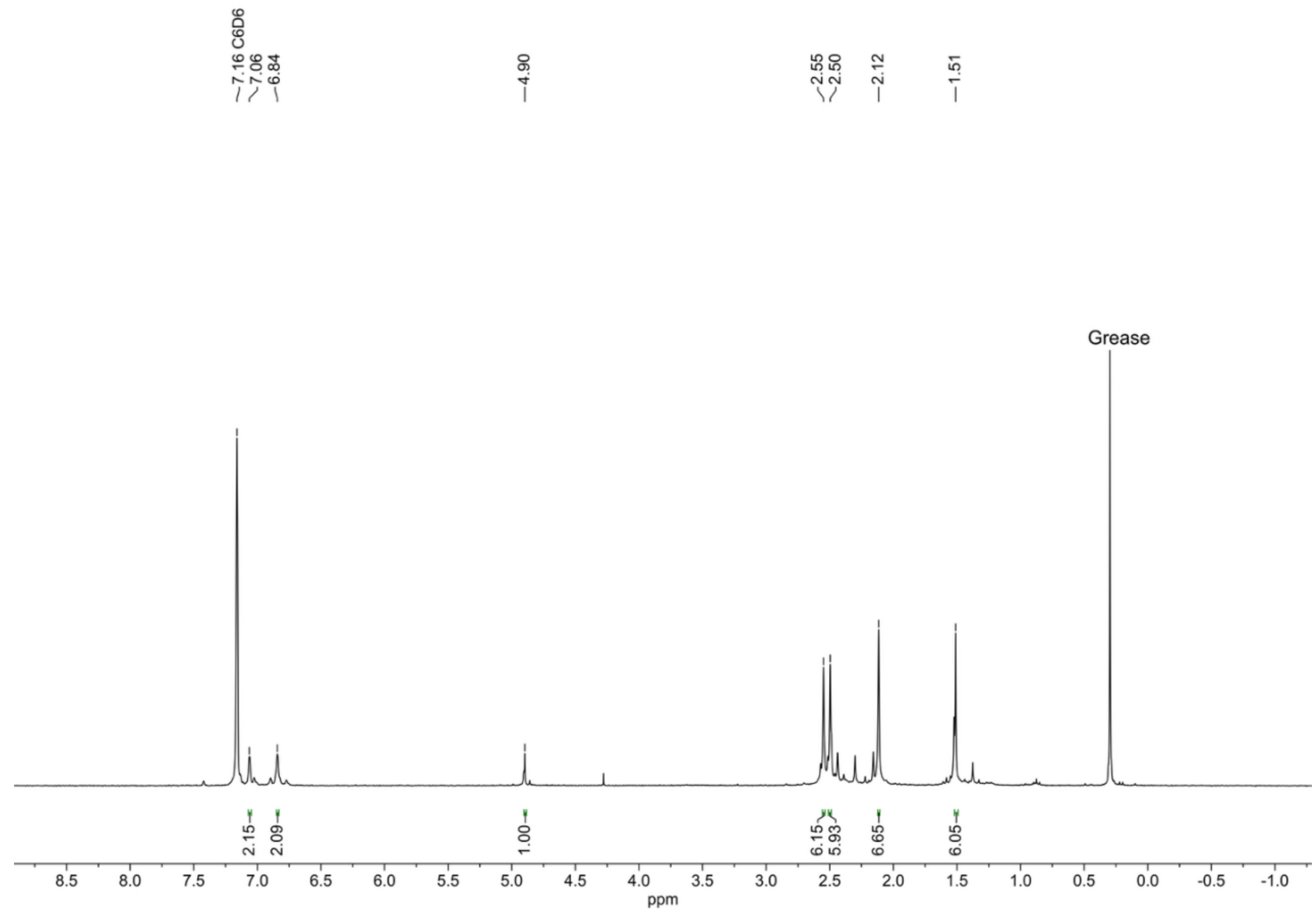

Figure S23. ^{1}H NMR spectrum of **13** in C_6D_6.

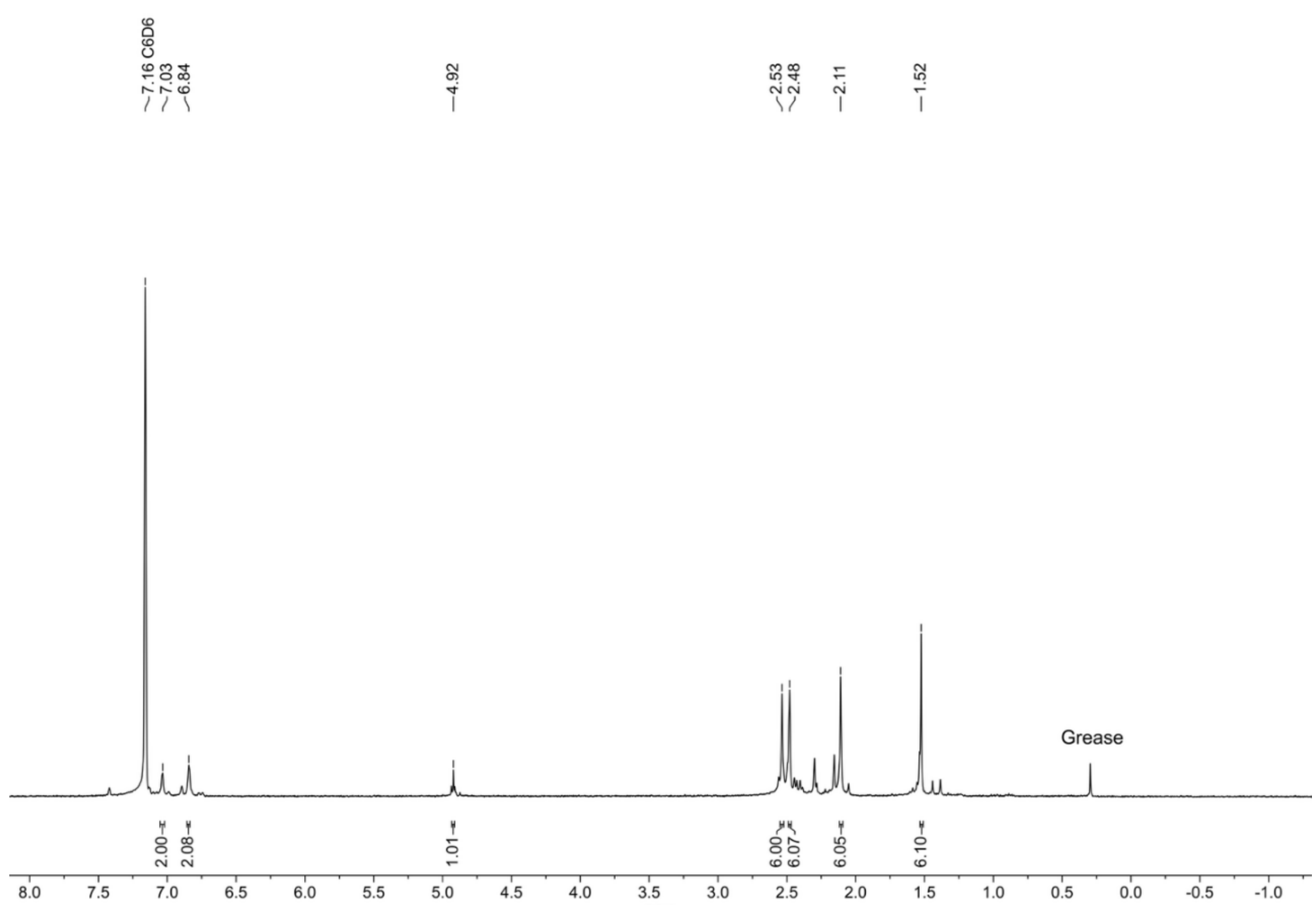

Figure S24. ^{1}H NMR spectrum of **14** in C_6D_6.

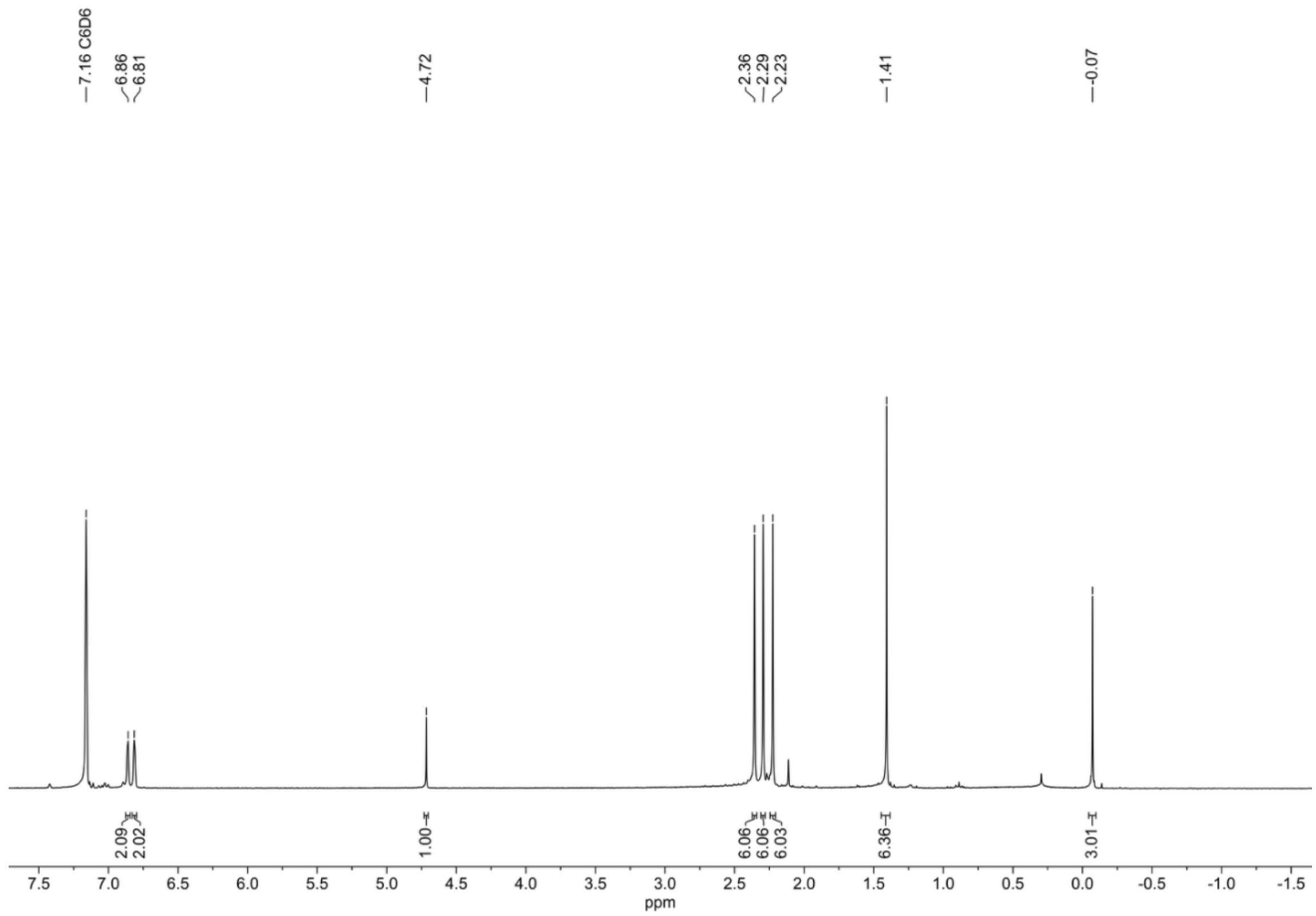

Figure S25. ^{1}H NMR spectrum of **15** in C_6D_6.

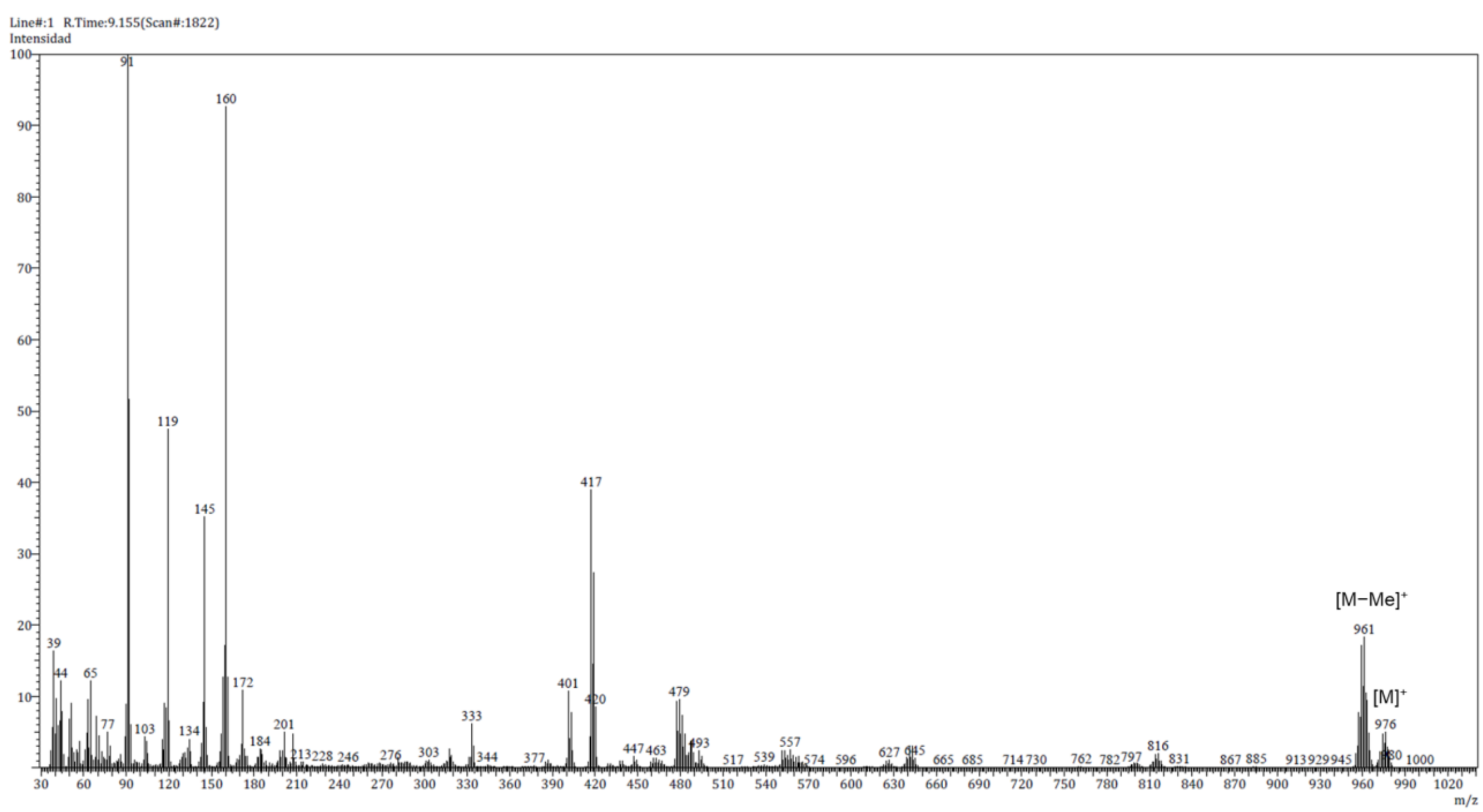

Figure S26. EI-MS spectrum of **15**.

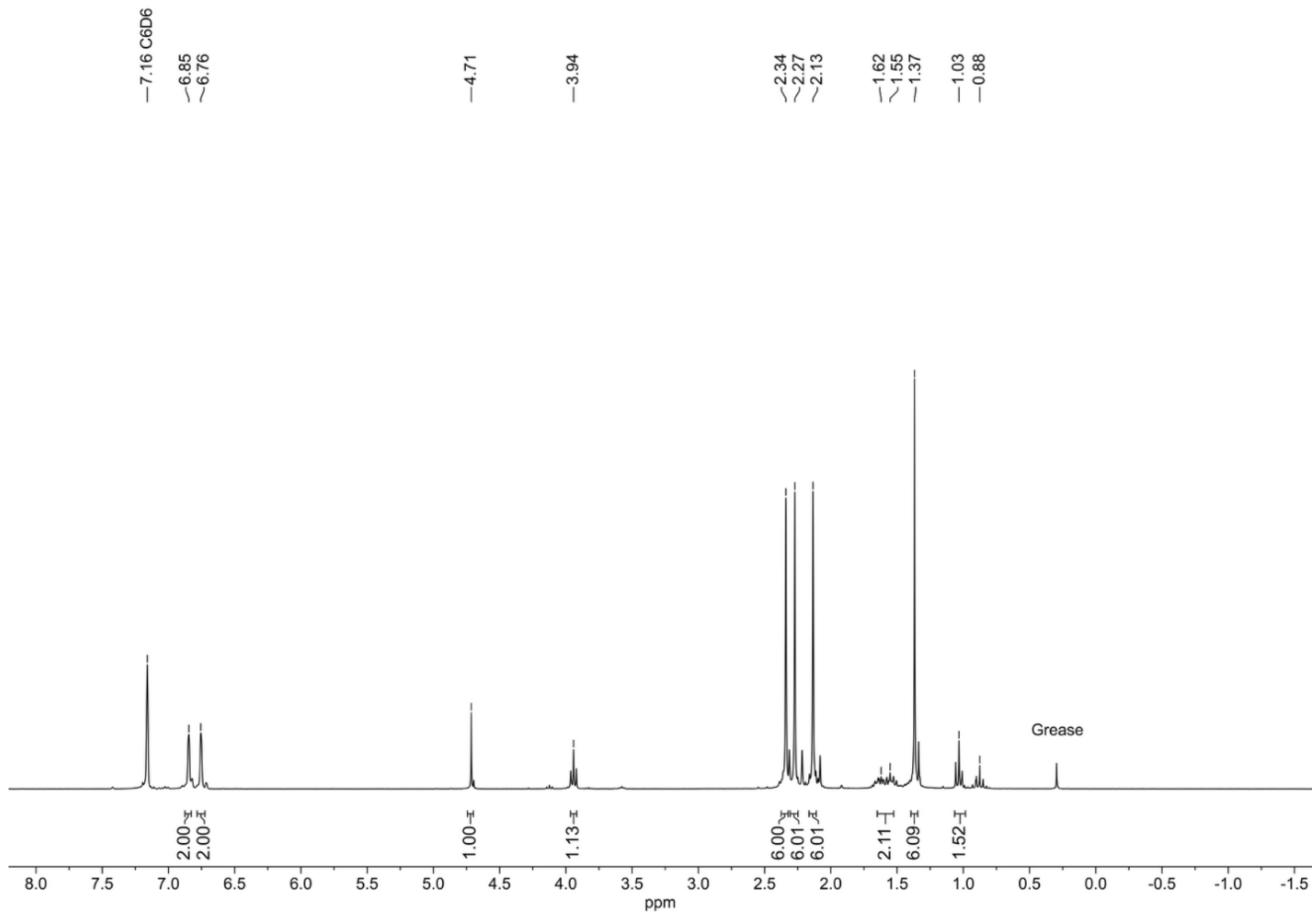

Figure S27. ^{1}H NMR spectrum of **17** in C_6D_6.

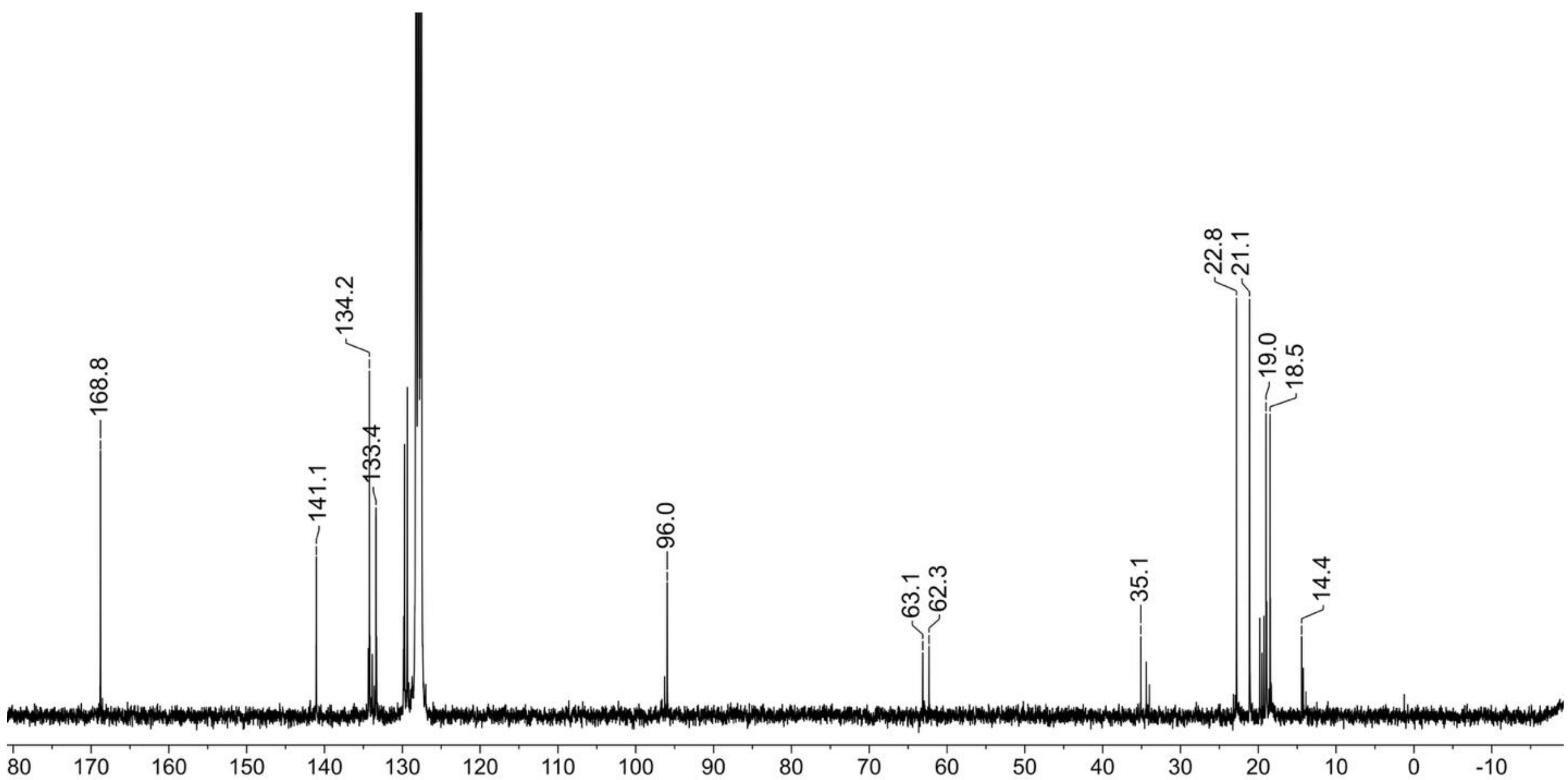

Figure S28. ^{13}C NMR spectrum of **17** in C_6D_6.

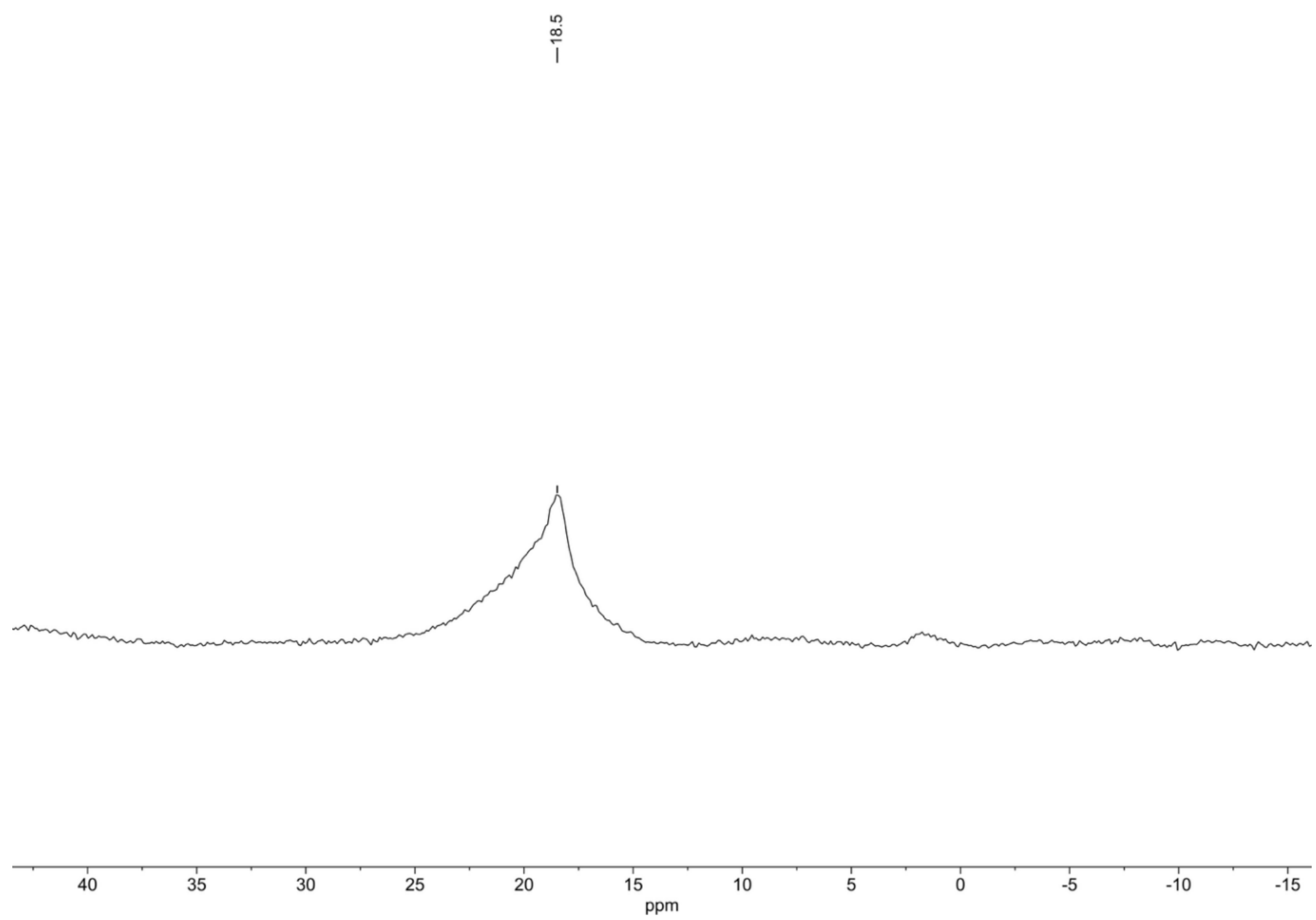

Figure S29. ^{11}B NMR spectrum of **17** in C_6D_6.

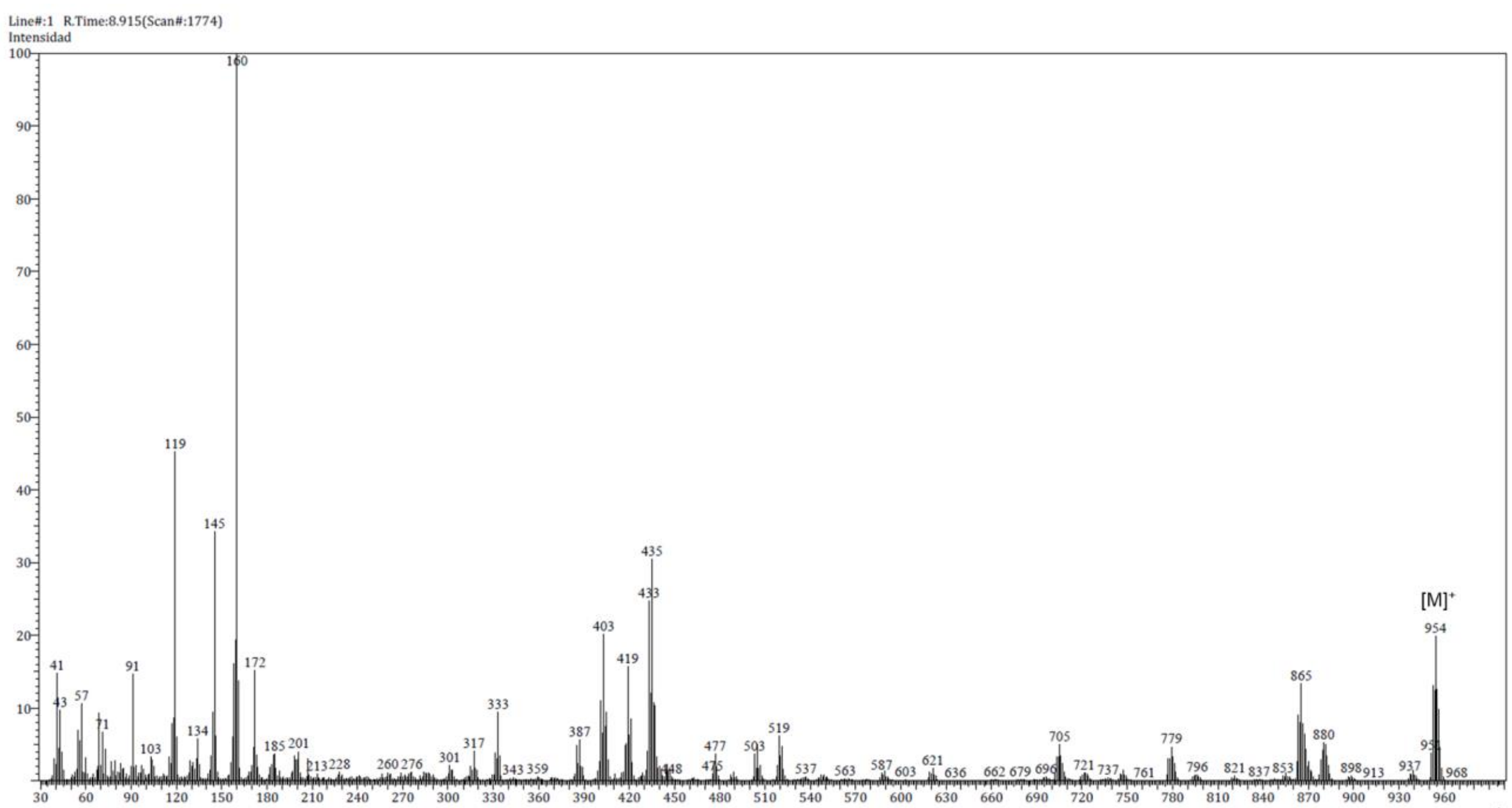

Figure S30. EI-MS spectrum of **17**.

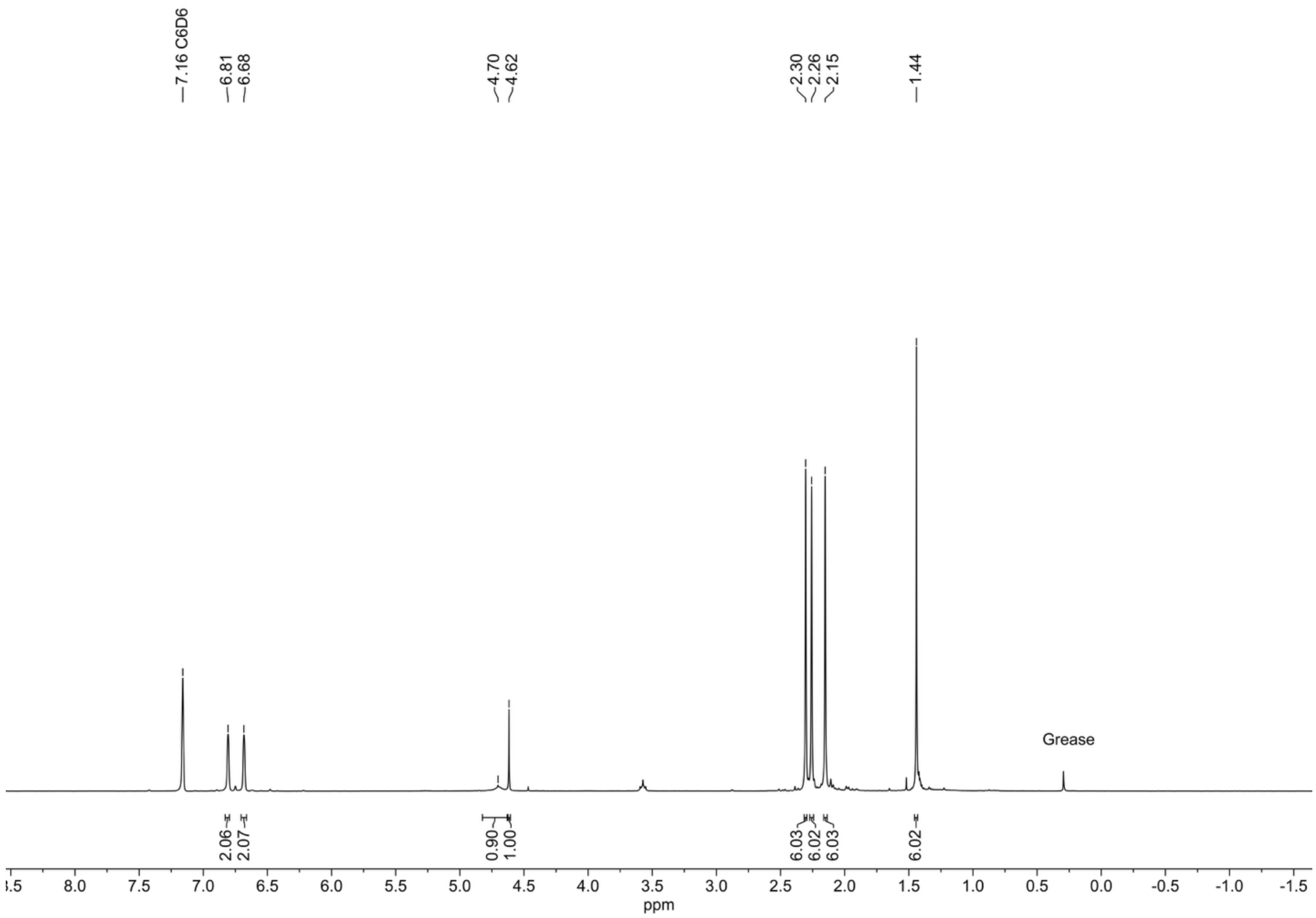

Figure S31. ^{1}H NMR spectrum of **18** in C_6D_6.

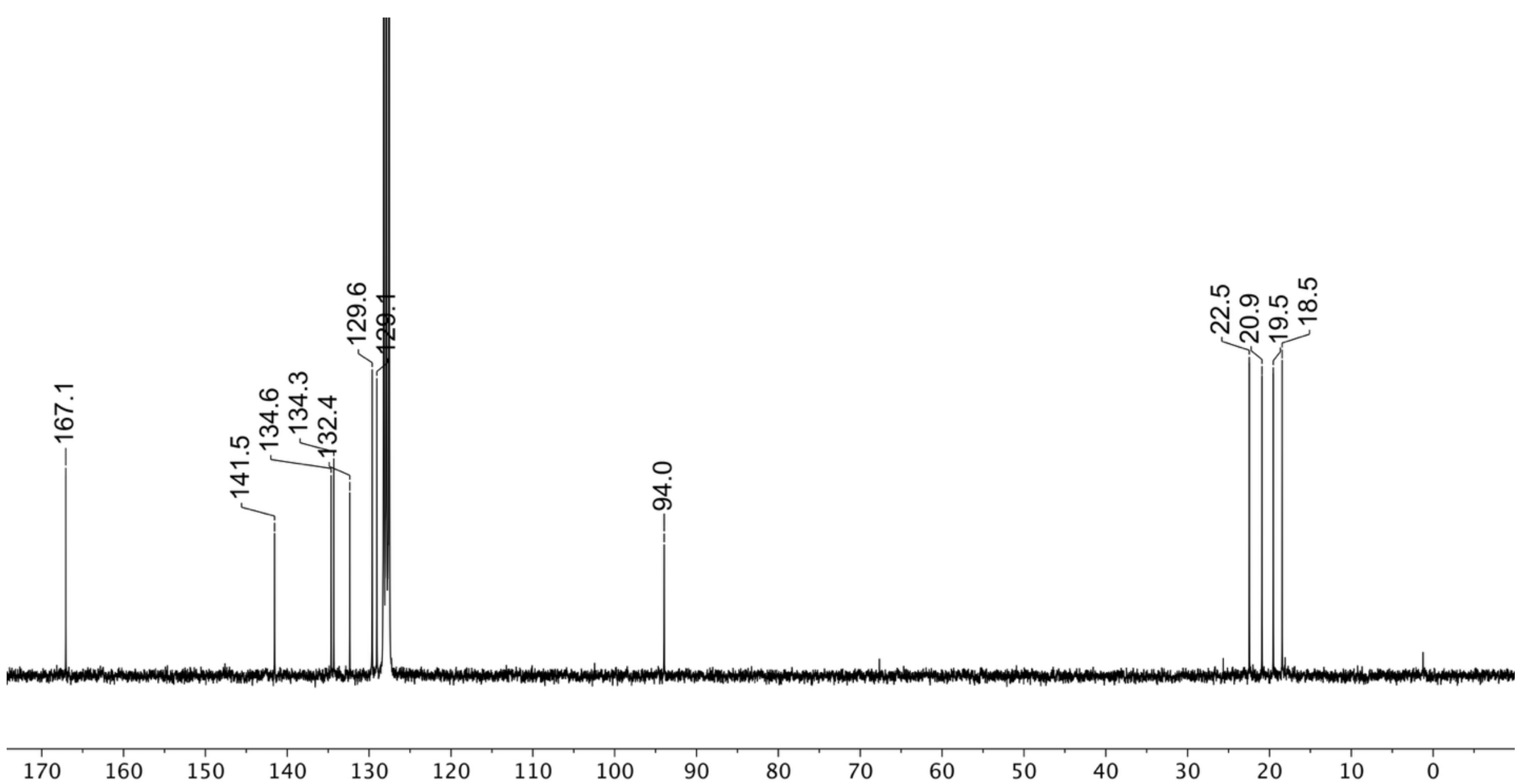

Figure S32. ^{13}C NMR spectrum of **18** in C_6D_6.

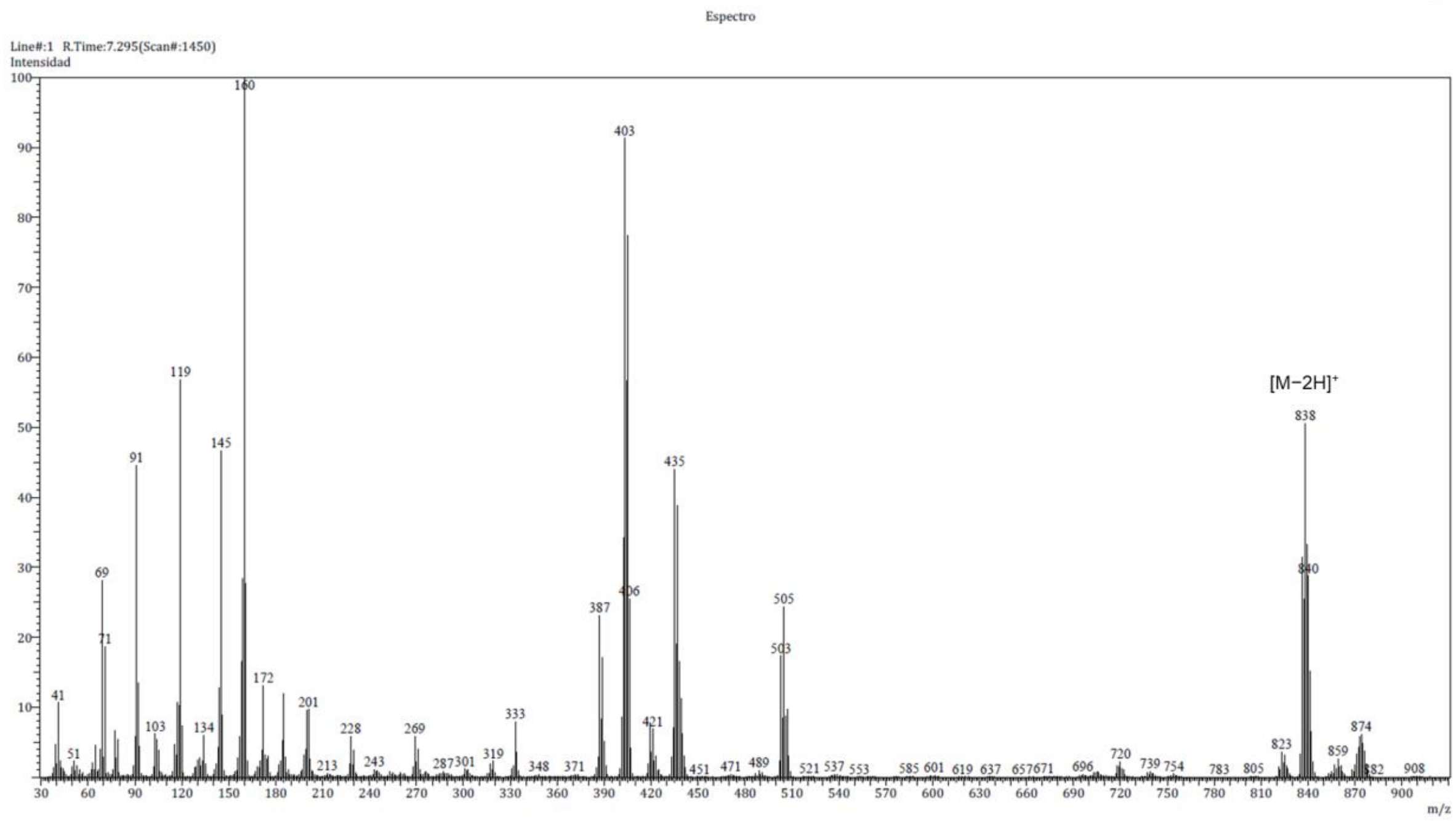

Figure S33. EI-MS spectrum of **18**

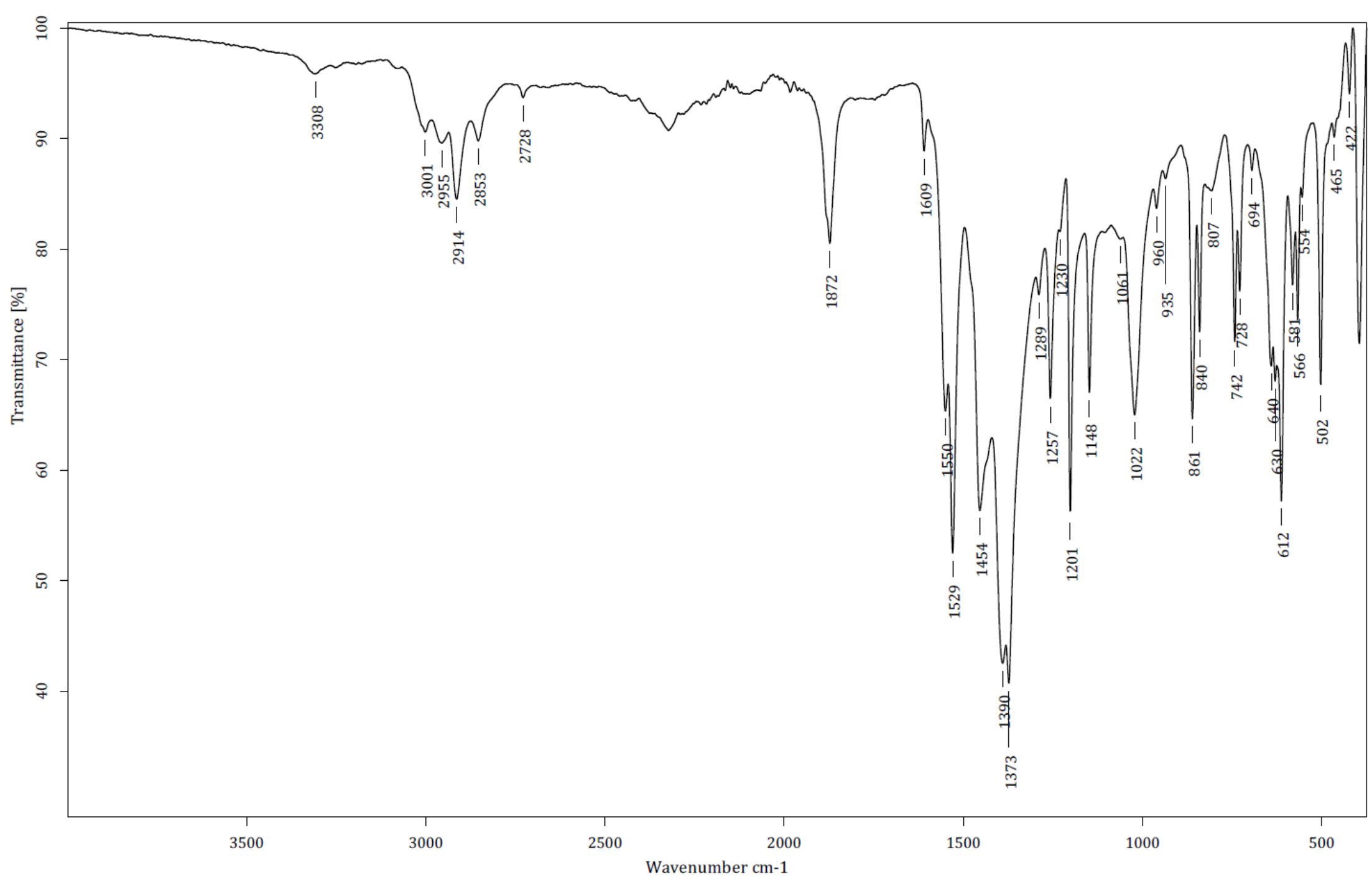

Figure S34. IR (ATR) spectrum of **18**.

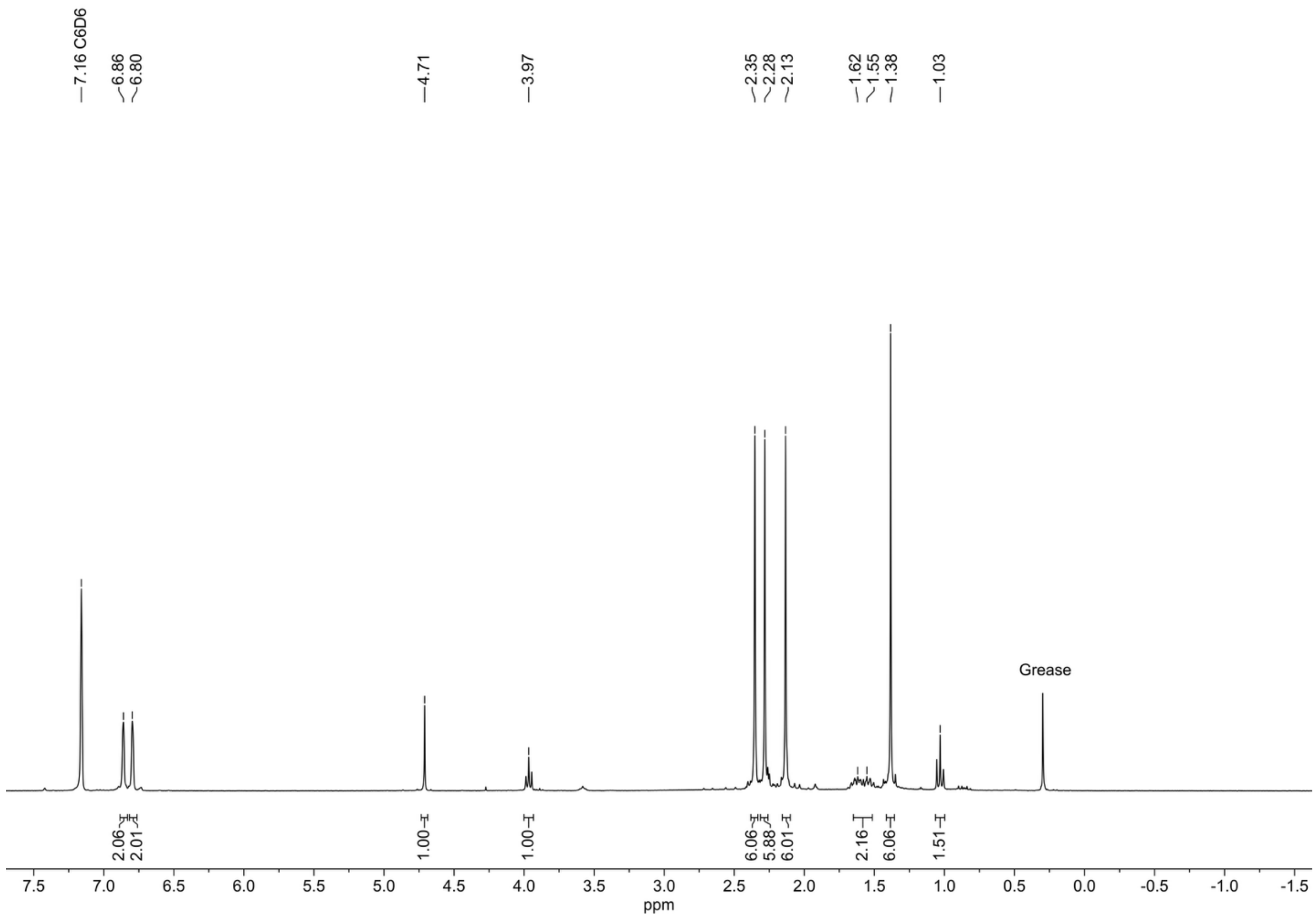

Figure S35. ^{1}H NMR spectrum of **19** in C_6D_6.

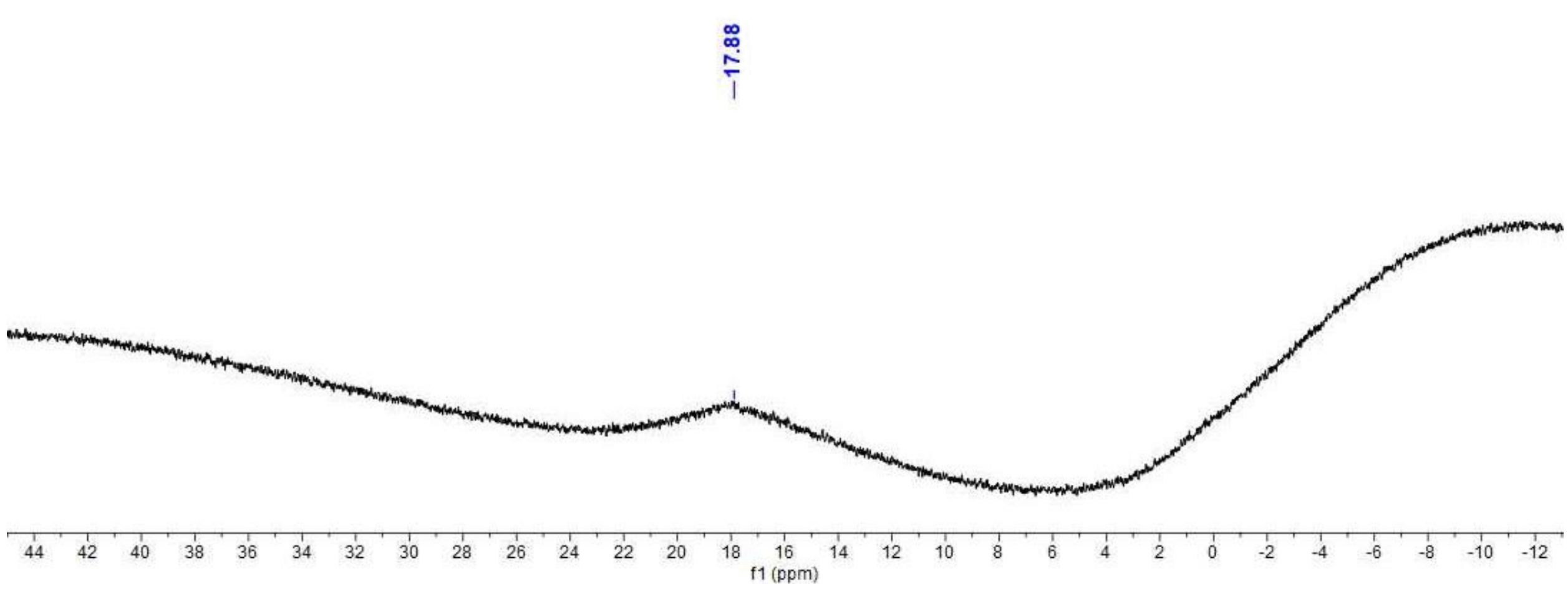

Figure S36. ^{11}B NMR spectrum of **19** in C_6D_6.

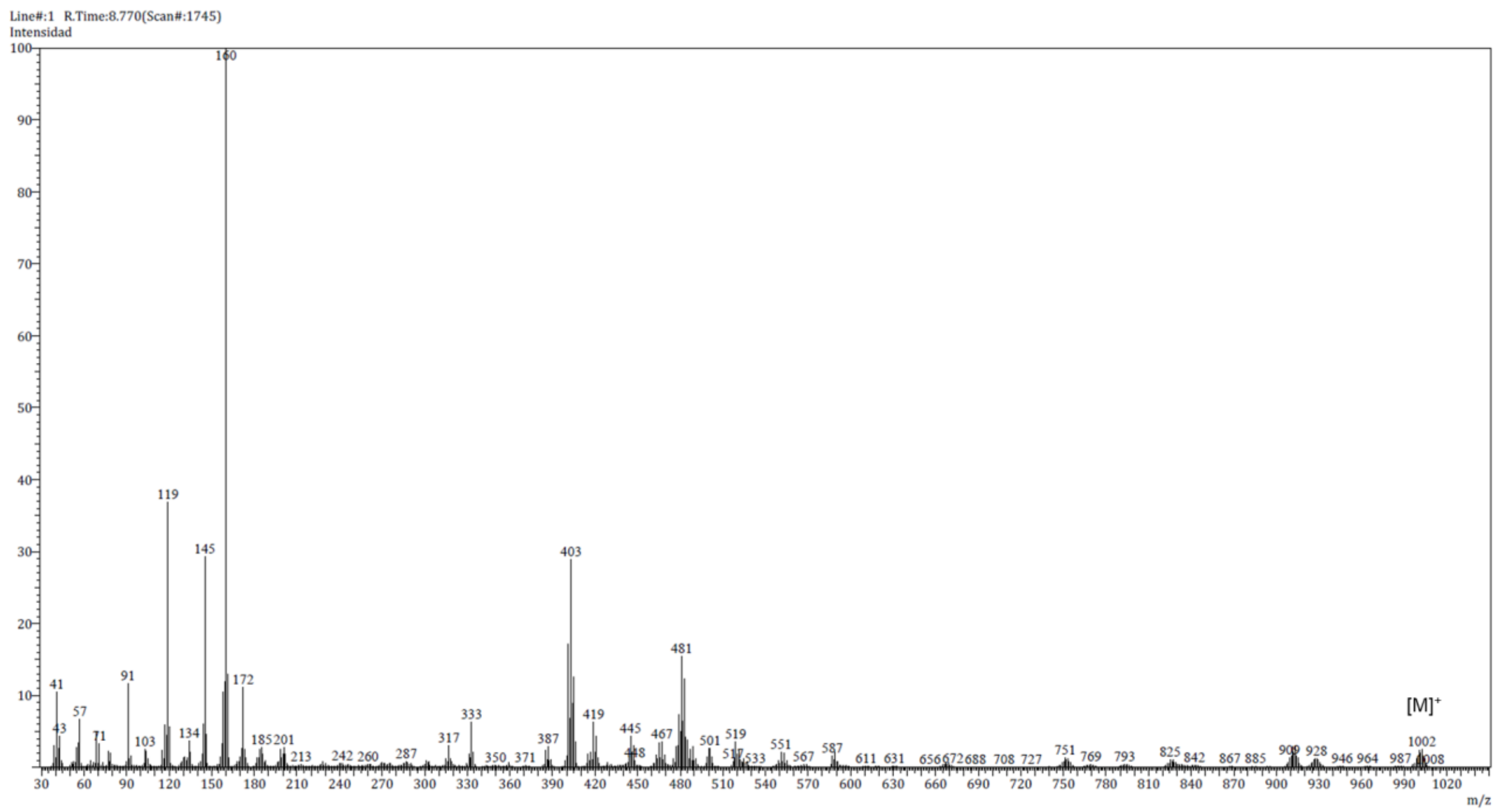

Figure S37. EI-MS spectrum of **19**.

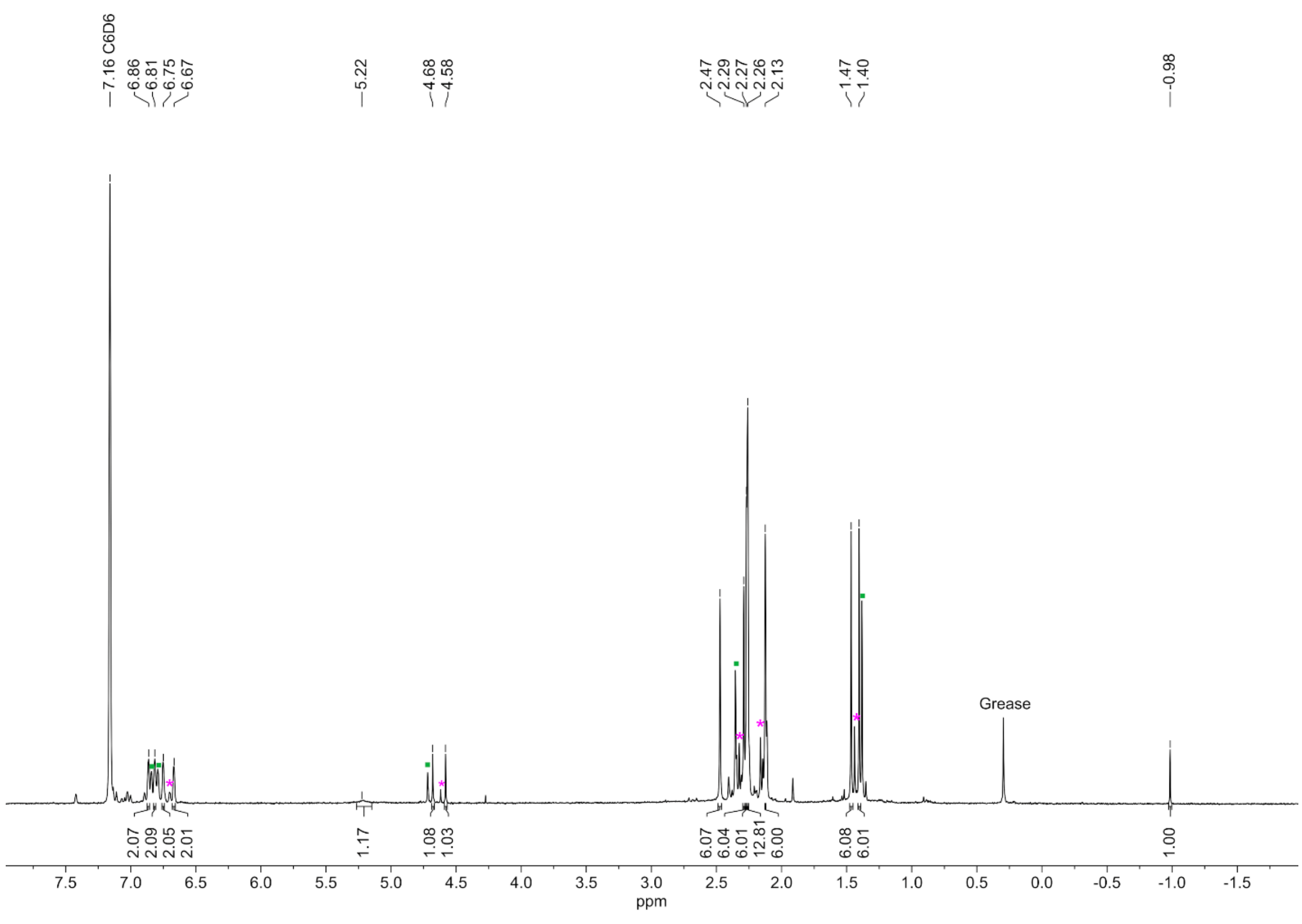

Figure S38. ^{1}H NMR spectrum of **20** in C_6D_6 with traces of **20a** (pink) and **20b** (green).

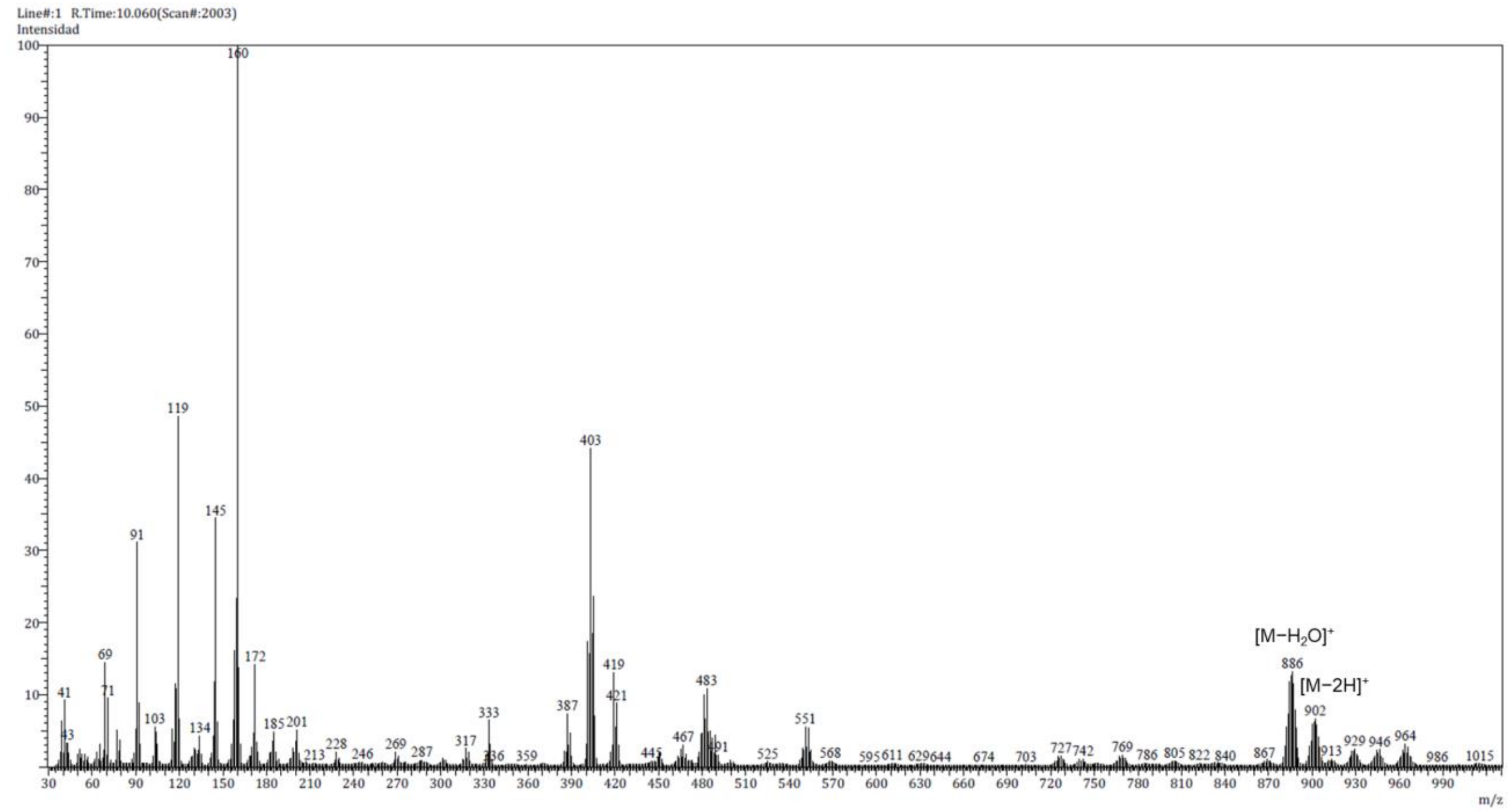

Figure S39. EI-MS spectrum of **20**.

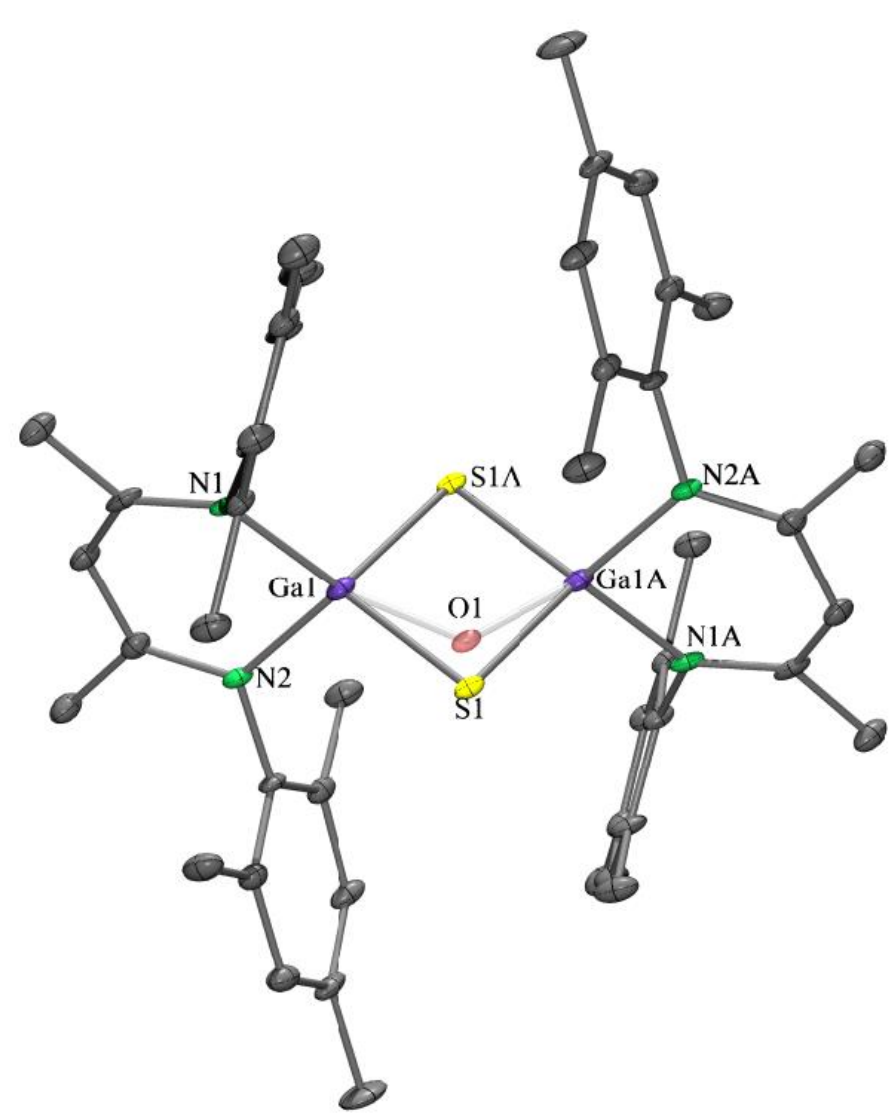

Figure S40. Molecular structure of **7** with thermal ellipsoids at 50% probability. Hydrogen atoms were omitted for clarity. Selected Bond Lengths (Å) and Angles (º): Ga(1)–S 2.245(1) and 2.269(1), Ga(1)–O(1) 1.720(3), Ga(1)–N 1.952(3) and 1.945(3), Ga(1)–S(1)–Ga(1A) 79.4(1), Ga(1)–O(1)–Ga(1A) 114.0(3), S(1)–Ga(1)–S(1A) 100.6(1).

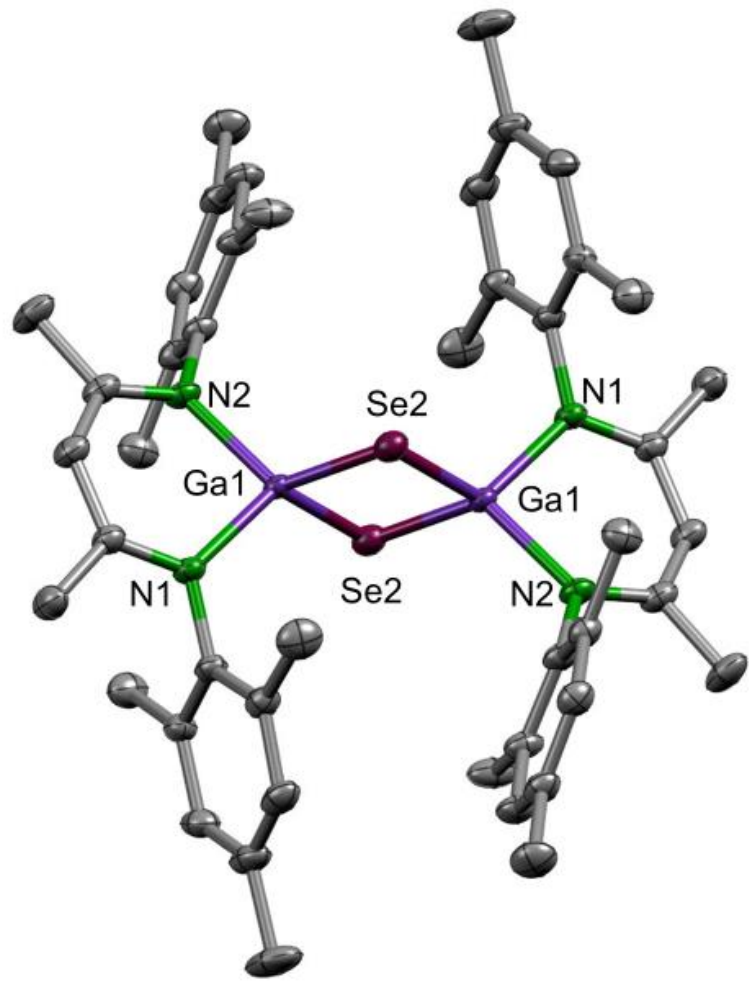

8

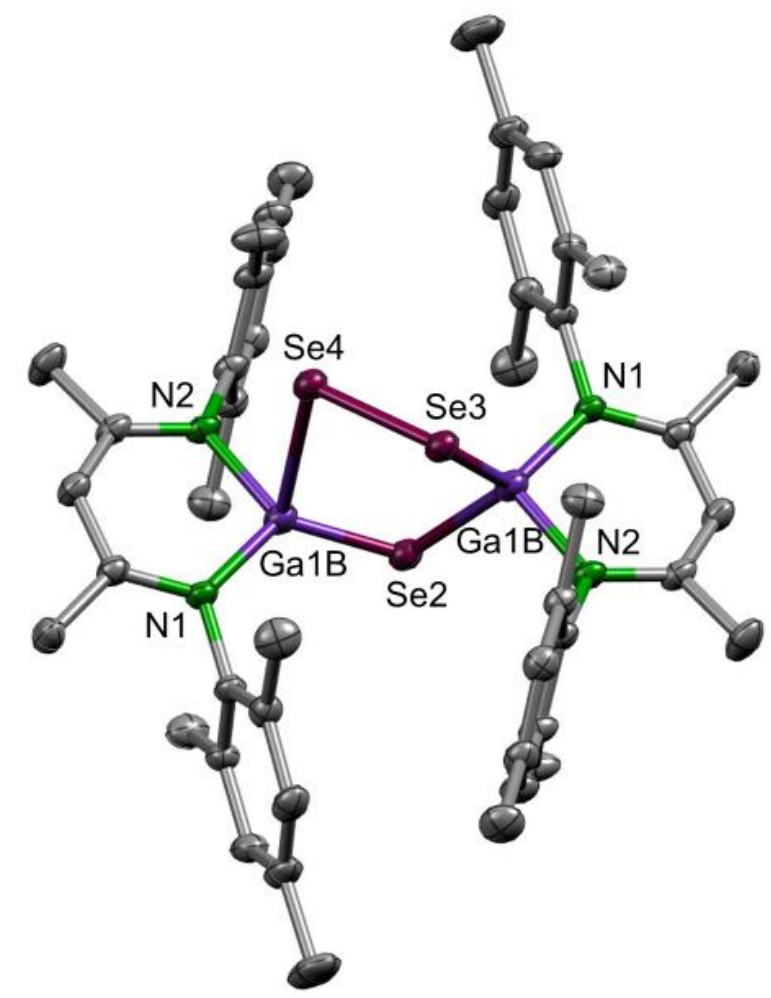

8a

Figure S41. Molecular structures of **8** and **8a** with thermal ellipsoids at 50% probability. Hydrogen atoms were omitted for clarity.

Table S1. Crystallographic Data and Refinement Details for Compounds **2**–**7**, **8** and **8a**.

	**2**·benzene	**3**	**4**	**5, 8, 8a**	**6**	**7**
Chemical formula	$C_{52}H_{64.13}Cl_{1.87}Ga_2N_4O_{0.13}S$	$C_{46}H_{62}Ga_2N_6S$	$C_{46}H_{60}Ga_2N_4O_2S$	$C_{46}H_{58.23}Ga_2N_4O_{0.23}Se_{1.89}$	$C_{46}H_{62}Ga_2N_6O$	$C_{46}H_{58}Ga_2N_4O_{0.17}S_{1.83}$
Formula weight	985.05	870.51	872.48	959.60	854.45	867.74
Space group	*C*2/*c*	$P\bar{1}$	$P\bar{1}$	$P\bar{1}$	*Fdd*2[c]	$P\bar{1}$
a, Å	22.0782(5)	11.4858(2)	11.3897(3)	8.6002(3)	20.5045(3)	8.5391(7)
b, Å	9.2277(2)	12.5158(2)	12.5050(3)	10.0926(3)	47.6325(8)	10.0160(8)
c, Å	24.7772(5)	16.8853(3)	16.7599(5)	13.4435(4)	9.11740(10)	13.4610(10)
α, deg	90	77.0729(6)	76.8498(13)	70.1792(6)	90	70.894(2)
β, deg	97.7686(4)	71.3337(5)	72.4877(13)	82.7322(6)	90	82.099(2)
γ, deg	90	86.8168(6)	86.8925(13)	79.8768(6)	90	79.629(2)
V, Å^3	5001.55(19)	2241.03(7)	2216.49(10)	1077.83(6)	8904.8(2)	1066.27(15)
Z	4	2	2	1	8	1
temp, K	100(2)	100(2)	100(2)	100(2)	100(2)	100(2)
λ, Å	0.71073	0.71073	0.71073	0.71073	1.54178	0.71073
μ, mm^{-1}	1.257	1.287	1.303	2.882	1.788	1.390
ρ_{calc} /g·cm^{-3}	1.308	1.290	1.307	1.478	1.275	1.351
R_1 ($I > 2\sigma(I)$)[a]	0.0242	0.0267	0.0261	0.0297	0.0212	0.0521
wR_2 (all data)[b]	0.0639	0.0715	0.0684	0.0739	0.0581	0.0681

[a] $R_1 = \sum||F_o| - |F_c||/\sum|F_o|$. [b] $wR_2 = [\sum w(F_o^2 - F_c^2)^2/\sum(F_o^2)^2$. [c] Absolute structure parameter Flack 0.014(6).

Table S2. Selected Bond Lengths (Å) and Angles (deg) for Compounds **2** and **6**.

**2**·benzene		**6**	
Ga(1)–S(1)	2.1958(4)	Ga(1)–O(1)	1.8180(1)
Ga(1)–Cl(1)	2.1846(6)	Ga(1)–N(1)	1.9688(18)
Ga(1)–N(1)	1.9315(13)	Ga(1)–N(2)	1.957(2)
Ga(1)–N(2)	1.9379(13)	Ga(1)–N(3)	1.878(2)
Ga(1)-S(1)-Ga(1)#1	107.24(3)	Ga(1)-O(1)-Ga(1)#2	119.06(15)
N(1)-Ga(1)-N(2)	97.05(11)	N(1)-Ga(1)-N(2)	95.27(8)

#1 -x+1, y, -z+1/2
#2 -x+5/2, -y+3/2, -z

Table S3. Selected Bond Lengths (Å) and Angles (deg) for Compounds **3**–**5**.

**3**		**4**		**5**	
Ga(1)–S(1)	2.2428(4)	Ga(1)–S(1)	2.2162(5)	Ga(1A)–Se(2)	2.337(4)
Ga(2)–S(1)	2.2181(4)	Ga(2)–S(1)	2.2226(5)	Ga(1B)–Se(2)	2.351(4)
Ga(1)–N(5)	1.8663(15)	Ga(1)–O(1)	1.8151(13)	Ga(1A)–O(1)	1.817(19)
Ga(2)–N(6)	1.8723(14)	Ga(2)–O(2)	1.8142(14)	Ga(1B)–O(2)	1.778(18)
Ga(1)–N(1)	1.9555(13)	Ga(1)–N(1)	1.9439(15)	Ga(1B)–N(1)	1.802(4)
Ga(1)–N(2)	1.9708(13)	Ga(1)–N(2)	1.9551(15)	Ga(1B)–N(2)	1.893(4)
Ga(2)–N(3)	1.9661(13)	Ga(2)–N(3)	1.9472(15)	Ga(1A)-Se(2)-Ga(1B)	95.44(18)
Ga(2)–N(4)	1.9663(14)	Ga(2)–N(4)	1.9440(14)	N(1)-Ga(1B)-N(2)	103.5(2)
Ga(1)-S(1)-Ga(2)	100.37(2)	Ga(1)-S(1)-Ga(2)	101.80(2)		
N(1)-Ga(1)-N(2)	96.71(6)	N(1)-Ga(1)-N(2)	96.02(6)		
N(3)-Ga(2)-N(4)	94.98(6)	N(3)-Ga(2)-N(4)	97.66(6)		

Table S4. Crystallographic Data and Refinement Details for Compounds **9**, **12**, **15**, **16** and **17**.

	**9**	**12**	**15**	**16**·4 pentane	**17**·benzene
Chemical formula	$C_{50}H_{70}Ga_2N_6O_2STi$	$C_{92}H_{116}Ga_4N_8O_4S_2Ti$	$C_{47.97}H_{63.90}Ga_2N_4O_{1.97}Se_{1.02}Si_{0.98}$	$C_{204}H_{288}Ga_8N_{16}O_{18}S_4Zr_5$	$C_{56}H_{73}BGa_2N_4O_3S$
Formula weight	1006.52	1788.82	975.33	4394.59	1032.49
Space group	$P\bar{1}$	*Pbca*	$P\bar{1}$	*Cc*[c]	$P\bar{1}$
a, Å	11.0598(3)	15.4404(2)	10.54080(10)	26.7760(5)	10.3084(2)
b, Å	13.3930(3)	28.3712(4)	13.5185(2)	25.3692(5)	13.3707(3)
c, Å	18.5593(5)	41.2665(5)	18.6980(2)	34.1926(7)	20.9209(5)
α, deg	70.0071(11)	90	75.8165(5)	90	81.7476(7)
β, deg	81.7509(12)	90	75.1570(5)	110.4082(8)	84.0801(7)
γ, deg	77.0519(12)	90	68.6851(4)	90	69.7542(7)
V, Å^3	2511.00(11)	18077.3(4)	2364.92(5)	21768.7(7)	2672.93(10)
Z	2	8	2	4	2
temp, K	100(2)	100(2)	100(2)	100(2)	100(2)
λ, Å	0.71073	1.54178	0.71073	0.71073	0.71073
μ, mm^{-1}	1.306	2.901	1.987	1.299	1.093
ρ_{calc} /g·cm^{-3}	1.331	1.315	1.370	1.341	1.283
R_1 ($I > 2\sigma(I)$)[a]	0.0282	0.0322	0.0239	0.0319	0.0326
wR_2 (all data)[b]	0.0734	0.0850	0.0608	0.0754	0.0805

[a] $R_1 = \sum||F_o| - |F_c||/\sum|F_o|$. [b] $wR_2 = [\sum w(F_o^2 - F_c^2)^2/\sum(F_o^2)^2]^{1/2}$. [c] Absolute structure parameter Flack 0.128(5), refined as inversion twin.

Table S5. Selected Bond Lengths (Å) and Angles (deg) for Compounds **9**, **12**, **15** and **17**.

	**9**	**15**	**17**·benzene	**12**	
Ga(1)–E(1)	2.2340(6)	2.3521(2)	2.1942(5)	Ga(1)–S(1)	2.2096(5)
Ga(2)–E(1)	2.2133(5)	2.3253(2)	2.2271(5)	Ga(2)–S(1)	2.2290(6)
Ga(1)–O(1)	1.8144(14)		1.8172(14)	Ga(3)–S(2)	2.2290(5)
Ga(2)–O(2)	1.8125(14)		1.8243(14)	Ga(4)–S(2)	2.2084(5)
A(1)–O(1)	1.7839(14)	1.6173(12)	1.351(3)	Ga(1)–O(1)	1.8177(13)
A(1)–O(2)	1.8075(15)	1.6224(12)	1.354(3)	Ga(2)–O(2)	1.8124(14)
A(1)–R	1.9129(19)<br>1.922(3)	1.8650(19)<br>1.8590(19)	1.393(3)	Ga(3)–O(3)	1.8115(14)
Ga(1)–N(1)	1.9474(17)	1.9417(13)	1.9316(16)	Ga(4)–O(4)	1.8202(14)
Ga(1)–N(2)	1.9477(17)	1.9415(13)	1.9409(16)	Ti(1)–O(1)	1.8127(14)
Ga(2)–N(3)	1.9463(17)	1.9399(13)	1.9378(17)	Ti(1)–O(2)	1.8195(14)
Ga(2)–N(4)	1.9495(17)	1.9522(13)	1.9312(17)	Ti(1)–O(3)	1.8140(14)
Ga(1)-E(1)-Ga(2)	105.92(2)	97.757(8)	96.72(2)	Ti(1)–O(4)	1.8170(14)
O(1)-Ga(1)-E(1)	116.51(5)		118.15(5)	Ga(1)-S(1)-Ga(2)	103.17(2)
O(2)-Ga(2)-E(1)	117.81(5)		116.46(5)	Ga(3)-S(2)-Ga(4)	103.23(2)
O(1)-A(1)-O(2)	109.25(7)	112.16(6)	126.5(2)	O(1)-Ti(1)-O(2)	103.64(6)
R-A(1)-R	111.3(6)	107.53(10)		O(3)-Ti(1)-O(4)	103.94(6)

E = S; A = Ti, R = NMe_2 (**9**), A = B, R = O^nBu (**17**). E = Se, A = Si, R = Me (**15**)

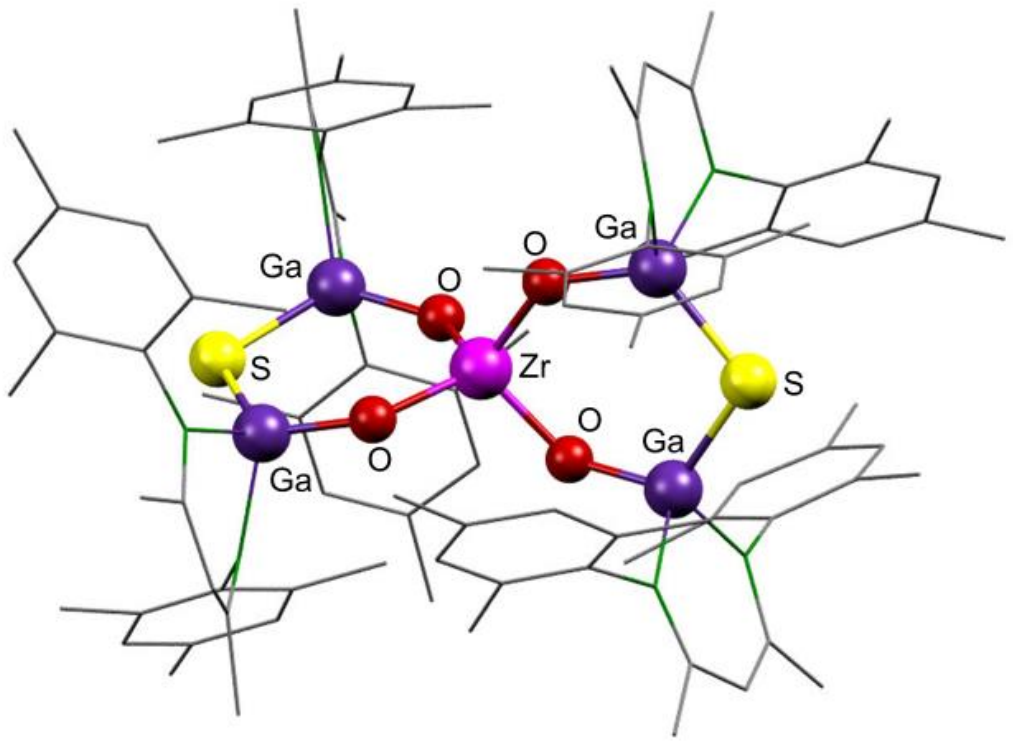

13

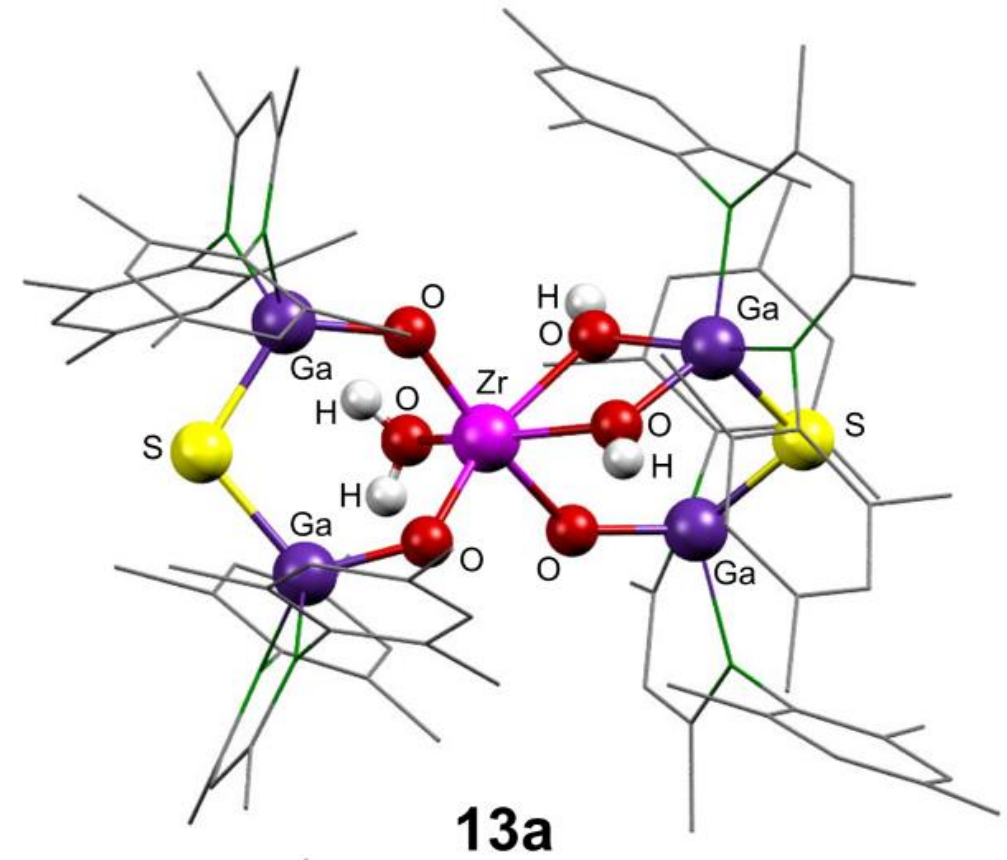

13a

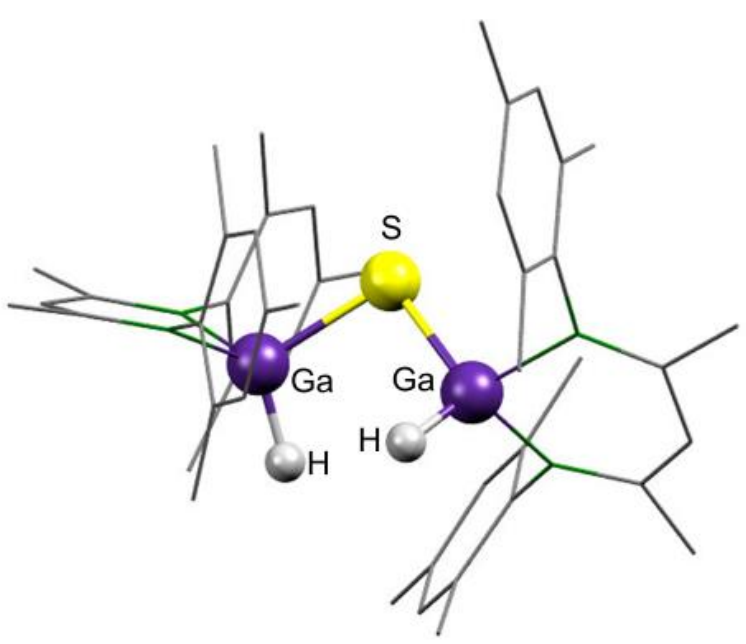

18

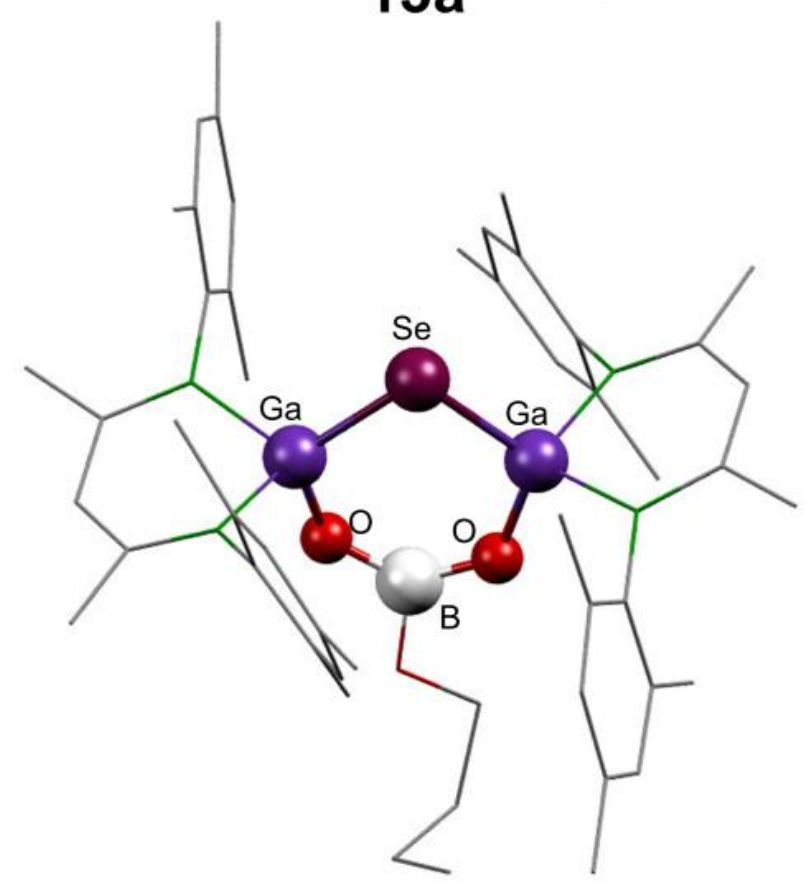

19

Figure S42. Ball-and-stick diagram of connectivity model for **13**, **13a**, **18** and **19** (ligand units and O^{n}Bu group are displayed as wireframe). Hydrogen atoms were omitted for clarity, except those from functional groups in **13a** and **18**.

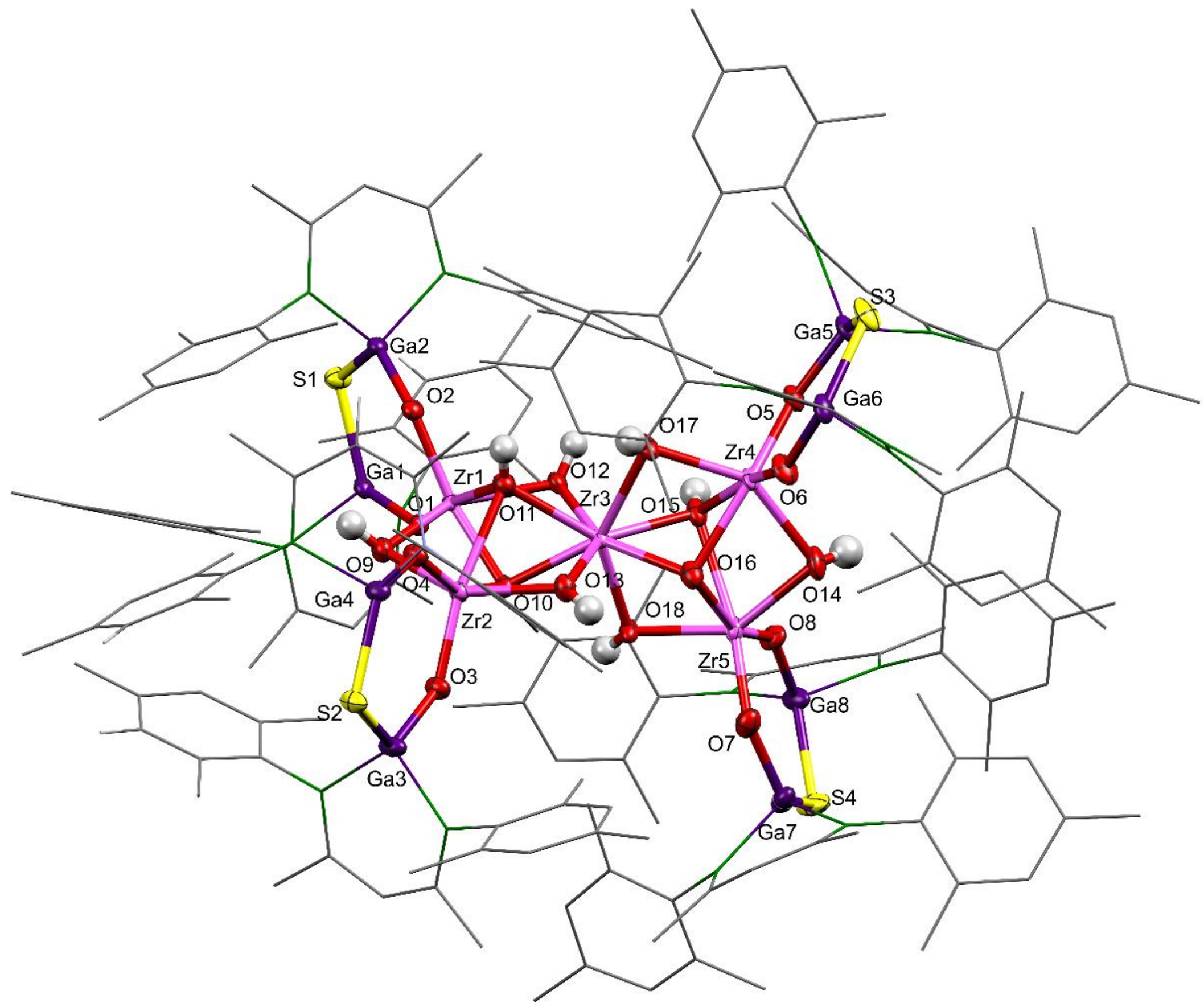

Figure S43. Molecular structure for **16** with thermal ellipsoids at 30% probability (ligand units are displayed as wireframe). Hydrogen atoms (except for μ-OH) and solvent molecules were omitted for clarity.

Table S6. Selected Bond Lengths (Å) and Angles (deg) for Compound **16**.

Zr(1)–O(9)	2.189(3)	Zr(1)–Zr(2)	3.2755(6)	Ga(1)-S(1)-Ga(2)	103.26(5)
Zr(1)–O(10)	2.114(3)	Zr(1)–Zr(3)	3.2812(5)	Ga(3)-S(2)-Ga(4)	105.61(5)
Zr(1)–O(11)	2.398(3)	Zr(2)–Zr(3)	3.2544(5)	Ga(5)-S(3)-Ga(6)	104.93(6)
Zr(1)–O(12)	2.138(3)	Zr(3)–Zr(4)	3.2684(17)	Ga(5A)-S(3)-Ga(6)	97.69(15)
Zr(2)–O(9)	2.150(3)	Zr(3)–Zr(5)	3.2551(5)	Ga(7)-S(4)-Ga(8)	105.08(5)
Zr(2)–O(10)	2.142(3)	Zr(4)–Zr(5)	3.3063(15)	O(1)-Zr(1)-O(2)	98.11(13)
Zr(2)–O(11)	2.368(3)	Ga(1)–S(1)	2.1976(12)	O(3)-Zr(2)-O(4)	99.48(13)
Zr(2)–O(13)	2.124(3)	Ga(2)–S(1)	2.2256(12)	O(5)-Zr(4)-O(6)	98.23(18)
Zr(3)–O(10)	2.151(3)	Ga(3)–S(2)	2.2394(12)	O(7)-Zr(5)-O(8)	98.84(14)
Zr(3)–O(11)	2.275(3)	Ga(4)–S(2)	2.2160(12)		
Zr(3)–O(12)	2.201(3)	Ga(5)–S(3)	2.2188(15)		
Zr(3)–O(13)	2.182(3)	Ga(5A)–S(3)	2.258(5)		
Zr(3)–O(15)	2.261(3)	Ga(6)–S(3)	2.2410(14)		
Zr(3)–O(16)	2.161(3)	Ga(7)–S(4)	2.2074(16)		
Zr(3)–O(17)	2.231(3)	Ga(8)–S(4)	2.2343(14)		
Zr(3)–O(18)	2.161(3)	Ga(1)–O(1)	1.818(3)		
Zr(4)–O(14)	2.215(4)	Ga(2)–O(2)	1.798(3)		
Zr(4)–O(15)	2.274(4)	Ga(3)–O(3)	1.800(3)		
Zr(4)–O(16)	2.193(4)	Ga(4)–O(4)	1.799(3)		
Zr(4)–O(17)	2.120(4)	Ga(5)–O(5)	1.801(4)		
Zr(5)–O(14)	2.188(3)	Ga(6)–O(6)	1.792(3)		
Zr(5)–O(15)	2.445(4)	Ga(7)–O(7)	1.807(4)		
Zr(5)–O(16)	2.120(3)	Ga(8)–O(8)	1.799(3)		
Zr(5)–O(18)	2.092(3)				

3. COMPUTATIONAL DETAILS

Computational Methods

DFT geometry optimizations of the gallium chalcogenide dihydroxides [$\{^{Me}LGa(OH)\}_2(\mu\text{-}E)$] (E = O (**I**), S (**4**), Se (**5**)) were performed using the electronic structure package ORCA v. 5.0.3.[5] The chosen basis set was Def-TZVPP for N, O, Ga, S, Se atoms and the organic backbone (C, H) was modeled at the doble-ζ level with the Def2-SVP basis set.[6] The solvated species were modeled within the conductor-like polarizable continuum model (CPCM)[7] selecting toluene as solvent with the CAM-B3LYP functional. Non-covalent Interactions (NCI)[8] were examined with the program NCIPLOT4.[9] The required wfx files were produced with the *orca_2aim* utility using the gbw file obtained via a single-point calculation at the ωB97X-D3BJ/Def2-TZVP level.

Non-Covalent Interactions (NCI) analysis for hydrogen bond formation

The molecular structures obtained by X-ray diffraction studies revealed no hydrogen bond interaction between the OH groups in **I**, while the OH groups in **4** present a *syn* conformation, and in **5** there is a hydrogen bond interaction between the hydroxides. The NCI calculations were performed to analyze this observation, and the NCI surfaces obtained are presented in **Figure S44**. Compound **I** exhibited weak van der Waals (VdW) interactions between the OH groups, whereas hydrogen bonding was observed in compounds **4** and **5** (**Figure S44a**). The strength of the H–O···H bonds follows the order E = O (**I**) < S (**4**) < Se (**5**), as evidenced by the higher negative λ_2 values in the NCI plots (**Figure S44b**).

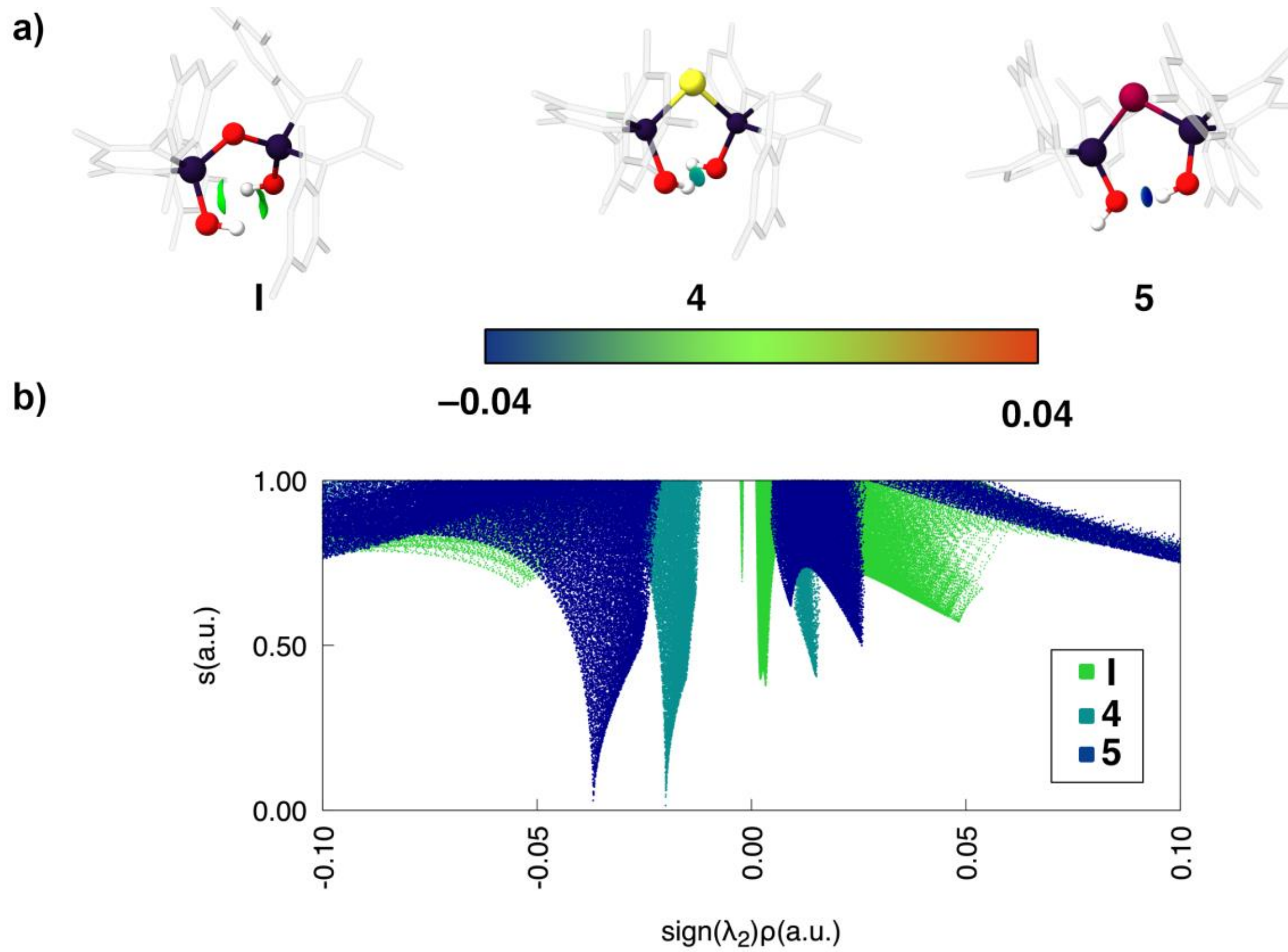

Figure S44. **a)** NCI surfaces at s = 0.4 a.u. for **4** and **5**, and 0.5 a.u. for **I**, color scale of –0.04 < ρ < 0.04. Interactions were localized in space using the RADIUS option; **b)** Overlap of s(r) vs sign(λ_2)ρ plots, illustrating the strengthening of O–H···O interaction.

4. REFERENCES

(1) SAINT and SADABS; Bruker AXS Inc.: Madison, WI, 2007.

(2) SHELX: Sheldrick, G. M. A Short History of SHELX. *Acta Crystallogr., Sect. C: Struct. Chem.* ***2015***, 71, 3−8.

(3) SHELXLE: Hübschle, C. B.; Sheldrick, G. M.; Dittrich, B. ShelXle: a Qt Graphical User Interface for SHELXL. *J. Appl. Crystallogr.* **2011**, *44*, 1281−1284.

(4) Taylor, W. L.; Griswold, E.; Kleinberg, J. Reactions of Some Alkali and Alkaline Earth Metals with Aluminum(III) Iodide in Liquid Ammonia**.** *J. Am. Chem. Soc.* **1955**, *77*, 294−298.

(5) a) Neese, F. The ORCA Program System. *WIREs Comput. Mol. Sci.* **2012**, *2*, 73–78. b) Neese, F. Software Update: The ORCA Program System, Version 4.0. WIREs *Comput. Mol. Sci.* **2018**, *8*, e1327. c) Neese, F. Software Update: The ORCA Program System—Version 5.0. WIREs. *Comput. Mol. Sci.* **2022**, *12*, e1606.

(6) Weigend, F.; Ahlrichs, R. Balanced Basis Sets of Split Valence, Triple Zeta Valence andQuadruple Zeta Valence Quality for H to Rn: Design and Assessment of accuracy. *Phys. Chem. Chem. Phys.***2005**,*7*, 3297–3305.

(7) Barone, V.; Cossi, M. Quantum Calculation of Molecular Energies and Energy Gradients inSolution by a Conductor Solvent Model. *J. Phys. Chem. A* **1998**, *102*, 1995–2001

(8) a) Contreras-García, J.; Johnson, E. R.; Keinan, S.; Chaudret, R.; Piquemal, J.-P.; Beratan, D. N.; Yang, W. NCIPLOT: A Program for Plotting Noncovalent Interaction Regions. *J. Chem. Theory Comput.* **2011**, *7*, 625–632. b) Boto, R. A.; Peccati, F.; Laplaza, R.; Quan, C.; Carbone, A.; Piquemal, J.-P.; Maday, Y.; Contreras-García, J. NCIPLOT4: Fast, Robust, and Quantitative Analysis of Noncovalent Interactions. *J. Chem. Theory Comput.* **2020**, *16*, 4150–4158.

(9) Chai, J.; Head-Gordon, M. Long-Range Corrected Hybrid Density Functionals with Damped Atom – Atom Dispersion Corrections. *Phys. Chem. Chem. Phys.* **2008**, *10*, 6615–6620.
